# Optimizing foreign exchange reserves: Protection against external shocks in Ghana

**DOI:** 10.3389/fpsyg.2022.994043

**Published:** 2022-11-02

**Authors:** Abdul-Rashid Abdul-Rahaman, Yao Hongxing, Abdul-Rasheed Alhassan Alolo Akeji, Emmanuel Caesar Ayamba, Jean Baptiste Bernard Pea-Assounga, Mohammed Kamil Alhassan

**Affiliations:** ^1^School of Finance and Economics, Jiangsu University, Zhenjiang, China; ^2^Department of Accounting and Finance, School of Business Studies, Bolgatanga Technical University, Sumbrungu, Ghana; ^3^School of Finance and Economics, Jiangsu University, Zhenjiang, China; ^4^Department of Logistics and Procurement Management, Tamale Technical University, Tamale, Ghana; ^5^Department of Procurement, School of Business Studies, Bolgatanga Technical University, Sumbrungu, Ghana; ^6^School of Finance and Economics, Jiangsu University, Zhenjiang, China; ^7^Department of Mathematics, Bilkent University, Ankara, Turkey

**Keywords:** Bank of Ghana, foreign exchange reserves, central banking, monetary policy, optimality, least squared residuals

## Abstract

Using Least Square Residual Minimization techniques, this paper develops an optimal reserve model, known as the OPREM model, which is essential in optimizing the costs of reserve holding. The paper also sets-out to test and compare the relative predictions of economic trends of the OPREM model as well as the predictions of alternative models in literature. Establishing the predictive accuracy of economic trends of these models are crucial for the gradual and cost-effective accumulation of reserves. The research concludes that, the decision to optimize the cost of reserves under a stable currency environment is reliant on the gold impact factor and not on inflation or interest rates. We also found on further analysis of the OPREM that the OPREM model is better positioned to eliminate the procyclicality and perverse rush in reserve build-ups experienced in developing and emerging countries by effectively setting the reserve stock against economic trends. The research fixes the optimal reserves around a benchmark of 0.7–1.2 of previous year's optimal value. However, in the absence of past optimal values, a benchmark between 2 and 6 times of average inflows for short-term analysis or analysis with small data observations. However, for long-term analysis or analysis with large data frequency (i.e., exceeding 13 data observations), the reserve stock should be fixed on a benchmark of 2–9 times of the average inflows.

“Reserve models produce imprecise estimates of reserve stocks” (Ghana, 2017).

## Introduction

Research on assessing countries' reserve holdings has always been a relevant macro-financial indicator for the IMF and other world financial institutions (Abdul-Rashid and Yao, 2019). The country reports of these respective institutions seldom capture the reserve holdings of countries to demonstrate their preparedness and resilience to external shocks and exposures. These reports rely heavily on the research conclusions of Kindleberger (1961), Heller (1966), Ben-Bassat and Gottlieb (1992), Greenspan (1999), and Wijnholds and Kapteyn (2001), who use benchmark assessments and, occasionally, cost-benefit assessment of the reserve stock of countries. Though these previous works have contributed immensely to building the literature on the holding of reserves, the recent procyclicality observed in reserve build-ups in developing, and emerging countries, the absence of future growth projections in these models, and the presence of unrealistic assumptions have limited the usefulness of these models. These problems regarding the existing models have cast doubts on the effectiveness of reserve models in fixing optimal reserve stocks. Therefore, this research aims to develop a reserve model that optimizes the cost of holding reserves and assists in fixing reserve levels or estimates that are superior in terms of reduced errors in adjusting the reserve stock to economic trends.

The reserve model proposed in this research, henceforth known as the OPREM model, contributes significantly to the literature on reserves and has vast implications for central banks' management and investors. The OPREM model addresses and satisfies all three logics of holding reserves (i.e., liquidity, reasonability, and future projections) which existing reserve models partially address. Also, the better reserve adjustments of the OPREM model to changes in economic variables gives a long term view of the reserve stock instead of the Adhoc view of most of the reserve models. This advantage helps to avoid the rush and perverse accumulation of reserves in most developing and emerging countries. The Ghana (2017) proffers that the existing reserve models, especially the benchmark models, produce imprecise estimates of reserve stocks. This weakness comes as huge financial and economic costs to countries. Therefore, the absence of this type of research which seeks to re-engineer and mend the weaknesses in the previous reserve models, will spell disaster to many economies, especially developing and emerging economies whose needs for reserves are well documented in Roger (1993) and Abdul-Rashid and Yao (2019). Therefore, this research develops a reserve model that optimizes the cost of holding reserves and provides superior benchmarks in fixing reserve levels.

Heller (1966) pioneered the formulation and estimation of optimal reserves using cost-benefit analysis. Following his work was research works such as Hamada and Ueda (1977), Frenkel and Jovanovic (1981), Ben-Bassat and Gottlieb (1992), and Tule et al. (2015) expounding on similar concepts of assessing reserves based on costs-benefits analysis. These papers bear similarities in the way each model incorporates countries' risk exposure or risk level (π) in the reserve stocks. Also, the models are again similar in the way each of them views and interpret the cost of reserves (*C*_0_) as the consequence of a country running out of reserves. However, the way and manner in which each model handles and calculates these π and *C*_0_ create fundamental differences between the models or theories. These differences in the models can be seen in the works of two prominent researchers in reserve modeling, that is, Heller (1966) (henceforth known as the H-Models) and Ben-Bassat and Gottlieb (1992) (henceforth known as the B-G Models). The B-G models estimate the π from a cross-sectional analysis of borrowing countries that had defaulted from their external debt obligations and have had a renegotiation of their debts during the sample period. This approach introduces subjectivity and narrows the renegotiation of debts to the incapacity of the borrowing country. On the contrary, the H-Models derive the π from a symmetric random walk process. A simple random walk is symmetric if observations have a constant probability over time (Gujarati, 2004). Also, the H-Models summarize and depict the foregone GDP or aggregate output in an economy as representing *C*_0_. This approach of the H-Models in calculating *C*_0_ is also narrowing the scope of estimation compared to the approach in B-G Models. This approach, according to Clark (1970) and Ben-Bassat and Gottlieb (1992), limits the probable cost of defaults (π*C*_0_). In addition to these groups of reserve models, reserve models provide benchmarks against economic variables. Examples of such research works are Kindleberger (1961), Greenspan (1999), and Wijnholds and Kapteyn (2001). As essential as they are as start point for reserve stock analysis, these benchmark metrics give imprecise estimates of the reserve levels (Ghana, 2017). These imprecise estimates have led to a perverse rush in accumulating reserves in developing and emerging economies (Pina, 2015; Ghana, 2017). The OPREM model proposed in this research is to improve these models by eliminating the subjectivities embedded in the models. This objective is achieved by sticking to opportunity costs instead of probabilities as in previous models. Also fundamental to the OPREM model is the treatment of the reserve stock as a multi-faceted and multi-period fund that optimizes each component of the reserve stock separately. These are improvements made on previous reserve models.

Testing the developed research model, we found that during periods of currency stability, the cost of holding reserves for precautionary purpose is optimized by overlooking the prevailing interest and inflationary rates and only considering these two variables; (1) the percentage trade-offs between global gold prices and hard currency values, and (2) the economic value of gold, defined as the output of domestic currency in circulation divided by the amount of gold in reserves. Therefore, in a stable currency environment, the only random variable in the reserve model is the gold impact factor. Also, the research concludes that this OPREM model is better positioned to eliminate procyclicality in reserve build-ups by returning reduced errors in computations and producing better adjustments of the reserve stock to economic trends and conditions. We also find that the optimal reserves set by the OPREM model closely match the reserve level of the import metric. This finding shows that the reserves fixed by the 3-month import model are not entirely off the mark. However, it does not possess the precise and constant adjustments of the OPREM model, where reserves easily adjust to economic trends and conditions. In addition, this paper gives benchmark estimates of the OPREM values. A benchmark of 0.7-to−1.2 of previous year's optimal value is given when past optimal values are known. When the past optimal values are not already known, a benchmark between 2 and 6 times of the average inflows in an economy is recommended for short-term analysis or analysis with small data observations. However, for long-term analysis or analysis with large data frequency (i.e., exceeding 13 data observations), the reserve stock should be fixed on a benchmark of 2 to 9 times of the average inflows. A panel analysis of selected countries may be necessary to generalize these benchmarks, but this may not be necessary as IMF recommendation is for country-specific management of reserves. Lastly, the research reveals that the regression properties of the OPREM model are capable of being exploited to show banking and financial sector weaknesses in the simulated countries. This information helps with a quantitative approach to determining financial and banking sector weaknesses in an economy.

This research is organized into 5 sections. Section Introduction introduces optimal reserves, and the previous research works carried out in this area. Section Literature review present the theoretical and empirical literature. Section Theoretical model and hypothesis development explains and expounds on the theoretical reserve model proposed in this work and highlights the assumptions made in the derivatives. Section Specification of an econometric model specifies an econometric model with the optimal reserve results of the proposed model in section Theoretical model and hypothesis development. Section Results and discussions presents the research results and discussions. Section Conclusions summarizes the conclusions of the study and gives policy recommendations to economic agents and policymakers.

## Literature review

This section analyses the theoretical and empirical literature of reserve models.

### Theoretical literature

Foreign exchange reserves are assets held by monetary authorities in foreign currencies, including foreign bank deposits, treasury bills, short-term and long-term foreign government securities, gold reserves, special drawing rights, and the international monetary fund reserve positions. These assets support countries' liabilities and have become essential to monetary policies in open and liberalized economies (Roger, 1993; Assessing Reserve Adequacy—Specific Proposals, 2014; Abdul-Rashid and Yao, 2019). The knowledge of reserves, i.e., how to constituent the reserve stock and the thresholds, is more beneficial for developing countries with less developed financial markets to provide the needed liquidity to support businesses and the economy during crises (Roger, 1993; Hongxing and Rahaman, 2018).

There are two main reasons or motives for keeping reserves in an economy. These are the precautionary and non-precautionary motives or needs. Assessing Reserve Adequacy—Specific Proposals (2014) proffers that the non-precautionary motives of reserves are driven by policies to allocate revenues emanating from natural resources to future generations. These allocations explain why resource-rich countries are likely to hold more reserves. However, the precautionary motives of holding reserves are for intervention purposes and need to be prepared for emergencies and shocks in the economy. The intervention need for reserves is the primary focus of many research works on reserve holdings (Roger, 1993; Steiner, 2013; Pina, 2015; Ghana, 2017; Hongxing and Rahaman, 2018). In a survey of Assessing Reserve Adequacy—Specific Proposals (2014), about three-quarters of respondent country authorities viewed precautionary liquidity needs as the critical reason to hold reserves. This precautionary motive of holding reserves must address the following three logics:

- Provision of ready liquidity in times of shocks,
- Ability to sustain current growth figures and to propel the economy to future higher growth paths, and
- Reasonability in terms of costs of holding excess reserves that are not needed.

A good reserve model is, therefore, one that possesses all of these attributes. This feature, therefore, becomes the strength of the OPREM model we are proposing in this research.

Several factors influence the evolution and building of foreign exchange reserves. These factors are summarized as the changing patterns of international trade, institutional changes in the economy, and structural shifts in production (Oyeniran and Alamu, 2020). Also, fear of capital mobility by some central banks may influence the composition and amount of foreign exchange. Steiner (2013) asserts that many central banks use reserves as substitutes for capital control measures.

The effects of each of the factors on an economy are mixed. These mixed effects are partly because of the motive of keeping reserves and the extent of financial sector development and integration.

**Figure 1:**
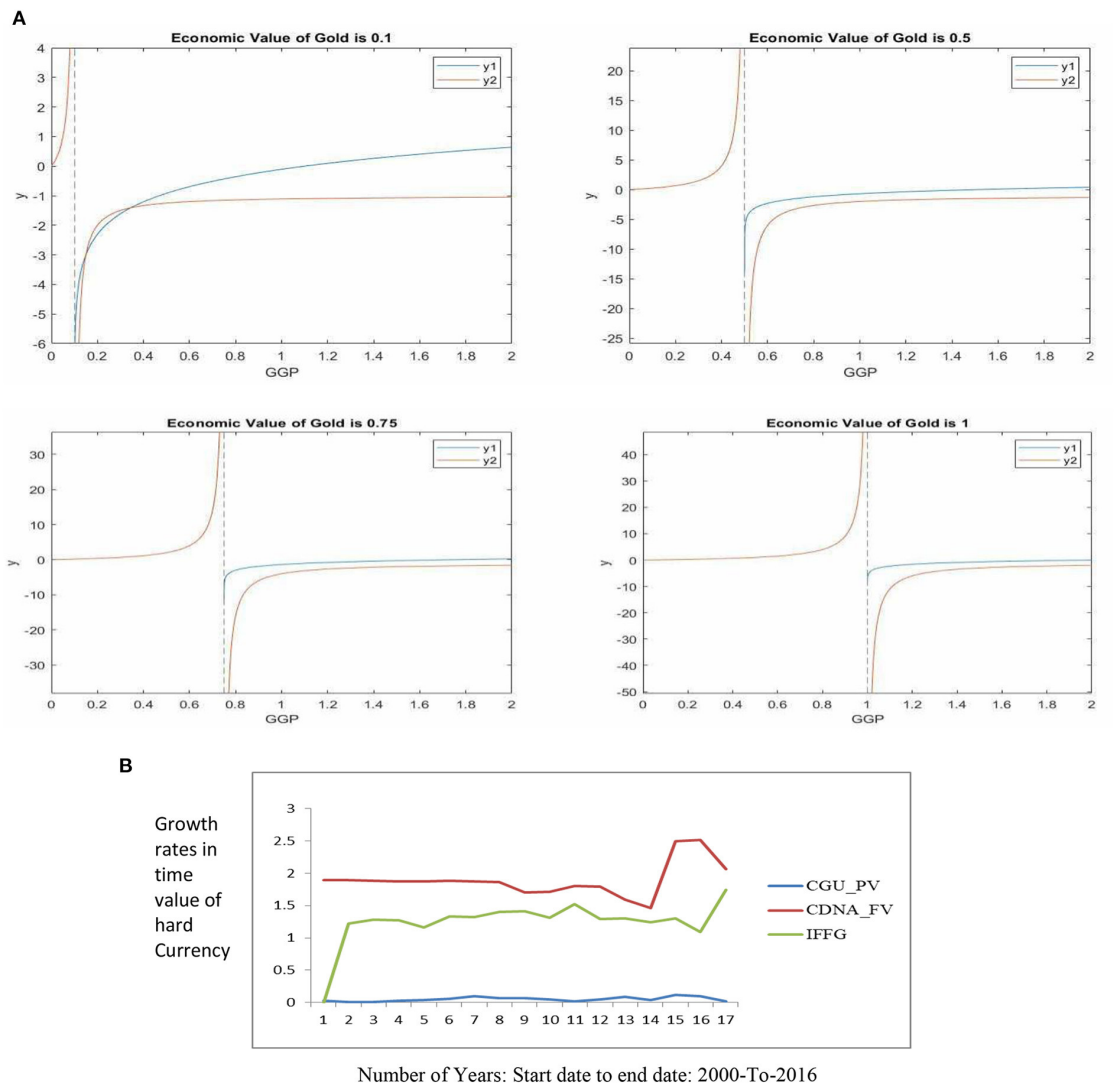
**(A)** Critical Points Graph of the IFFG of the OPREM model. **(B)** Plot of the OPREM Component series (Reserve component analysis).

Furthermore, the production layout in a country equally has a great impact on reserves. For instance, a commodity-intensive economy may hold more reserves than a country that is not a commodity-intensive economy (Assessing Reserve Adequacy—Specific Proposals, 2014), working on international reserve growth for 24 developed economies and 154 developing economies between 1970 and 2009, observed that whiles the reserves of developed nations are shrinking, that of their developing counterparts are increasing. Figure 1B presents these differences in reserves.

### Empirical literature

Research on assessing a country's optimal reserves has become a relevant macro-financial indicator that the IMF and other world financial institutions have always sort to capture in their reports on countries' resilience to external shocks and exposures. These reports rely heavily on the research conclusions of Kindleberger (1961), Heller (1966), Ben-Bassat and Gottlieb (1992), Greenspan (1999), and Wijnholds and Kapteyn (2001), who provide benchmarks against economic variables and a periodic assessment of the cost-benefits of reserves. Though these previous works have contributed significantly to building the literature on reserves, procyclicality in reserve build-up of developing and emerging countries has raised the cost of reserve stocks in these countries. These have left several questions on the cost-effectiveness of benchmark models such as the imports model, reserve-to-broad money, and the Greenspan or reserve-to-short-term debts model. These doubts over the previous models have led Ghana (2017) to refer to these benchmarks as producing imprecise estimates of reserve stocks. Also, nonrealistic assumptions in some models, such as the zero reserve depletion assumption in Ben-Bassat and Gottlieb (1992), have dragged the optimal reserve level upwards and contributed to the increasing cost of holding reserves.

Heller (1966) was the pioneer in analyzing and formulating optimal reserves using cost-benefit analysis. This research was followed later by several works expounding on a similar concept (see Hamada and Ueda, 1977; Frenkel and Jovanovic, 1981; Ben-Bassat and Gottlieb, 1992; Tule et al., 2015). Most of these research works are similar in how they view and treat the reserve stock and the cost of default in meeting the external obligations of borrowing countries. These researches are grouped into two broad classes according to the approach and method of the research. The first group of researchers is those we have identified as following the approach of Heller (1966), henceforth known as the H-Models. The second group shares a similar approach with Ben-Bassat and Gottlieb (1992), henceforth known as the B-G Models. The B-G models estimate the potential cost of default (π*C*_0_) as a proxy for the probable cost of reserve depletion (π), this likelihood (π) is the probability that a country may become insolvent and has to face the consequences of not honoring its debts obligations to external borrowers. While the B-G models derive these probability assumptions from a cross-sectional analysis of borrowing countries that had defaulted from their external debt obligations and have had a renegotiation of their debts during the sample period, the H-Models derive their depletion probabilities (π) from a symmetric random walk process by viewing the probable cost of default as the foregone GDP (π*C*_0_). A simple random walk is symmetric if observations have a constant probability over time (Gujarati, 2004). Heller (1966) asserts that the probability function, from his random walk process, does not change with the level of reserves. This assumption breeds subjectivity in the methodologies of the H-models (Clark, 1970, and Ben-Bassat and Gottlieb, 1992). Also, both groups of reserve models view the cost of reserve depletion as the consequence of a country running out of reserves. However, the H-models narrow this scope down to the loss in GDP.

The research methodologies of the H and B-G Models do not collectively exhaust the logic of holding reserves. For example, the focus of B-G Models is on liquidity and cost minimization. It fails to factor in the sustainability of the current growth path and future growth projections. The H-Models only focused on sustaining current growth paths and future growth projections and cost minimization. It fails to consider the need for liquidity in the analysis. Table 1A shows the models for optimal reserve holdings and the logic or aims considered in previous research models' methodologies.

**Table 1A:** Models for optimal reserves and their Logics.

**MODEL**	**Purpose and logic classification of reserves**		
	**Liquidity**	**Sustainability** **and Projections**	**Reasonability**
H-Models	Unmeasured	✓✓	✓✓
B-G Models	✓✓	Unmeasured	✓✓
Our Model (OPREM)	✓✓	✓✓	✓✓

A desirable optimal reserve model should consider all the logic or aims of holding reserves like the OPREM model in this research.

What is common to these different methodologies is the incorporation of cost minimization technics. However, the cost minimization procedure methodologies differ significantly between these two broad models, as we have already explained. The methodology adopted in this paper minimizes the total cost of holding reserves by treating each component of the reserve stock as a separate fund or wealth which are exogenously related to one another in the reserve build-up process. We also treat the reserves fund as a multi-period account with perpetuity instead of the *ad hoc* treatment given to the fund by the H and B-G models. However, this paper also treats non-optimal reserves' cost as the variations in economic output along with the H-Models. The significance of this methodology is in highlighting the exogenous components of the reserve stock and emphasizing the continuity of the reserve stock.

Prabheesh (2013) determined India's optimal reserve level and indicated that the actual reserves are higher than the optimal reserves across the sample period 1994–2008, except for only the 1997–1998 period. Wijnholds and Kapteyn (2001) found that countries on a managed float or fixed exchange rate regime could maintain reserves to cover around ten to twenty per cent of broad money while the IMF suggests 3 months of import cover.

### Ghana's economic outlook post COVID-19

Ghana is an economy that runs a managed currency regime. This system encourages the holding of foreign exchange reserves by the Central Bank for intervention into the currency market to ensure stability. Also, foreign exchange reserve is a useful tool for intervention against shocks and external vulnerabilities, and the quantum of reserves can be a good determinant of countries' resilience. The adequacy of Ghana's reserve stock for intervention purposes has been consistently questioned by researchers such as Hongxing and Rahaman (2018), Abdul-Rashid and Yao (2019) and the Ghana: 2021 Article IV Consultation-Press Release; Staff Report; and Statement by the Executive Director for Ghana (2021). This issue is recently revisited with great vigor when COVID-19 hit and exposed the vulnerabilities of the economy. The government response to the COVID-19 pandemic helped to contain the pandemic (Ghana: 2021 Article IV Consultation-Press Release; Staff Report; and Statement by the Executive Director for Ghana, 2021), but this was achieved at the expense of a record high debt or borrowing threshold, a growth rate as low as 0.4% in 2020, a record high inflation, and a deteriorating exchange rate, among others. Recently, Ghana's government debt instruments are classified as junk by major rating agencies such as S&P, Fitch, and Moody. These bad ratings have increased borrowing costs to government, hence, forcing government to re-focus attention to the domestic market to finance its activities. The directors of IMF asserted regarding the banking and financial sector in Ghana:

“*… the financial sector cleanup had made the sector more resilient but stressed that banks' growing holdings of sovereign debt creates risks and crowds out private sector credit”*

This recent outlook could have probably been different if the appropriate foreign exchange reserves for intervention were held by the Central Bank.

## Theoretical model and hypothesis development

This section discusses the theoretical model of the proposed research and the development of the research hypotheses.

### Theoretical model

This research uses both the inductive and deductive research approach to propose an Optimal Reserve model using the principle of Least Square residual minimization of Hard currencies and gold in the reserves stock of central banks.

We note that the amount and composition of the reserve stock of countries are different and that countries also have a financial obligation to optimize their holdings of these reserves. This research assumes that the best way to optimize a central bank's reserve holdings is to treat reserves as having an identifiable exogenous or separate component that can be maximized independently of one another *(Assumption 1)*. These identifiable components include foreign currencies in the reserves of central banks, foreign bank deposits, foreign treasury bills, short-term and long-term foreign government securities, gold reserves, special drawing rights, and international monetary fund reserve positions.

We note that these assets are independent of one another and contribute differently to the reserve stock. The International Monetary Fund (2013) and Assessing Reserve Adequacy—Specific Proposals (2014) have classified these assets into liquid and less liquid securities and have advised against using less liquid securities in any research on reserve holdings for intervention. In line with the advice given in International Monetary Fund (2013) and the Assessing Reserve Adequacy—Specific Proposals (2014), this research has eliminated less liquid securities *(excluding gold)* in the analysis of the reserve stock of central banks.

The research also negates the complete depletion of reserves and emphasizes the carrying over from one period to another *(Assumption 2)*. These pre-supposes that the reserve stock is a continuous or a perpetual fund that is reimbursed periodically. Proper management of this fund demands that inflows into the fund must be clearly defined and sources earmarked. This reimbursement is to ensure a perpetual building of the fund based on some acceptance criteria.

We further assume that the constituents or components of reserves are not equally represented in reserve holdings. We have therefore assigned weights of φ and ω to hard currencies and gold, respectively, in the reserves of central banks. Where we defined φ as the percentage of reserves that are in hard currencies, and ω as the percentage of reserves in gold. The research assumes a relationship between φ and ω and holds that the expression:

*φ* > *ω*,

is true for most developing and emerging countries today *(Assumption 3)*.

This relationship is assumed because of the reduced role of gold and the increased role of hard currencies in reserve management for intervention purposes. The collapse of the fixed exchange rate system and the passage of the standard gold era support this theory. These have drastically reduced the significance of gold in central banks' precautionary reserves. For example, gold reserves in the case study country remain constant at 8.74 metric tons for more than a decade (Trading Economics, 2019).

Following these assumptions, the research has modeled the holding cost of reserves as:

The opportunity cost of holding Hard or foreign currencies (HCV)


$$\begin{array}{l} \text{plus }\left(+\;\right) \end{array}$$

The opportunity cost of holding gold in the reserves is in equation (1).


(1) $$\begin{array}{l} \text{Cost}=\text{HT}{\text{C}}^{\varphi }+\text{IFF}{\text{G}}^{\omega } \end{array}$$

Where

**HTC** is the Total cost (opportunity cost) of hard currencies.
**IFFG** is the impact factor of gold or the opportunity cost of holding gold in the reserves.

By limiting reserve stock to only hard currencies and gold, we restrict the reserve stock to only two components. This approach brings to the fore other implicit assumptions such as a risk-free substitution between the Special Drawing Rights (SDRs) and hard currencies. This assumption is because of the similarities between hard currencies and SDRs in reserves of central banks. The elimination of Government investment in foreign assets and securities from the stock of reserves is because they are not readily available to assist or support interventions (i.e., illiquid). This method is in adherence to the International Monetary Fund (2013) and the Assessing Reserve Adequacy—Specific Proposals (2014).

The Total Cost of Hard Currencies (HTC), stated in equation (1), is expressed as:

Hard currency appreciation not taken advantage of now (CGU) plus (+) Hard currency depreciation in the future not avoided now (CDNA).

This definition, mathematically, is in equation (2).


(2) $$\begin{array}{l} \text{HTC}={\left(\text{CGU }+\text{ CDNA }\right)}^{\varphi }\\ HTC=CGU^{\varphi \left(1-g\left(\text{dinf}-\text{r}\right)\right)}+CDNA^{g\left(\text{dinf}-\text{r}\right)} \end{array}$$

Empirical research works have shown that the amount, fraction or percentage of hard currencies in reserves (φ), and its associated costs depend on the nature of the intervention hard currencies are used for (i.e., CGU or CDNA). For example, many research works associate large stocks of international reserves with undervalued exchange rates and currency depreciations (Aizenman and Sun, 2012; Dominguez et al., 2012; Pina, 2015). Therefore, the φ value, adjusted for the variance between the growths in differential inflation and differential interest rates, are used as weights for CGU and CDNA. Equal weight for CGU and CDNA means the absence of currency manipulations, and an unequal weight means the presence of currency manipulation by monetary authorities.

Therefore, the Total cost function in equation (1) is re-written in equation (3).


(3) $$\begin{array}{l} Cost=(CGU^{\varphi (1-g\left(\text{dinf}-\text{r}\right))}+CDNA^{\varphi (g\left(\text{dinf}-\text{r}\right))})+IFFG \end{array}$$

Where


(4) $$\begin{array}{l} CGU={\left(HPV_{i}-\frac{\sum_{i=1}^{n}HPV_{i}}{n}\right)}^{2} \end{array}$$

(5) $$\begin{array}{l} CDNA={\left(HFV_{i}-\frac{\sum_{i=1}^{n}HFV_{i}}{n}\right)}^{2} \end{array}$$

Where

$\frac{\overset{n}{\underset{i=1}{\sum }}HPV_{i}}{n}$ is the mean of the Present Value of Hard Currency, i.e., *HPV*_μ_
$\frac{\overset{n}{\underset{i=1}{\sum }}HFV_{i}}{n}$ is the mean of the Future Value of Hard Currency, i.e., *HPV*_μ_.

We note that the variables *HPV* and *HFV* in equation (4) and equation (5) are the present value of hard currencies and the future value of hard currencies, respectively. Also, *i* in the equations represents the product of the period (*t*) and data frequency (*j*).

Note that the research placed some restrictions on the values of (*j*) in the model where we assume (*j*) to be constant within an analysis period (*t*). These values carry the same number as the year of analysis *(Assumption 4)*. For example, all data frequencies or intervals for the first year take the number *1*. For the second year, all data frequencies or intervals take the number *2*, and so on. We mean that economic variables or agents take at least a year to respond to market information by this assumption. *Assumption 4* was to control the effect of high-frequency data on the estimates of the time values. This assumption is consistent with conventional economics and theory because most economic variables in literature will only react to policy or market information with some lag(s). Therefore, economic variables or agents may not experience much change within a year ***t*** with ***j*** frequencies (Gujarati, 2004).

Therefore, the Total Cost function with gold and hard currencies in the reserve stock is re-stated in equation (6).


(6) $$\begin{array}{l} Cost=({\left(HPV_{i}-\frac{\sum_{i=1}^{n}HPV_{i}}{n}\right)}^{2\varphi (1-g\left(\text{dinf}-\text{r}\right))}\\ \;+{\left(HPV_{i}-\frac{\sum_{i=1}^{n}HPV_{i}}{n}\right)}^{2\varphi (g\left(\text{dinf}-\text{r}\right))})+IFFG^{\omega } \end{array}$$

On the Impact Factor of Gold (IFFG), this research defines the effect of gold in the reserve stock of the central bank as the benefits lost on holding gold in the reserves. This is expressed as:

Global prices of gold in domestic currency – Real Economic value of gold.

The Real Economic value of gold is defined in this research as the Domestic currency in Circulation support ratings per gold in reserves. Economic or financial theory may define this as the opportunity cost of holding gold (a return that investors could achieve by purchasing or investing in other assets such as stock, bonds, or some hard currencies). As used in this research, the economic value of gold is therefore not estimating the opportunity cost of gold as in conventional theories. It is, rather, a measure of the extent to which gold in reserves can be optimized in other to strengthen or weaken domestic currencies relative to other currencies. This understanding of the relevance of gold in reserves is more crucial to countries with fixed exchange rate regimes. Therefore, fluctuations in the real economic value of gold will mediate the impact gold has on the economy (i.e., the IFFG). The measure of the economic value of gold (i.e., $\frac{DCIC_{i}}{G_{i}}$) is used to weigh the risk of default on holders of a Nation's currency. Note that the B-G models estimate this potential cost of default (π*C*_0_) as a proxy for the probable cost of reserve depletion, whiles the H-models view the cost of default as the foregone output (i.e., GDP (π*C*_0_)) derived from the asymmetric random walk process.

Note that the decision to use currency in circulation (CIC) in the computation of the economic value of gold and not broad money (M2) is because the former is money created by a central bank which gold in reserves are used to support. This is not the case with M2, which include monetary instruments created by commercial banks on a fractional system basis. The IFFG is expressed in equation (7).


(7) $$\begin{array}{l} IFFG=GGP_{d,i}-\frac{DCIC_{i}}{G_{i}} \end{array}$$

Where

*GGP*_*d*_ is the global gold price in domestic currency, also written as GGP.
*DCIC* is Domestic currency in circulation.
*G* is the number of gold in reserves (measured in an ounce).

The research objective is to minimize the cost function in equation (7). We, therefore, state the minimization function as:


(8) $$\begin{array}{l} Min IFFG={\left(GGP_{d,i}-\frac{DCIC_{i}}{G_{i}}\right)}^{SM} \end{array}$$

Where

*SM* is the ‘stability monitor or indicator’ and expressed as:


(9) $$\begin{array}{l} SM =\frac{\%\Delta GGP}{\%\Delta HCV} \end{array}$$

*SM* addresses the trade-off between hard currencies in reserves and gold in reserves (i.e., assuming gold were to be exchanged for some hard currencies). Each of these elements in the composition of the central bank reserves has a unique benefit. For example, experience has shown that hard currencies are relatively stable, especially when protecting against the downside (a decline), while gold prices are highly volatile. However, gold turns to appreciate faster. Therefore, the SM index helps us incorporate past, current, and future trade-off *growth expectations* between the amount of gold and hard currencies in the IFFG. In short, the SM index in equation (10) plugs in *growth expectations* and opportunity cost in the IFFG. The SM index has implications for managers in the management of the reserve stock. It indicates when to consider gold in reserves and when not to consider gold in reserve management. This condition is simply dependent on the value of the SM index (i.e., When *SM* = 1, freeze gold out. Otherwise, unfreeze gold in the equation of optimal reserves).

To plug in the actual time it takes to realize the growth expectations of reserves, we take the log of the IFFG.


(10) $$\begin{array}{l} \text{IFFG}=\;|SM|\;\mathrm{LOG}{\left(GGP_{d,i}-\frac{DCIC_{i}}{G_{i}}\right)}^{2\left(\omega \right)} \end{array}$$

Where:

ω is the weight of gold in the reserves of a central bank, and ′2′ is a constant in the formula to avoid a negative IFFG.

Therefore, the Total Cost of holding reserves is expressed as:


(11) $$\begin{array}{l} Cost=({\left(HPV_{i}-\frac{\sum_{i=1}^{n}HPV_{i}}{n}\right)}^{2\;\varphi (1-g\left(\text{dinf}-\text{r}\right))}\\ \;+{\left(HFV_{i}-\frac{\sum_{i=1}^{n}HFV_{i}}{n}\right)}^{2\varphi (g\left(\text{dinf}-\text{r}\right))})\\ \;+|SM|\mathrm{LOG}{\left(GGP_{d,i}-\frac{DCIC_{i}}{G_{i}}\right)}^{2(\omega )} \end{array}$$

Having estimated the Total opportunity cost of holding reserves, we multiply the cost by a country's total inflows. This inflow is a measure of a country's exposure to international transactions and trade. We measure this exposure as the simple average sum of a country's Total Imports, Foreign Direct Investment, and Total External Debts.

Many researchers and models have used either one of these variables to measure exposure to external influence and shocks, especially the imports. The procedure of using the average of more than one variable in this research is to avoid sticking to inelastic variables, such as imports, which do not often allow for easy adjustments to economic trends in developing countries.

The final drove model, i.e., the Optimal Reserve Model (OPREM), is stated as:


(12) $$\begin{array}{l} OPREM_{\left(i\right)}=\{({\left(HPV_{i}-\frac{\sum_{i=1}^{n}HPV_{i}}{n}\right)}^{2\varphi (1-g\left(\text{dinf}-\text{r}\right))}\\ \;+{\left(HFV_{i}-\frac{\sum_{i=1}^{n}HFV_{i}}{n}\right)}^{2\varphi (g\left(\text{dinf}-\text{r}\right))})\\ \;+\left[|SM|\mathrm{LOG}{\left(GGP_{d,i}-\frac{DCIC_{i}}{G_{i}}\right)}^{2(\omega )}\right]\}\\ \;\times \;INFLOWS_{\left(i\right)} \end{array}$$

Where

‘*INFLOWS'* is the average sum of a country's Imports, Foreign Direct Investment, and Total External Debts.

The reserve model in equation (12) addresses shortfalls in previous reserve models by addressing the three main logics of holding reserves already outlined in the introductory section.

Firstly, the reserve model proposed in this research increases the reserve stock's adaptability to adjust to economic trends and activities. These auto-adjustments increase reserves' effectiveness as a monetary policy tool, and again, as a protection against external economic fluctuations most prevalent in developing and emerging countries.

Secondly, the proposed model sets reserve stocks that are highly liquid and will be readily available if a shock hits the economy. We achieved this feat by withdrawing less liquid securities such as government investments in bonds and equities out of the reserve stock. Also, the total cost function, which is also a measure of the potential risk of holding reserves, is multiplied by the average total inflows, not just a single sectoral variable like M2, short-term debts, imports or exports. This approach protects the OPREM model from the inelastic behavior some of these variables exhibit. This behavior mainly results in imprecise estimates of the reserve stocks (Ghana, 2017). Besides, many researchers and models have used either of these variables to measure exposure to external influence and shocks, especially the imports.

Thirdly, the assumption of no zero depletion of reserves and the reserves stock's perpetuity ensures that the current stock of reserves is always sufficient to sustain current and future economic growth projections. Also, the research model satisfies the last logic of holding reserves (i.e., to reduce the total cost of holding reserves) by minimizing the Least Square Residuals of the cost of each component of the reserve stock over time. This methodology uses a component-based approach to minimizing the total cost of reserves. Many research works have approached this based on probabilities and perceptions developed by market agents on the default risk. Ben-Bassat and Gottlieb (1992) assert that this approach introduces subjectivity in previous research models.

### Partial derivative of the OPREM model

This section begins from Equation (12).


(13) $$\begin{array}{l} OPREM_{\left(i\right)}=\{{\left(HPV_{i}-\frac{\sum_{i=1}^{n}HPV_{i}}{n}\right)}^{2\varphi \left(1-g\left(\dim f-r\right)\right)}\\ \;+{\left(HFV_{i}-\frac{\sum_{i=1}^{n}HPV_{\left(i\right)}}{n}\right)}^{2\varphi \left(g\left(dinf-r\right)\right)}\\ \;+\left(\left|SM\right|Log{\left(GGP_{d,i}-\frac{DCIC_{i}}{G_{i}}\right)}^{2\omega }\right)\}\\ \;\cdot \;INFLOWS_{\left(i\right)}\\ \;OPREM=\{{\left(HPV-HPV_{\mu }\right)}^{2\varphi \left(1-g\left(dinf-r\right)\right)}\\ \;+{\left(HFV-HFV_{\mu }\right)}^{2\varphi \left(g\left(dinf-r\right)\right)}\\ \;+\left(\left|SM\right|Log{\left(GGP-\frac{DCIC}{G}\right)}^{2\omega }\right)\}\\ \;\times \;INFLOWS \end{array}$$

We now take partial derivatives of OPREM with respect to these three variables:

i. HPV,
ii. HFV, and
iii. GGP.

#### Derivative of OPREM with respect to HPV


$$\begin{array}{ll} \frac{\partial \left(OPREM\right)}{\partial HPV}=2\varphi \left(1-g\left(dinf-r\right)\right)\\ {\left(HPV-HPV_{\mu }\right)}^{2\varphi \left(1-g\left(dinf-r\right)\right)-1}\\ \times & INFLOWS.\left(1-\frac{1}{n}\right).\\ \text{Setting }\frac{\partial \left(OPREM\right)}{\partial HPV}=0,\text{we have}\\ 2 & \varphi \left(1-g\left(dinf-r\right)\right)\\ {\left(HPV-HPV_{\mu }\right)}^{2\varphi \left(1-g\left(dinf-r\right)\right)-1} \end{array}$$

(14) $$\begin{array}{ll} \times & INFLOWS.\left(1-\frac{1}{n}\right)=0 \end{array}$$

The variable; INFLOWS, is a number which value cannot be zero (0) so long as a country operates an open economy. Therefore, *INFLOWS* ≠ 0 ***(Condition 1)***. Also, the proportion of Hard Currencies in a country's reserve, i.e., φ, cannot be zero (φ ≠ 0) ***(Condition 2)***. This makes sense as a country's Central Bank will always have some holdings of foreign currencies.

To calculate for the value of HPV, we set the function 2φ(1 − *g*(*dinf* − *r*)) to be greater than zero ***(Condition 3)***.


(15) $$\begin{array}{l} 2\varphi \left(1-g\left(dinf-r\right)\right)\neq 0 \end{array}$$

This is because,


(16) $$\begin{array}{l} g\left(dinf-r\right)\neq 1 \end{array}$$

For (*dinf* − *r*) = 1 to be true, the Central Bank must not take any action when inflation is changed by 100%. This scenario is highly unlikely especially in an inflation-targeting economy where interest rate is the main monetary policy tool and is also not likely to be zero (0). Therefore, the condition in equation (16), i.e., ***(Condition 4)***, and equation (15) are true for real world situations.

Given the fact that condition 1, 2, and 3 exist, the Equation (14) which is:


$$\begin{array}{l} 2\varphi \left(1-g\left(dinf-r\right)\right){\left(HPV-HPV_{\mu }\right)}^{2\varphi \left(1-g\left(dinf-r\right)\right)-1}\\ \times INFLOWS.\left(1-\frac{1}{n}\right)=\;0, \end{array}$$

Can only be zero (0) if;


$$\begin{array}{l} {\left(HPV-HPV_{\mu }\right)}^{2\varphi \left(1-g\left(dinf-r\right)\right)-1}=0,\text{ Or} \end{array}$$

(17) $$\begin{array}{l} HPV-HPV_{\mu }=0 \end{array}$$

Note that equation (17) is only true on the condition that:


$$\begin{array}{l} HPV=HPV_{\mu }\;\left(\text{Condition }5\right) \end{array}$$

This condition simply implies that the present value of hard currency, *HPV*, must be equal to the average of present value of hard currency *HPV*_μ_. This means the spread or dispersion is zero. Condition (5) can, therefore, only exist in an environment of currency or exchange rate stability. Therefore, for Equation (14) or the effect of the HPV to be zero, an economy should have a stable currency or exchange rate. From this analysis, the effect of the HPV on reserve stock accumulation on developed nations will be relatively small compared to their developing counterparts.

#### Derivative of OPREM with respect to HFV


(18) $$\begin{array}{l} \frac{\partial \left(OPREM\right)}{\partial HFV}=2\varphi \left(g\left(dinf-r\right)\right)\\ {\left(HFV-HFV_{\mu }\right)}^{2\varphi \left(g\left(dinf-r\right)\right)-1}.\;INFLOWS.\;\left(1-\frac{1}{n}\right) \end{array}$$

Setting $\frac{\partial \left(OPREM\right)}{\partial HFV}=\;0$,

we have;


(19) $$\begin{array}{l} 2\varphi \left(g\left(dinf-r\right)\right){\left(HFV-HFV_{\mu }\right)}^{2\varphi \left(g\left(dinf-r\right)\right)-1}\\ \times INFLOWS.\left(1-\frac{1}{n}\right)=0 \end{array}$$

But since φ, and 2φ(*g*(*dinf* − *r*)) are already defined under condition 1 and 3 as not equal to zero (0), i.e.:

φ ≠ 0, and
(*g*(*dinf* − *r*)) ≠ 0

The only condition under which Equation (19) will be zero (0) is:


(20) $$\begin{array}{l} HFV-HFV_{\mu }=0 \end{array}$$

Or;


$$\begin{array}{l} HFV=HFV_{\mu }\; \end{array}$$

Therefore, for the effect of the HFV to be zero, there should be zero spread between *HFV*, and *HFV*_μ_. The only instance under which this is also possible is when participants in the foreign exchange market belief the currency or exchange rate will remain unchanged in the future. Under this condition, the effect of the HFV on reserve stock accumulation is zero.

#### Derivative of OPREM with respect to GGP

For the $\frac{\partial \left(OPREM\right)}{\partial GGP}$,

We let,


(21) $$\begin{array}{l} y=|SM|Log{\left(GGP-\frac{DCIC}{G}\right)}^{2\omega } \end{array}$$

Note that;


(22) $$\begin{array}{l} |SM|=\frac{GGP}{HCV} \end{array}$$

Therefore, substituting Equation (22) into Equation (21), we have:


(23) $$\begin{array}{l} y=|\frac{GGP}{HCV}|Log{\left(GGP-\frac{DCIC}{G}\right)}^{2\omega } \end{array}$$

(24) $$\begin{array}{l} y=Log{\left(GGP-\frac{DCIC}{G}\right)}^{2\omega \frac{GGP}{HCV}} \end{array}$$

(25) $$\begin{array}{l} 10^{y}={\left(GGP-\frac{DCIC}{G}\right)}^{2\omega \frac{GGP}{HCV}} \end{array}$$

(26) $$\begin{array}{l} \ln\;10^{y}={\ln\left(GGP-\frac{DCIC}{G}\right)}^{2\omega \frac{GGP}{HCV}} \end{array}$$

(27) $$\begin{array}{l} y\ln\;10\;=\;2\omega \frac{GGP}{HCV}\ln\left(GGP-\frac{DCIC}{G}\right) \end{array}$$

(28) $$\begin{array}{l} \frac{\partial y}{\partial GGP}=\frac{2\omega }{HCV}\ln\left(GGP-\frac{DCIC}{G}\right)+\frac{2\omega GGP}{HCV}\frac{1}{GGP-\frac{DCIC}{G}} \end{array}$$

Setting $\frac{\partial y}{\partial GGP}=0$, we have


$$\begin{array}{l} \frac{2\omega }{HCV}\left(\ln\left(GGP-\frac{DCIC}{G}\right)+\frac{GGP}{GGP-\frac{DCIC}{G}}\right)=0, \end{array}$$

(29) $$\begin{array}{l} \text{Since }\omega \neq 0,\text{we have} \end{array}$$

(30) $$\begin{array}{l} \ln\left(GGP-\frac{DCIC}{G}\right)+\frac{GGP}{GGP-\frac{DCIC}{G}}=0 \end{array}$$

(31) $$\begin{array}{l} \ln\left(GGP-\frac{DCIC}{G}\right)=\frac{GGP}{\frac{DCIC}{G}-GGP} \end{array}$$

Now, we will have to find a GGP value that satisfies the equation above, and will minimize the OPREM. Since Equation (31) involves two functions, we resort to solving this problem using graph plots in MATLAB where we represent *GGP* by *x*, and $\frac{DCIC}{G}$ by *k*. However, we note here that *k* ≤ 1 (Condition 6). This is because every currency in the economy must be support by gold (i.e., the Gold Standard System). Table … therefore computes the minimum impact of IFFG on reserve accumulation by taking $\frac{DCIC}{G}$ or *k* values of 0.1–1. The following equations are written to solve these functions in MATLAB:

*syms x*
*y*_1_ = log(*x* − *k*);

$y_{2}=\frac{x.}{\left(k-x\right)};\;$

*fplot*(*y*_1_, [0 2]);*hold on*; *fplot*(*y*_2_, [0 2]);
*xlabel*(″*GGP*″)
*ylabel*(″*y*″)
*title*(′*Economic value of Gold is k*′)
*legend* $\left(\prime y_{1}^{\prime }{,}^{\prime y_{2}^{\prime }}\right)$
*x*_*int* = *solve*(*y*_1_ = =*y*_2_, 0)
*x*_*int* = *eval*(*x*_*int*_)
*y*_*int* = *eval*(*subs*(*y*_2_, *x*_*int*))

The output graphs of the computations are shown in Figure 1A.

Table 1B shows the values of $\frac{DCIC}{G}$, and *GGP*. When these values are put back into the function of IFFG, we will get the impact gold has on reserve stock accumulation. Table 1C calculates the OPREM values given that the effect of hard currencies is zero (i.e., *HPV* − *HPV*_μ_ = 0 and *HFV* − *HFV*_μ_ = 0 which means stable currency or zero standard deviation of the present and future values of hard currencies). Exchange rates are stable when there is zero dispersion or standard deviation of the present and future value of hard currency. Under this circumstance, the effects of hard currencies in the reserve stock build-up for precautionary purpose will be zero regardless of prevailing interest rates and inflationary figures. This will cause the minimum and maximum reserves to accumulate or hold to be a product of the minimum and maximum critical values of the gold impact factor and the “inflows” into an economy, respectively. For instance, in Table 1C, at the $\frac{DCIC}{G}$ value of 0.1, the graphical approach for solving simultaneous equations in MATLAB found two critical values (i.e., the Minimum value and the Maximum value). At these values, the optimal reserves, when exchange rate is stable, should be within the range of GHc130,994,530.58 – GHc676,221,501.47 of the amount of gold (see Table 1C). The major determinants of these amounts are the Stability Monitor (*SM*) which is the percentage trade-off between global gold prices and hard currencies, and also the domestic currency in circulation in the economy. The optimal reserve values when this condition is not held are attached as a Supplementary Document.

**Table 1B:** Critical Value points for the IFFG function.

** $\frac{DCIC}{G}$ **	***GGP***
0.10	0.3443 (max value: 0.1493)
0.15	0.2805 + 0.0632i
0.20	0.3142 + 0.1339i
0.25	0.3483 + 0.1798i
0.30	0.3827 + 0.2171i
0.35	0.4174 + 0.2496i
0.40	0.4523 + 0.2789i
0.45	0.4874 + 0.3059i
0.50	0.5226 +0.3312i
0.55	0.5581 + 0.3551i
0.60	0.5936 + 0.3778i
0.65	0.6293 + 0.3996i
0.70	0.6652 + 0.4205i
0.75	0.7011 + 0.4406i
0.80	0.7371 + 0.4602i
0.85	0.7733 + 0.4792i
0.90	0.8095 + 0.4976i
0.95	0.8458 + 0.5156i
1.00	0.8822 + 0.53321i

**Table 1C:** Optimal Reserves for different critical values given exchange rate stability.

**DCIC/G**		**GGP**	**HCV**	***%ΔGGP***	***%ΔHCV***	** $SM=\frac{\%\Delta GGP}{\%\Delta HCV}$ **	**|*SM*|**	$\left(GGP_{d,i}-\frac{DCIC_{i}}{G_{i}}\right)$		**Min IFFG**	**Absolute values** **of Min IFFG**	**Inflows in Ghc**	**OPREM**
0.1000	Min	0.3443	0.7768	0.3443	0.7768	0.4432	0.4432	0.7544	−0.1224	−0.0543	0.0543	2,413,646,832.94	130,994,530.58
	Max	0.1493		0.1493		0.1922	0.1922	0.6836	−0.1652	0.2614	0.2614	2,586,619,393.77	676,221,501.47
0.1500		0.2805	0.8095	−0.1853	0.0420	−4.4111	4.4111	0.6655	−0.1769	−0.7802	0.7802	3,137,905,085.91	2,448,293,289.54
0.2000		0.3142	1.0012	0.1201	0.2369	0.5072	0.5072	0.6479	−0.1885	−0.0956	0.0956	3,766,899,606.27	360,095,120.64
0.2500		0.3483	1.1560	0.1085	0.1546	0.7018	0.7018	0.6288	−0.2015	−0.1414	0.1414	3,919,539,145.25	554,231,955.24
0.3000		0.3827	1.2860	0.0988	0.1125	0.8783	0.8783	0.6074	−0.2165	−0.1901	0.1901	4,351,527,130.70	827,426,371.48
0.3500		0.4174	1.1928	0.0907	−0.0725	−1.2507	1.2507	0.5831	−0.2343	−0.2930	0.2930	3,946,229,034.60	1,156,238,222.96
0.4000		0.4523	1.3184	0.0836	0.1053	0.7941	0.7941	0.5542	−0.2563	−0.2035	0.2035	5,463,145,699.62	1,111,838,709.40
0.4500		0.4874	1.4482	0.0776	0.0985	0.7880	0.7880	0.5183	−0.2854	−0.2249	0.2249	8,341,889,132.90	1,876,285,719.33
0.5000		0.5226	1.5487	0.0722	0.0693	1.0414	1.0414	0.4686	−0.3292	−0.3428	0.3428	10,227,967,193.50	3,506,213,368.91
0.5500		0.5581	1.9482	0.0679	0.2580	0.2633	0.2633	0.3817	−0.4183	−0.1101	0.1101	12,680,187,217.20	1,396,510,768.55
0.6000		0.5936	1.8729	0.0636	−0.0387	−1.6443	1.6443	0.3641	−0.4388	−0.7215	0.7215	18,173,469,508.64	13,111,332,509.74
0.6500		0.6293	2.0615	0.0601	0.1007	0.5970	0.5970	0.4605	−0.3368	−0.2011	0.2011	25,241,909,036.74	5,075,606,388.78
0.7000		0.6652	2.4627	0.0570	0.1946	0.2931	0.2931	0.5109	−0.2917	−0.0855	0.0855	30,807,870,610.19	2,634,151,005.15
0.7500		0.7011	2.9065	0.0540	0.1802	0.2995	0.2995	0.5468	−0.2621	−0.0785	0.0785	45,614,619,429.19	3,581,019,733.64
0.8000		0.7371	3.8999	0.0513	0.3418	0.1502	0.1502	0.5751	−0.2403	−0.0361	0.0361	58,398,114,690.45	2,108,005,927.46
0.8500		0.7733	4.5371	0.0491	0.1634	0.3006	0.3006	0.5984	−0.2230	−0.0670	0.0670	44,940,201,576.61	3,012,889,148.78
0.9000		0.8095	4.5119	0.0468	−0.0056	−8.4172	8.4172	0.6185	−0.2087	−1.7564	1.7564	49,650,978,565.42	87,207,913,833.07

NB, The ${\left(GGP-\frac{DCIC}{G}\right)}^{2\left(\omega \right)}$ values used in the Table are the absolute values. Also, we used a ω value of 0.1.

### Hypothesis development

There is still little agreement on assessing and estimating central banks' reserves holdings or adequacy levels, even though this is a critical aspect of a country's external stability assessment (Assessing Reserve Adequacy—Specific Proposals, 2014). The literature on central banks' foreign exchange reserves holdings and adequacy levels have been vast, and all seek to address the common issue of how reserves can be perpetually built and made available for an intervention into the domestic economy whiles at the same time avoiding locking-up scarce resources in the reserves of central banks.

Ghana has been used as an experimental case to test the proposed reserve model in this research. The (Ghana, 2017) has bemoaned the lack of adequacy of Ghana's International reserves stock in satisfying the import metric benchmark of reserves, even though the reserves stock far exceeds the ceilings of other reserve metrics such as the Reserves-to-M2 metrics and the short-term debt metrics (Hongxing and Rahaman, 2018). This situation begs whether one reserve model is superior to others as a standard for reserve build-up. This couples with the fact that Ghana has been one of the most unstable economies in Sub-Saharan Africa, translating into high inflation and a frequent currency depreciation (Ghana, 2017). In addition, the choice of Ghana for this experimental study is because Ghana runs a managed currency regime system which makes proper management of the reserves a vital duty for the central bank.

The basic idea in this research is that a cost-effective reserve model should possess one of two characteristics, i.e., it must first have good directional predictive accuracy of economic variables, and second, it must have a near-perfect magnitude adjustment to economic variables. These criteria are necessary features of the reserve stock to stabilize the economy and make trade competitive in the domestic financial and goods market. Therefore, for any optimal reserve model to be effective, it must satisfy at least one of these two criteria.

On the first criteria, i.e., the directional predictive accuracy of economic variables, we tested the OPREM model in this research and compared it to the first group of research models that treat reserves as a fraction of a particular economic variable. The second group of researchers modeled the optimal reserves on cost-benefit analysis, so comparing this group and the OPREM model must consider both the first and second criteria of a good reserve model. This research focuses on the first criterion, which has led us to develop the OPREM model and compare alternative research models in the first group of researchers. This, therefore, implies that we only concentrate on the directional predictive accuracy of the models. The hypothesis below summarizes the test in this research.

**H1**_**0**_: The OPREM model has a better prediction of economic trends than the alternative optimal reserve models in the literature.
**H2**_**0**_: Incremental changes in the reserve holding set by the OPREM model are equal to the incremental changes in inflows.

## Specification of an econometric model

This section illustrates the OPREM model by conducting a simulation on Ghana using data from 2000 to 2016. The component or variable weights φ and ω in the OPREM model, which is not data collected or computed, are informed by the current structure of Ghana's reserves and the features of the managed currency regime practiced in Ghana. Also, this section compares the predictive accuracy of the OPREM model to trends in economic transactions. This comparison reveals the extent of exposure to economic shocks and vulnerability.

### Data

The variable used in this experiment is the monthly OPREM series which is the optimal reserve values produced by the reserve model developed in this research (Abdul-Rashid and Hongxing, 2022). Table 2 shows the research variables in deriving the OPREM model and their definitions.

**Table 2:** Research variables.

**Variable**	**Variable description**	**Source**
1.91_Treasury bill rates	91-day treasury bill rate (%)	Bank of Ghana
2. Exchange rates (for dollar, euro, and pounds)	End of Month exchange rate figures	Bank of Ghana
3. Gold prices (GGP)	Gold (US $/fine ounce)	Bank of Ghana
4. Currency in circulation (CIC)	Currency in Circulation (GHC'm)	Bank of Ghana
5. Number of Gold in reserves	Number of gold in ounce	World bank
6. Gross International reserves	Gross International Reserves ($million). Change to Ghana cedis	Bank of Ghana
7. Foreign direct investment (FDI)	Foreign direct investment (FDI) in Ghana cedi	World bank
8. Total imports	Total imports value of goods, services, and income (GSI) in Ghana cedis	World bank
9. Total external debts	Total external debts	World bank
10. Present values of Hard currency (HPV)	Currency gains now not utilized or invested	Computed
11. Future values of Hard currency (HFV)	Risk of currency depreciation not avoided	Computed
12. Inflows	Was defined as the simple average total of FDIs, total imports, and total external debts	Computed
13. Hard currency values (HCV)	Hard Currency values (HCV) mean the average exchange rate values of Ghana's key trading partners whose currencies have been employed in the computation of the Nominal Effective Exchange Rates (NEER) by the Bank of Ghana, that is, the U.S dollar rate, pound rates, and the Euro rates (Business and Financial Times, 2012; Ghana, 2017). The weights use in the computation of the NEER is based on Ghana's trade with the respective countries. However, the weight for the U.S dollars is taken as Ghana's trade with the rest of the world except Britain and Europe (Business and Financial Times, 2012).	Computed

The computed variables (i.e., HCV, HPV, HFV, and Inflows) are crucial in the OPREM model. The value of Hard Currency (HCV), at period *i*, is the simple average of the US, Euro, and Britain-Ghana exchange rates.


$$\begin{array}{l} HCV_{i}=\frac{ExR_{\frac{US}{GHc},\left(i\right)\;}+ExR_{\frac{Euro}{GHc},\;\left(i\right)}+ExR_{\frac{Britain}{GHc},\;\left(i\right)}}{3} \end{array}$$

To impute the volume of trade and Central Banks' holdings of foreign exchange into the *HCV*, it would have been more appropriate to use weighted averages (where the weight of each currency will represent the volume of trade between the foreign country and the home country). However, insufficient data or records of trade in most developing and emerging countries preclude us from using such an approach. For instance, the Bank of Ghana calculates Nominal Effective Exchange Rates, which uses the trade volume between its main trading partners as weights, but this information is sparingly determined. Therefore, to ensure standard application of the model, we propose a simple average approach of calculating the *HCV*.

The HPV, which is the variance of the present value (PV) of Hard currency, is computed as:


$$\begin{array}{l} HPV={\left[PV_{i}-\frac{\sum_{i=1}^{n}PV}{N}\right]}^{2} \end{array}$$

Where

${PV=HCV}_{i}{\left(1+r\right)}^{-i}\;$

*N* is the number of population, and
*i* is the period or year which is the same for all data frequencies falling within the same year or period.

Also, the HFV, the variance of the future value (FV) of Hard currency is:


$$\begin{array}{l} HPV={\left[FV_{i}-\frac{\sum_{i=1}^{n}FV}{N}\right]}^{2} \end{array}$$

Where

${FV=HCV}_{i}{\left(1+r\right)}^{i}\;$

*N* is the number of population,
*i* is the period or year which is the same for all data frequencies falling within the same year or period Supplementary Tables A, B, E shows the computations of *HPV*_*i*_, *HFV*_*i*_, and *HCV*_*i*_, respectively.

### Test of unit root

We must confirm that the OPREM series is stationary [i.e., I (0)] before specifying and estimating an econometric model for its analyses. A unit root test in Table 3 indicates the OPREM series is only stationary in the second difference, and therefore an I(2) process. We, therefore, transformed the series to the second difference.

**Table 3:** Unit root test.

**Order of** **integration I(D)**	**Augmented Dickey-Fuller** **(ADF) test statistics**			**Interpretation of results** **of the ADF test**
	**t-statistic**	**5% critical value**	**Probability**	
At level	3.390821	−3.828975	1.0000	Series not stationary
First differencing	1.498705	−3.933364	0.9998	Series not stationary
Second differencing	−8.047221	−3.828975	0.0001g^***^	Series now stationary

*** significant at 1% confidence level **significant at 5% *significant at 10%.

### Determining AR (*p*) and MA (*q*) terms

To select the Autoregressive (AR) and Moving Average (MA) order of the OPREM series, that is, *p* and *q*, respectively; we analyse both the Auto-correlations Functions (ACF) and the Partial Auto-correlations Functions (PACF) in the correlogram in Table 4A. The use of the correlogram in this research follows Box-Jenkins (BJ) methodology, where we select the ARMA terms [i.e., the AR(p) and MA(q) terms in economic time series data] with a correlogram.

**Table 4A:** Correlogram for ARIMA specification.

**No of Lags**	**Autocorrelation** **(ACF)**	**Partial correlation** **(PACF)**	**Q-Stat**	**PROB**	**Significant lags**
1	−0.552	−0.552	5.2447	0.022	✓
2	0.071	−0.335	5.3389	0.069	✓
3	0.005	−0.208	5.3393	0.149	–
4	0.028	−0.081	5.3575	0.253	–
5	−0.069	−0.117	5.4768	0.360	–
6	0.035	−0.099	5.5103	0.480	–
7	0.034	−0.010	5.5478	0.593	–
8	−0.070	−0.065	5.7331	0.677	–
9	0.035	−0.057	5.7877	0.761	–
10	−0.031	−0.099	5.8431	0.828	–
11	0.011	−0.102	5.8524	0.883	–
12	−0.019	−0.116	5.8921	0.921	–

The correlogram in Table 4A shows that the OPREM time series is an MA (2) process as the PACF Decays geometrically, and ACF is significant at lags 1 and 2 only. Gujarati (2004, p. 835) asserts that the BJ methodology is an iterative process and must consider estimating several alternative ARMA models with the determined model of the correlogram as the based model. Therefore, Table 4B shows the information criteria of the alternative combinations of AR(p) and MA(q) terms. Both Akaike Information Criterion (AIC) and the Schwarz Information Criterion selected ARMA (0, 1) as the best fit model instead of the based model of the correlogram, which is ARMA (0, 2). Therefore, the final fitted model is ARIMA (2, 0, 1) model. We, therefore, use the Autoregressive and Moving Average class of models to estimate this property of the OPREM series.

**Table 4B:** Information criteria of alternative models.

**Akaike information criterion (AIC)**						
**AR(p) Term**	**MA(q) Term**					
	**0**	**1**	**2**	**3**	**4**	**5**
0	−0.0317	−**0.1349**	−0.0307	0.0984	−0.0256	−0.0081
1	0.0183	−0.0288	0.1050	0.2199	0.3472	0.3417
2	0.1507	0.0938	0.0489	0.1264	0.3920	0.1890
3	0.2478	0.1285	0.3470	0.3686	0.3794	0.9500
4	0.3618	0.5110	0.3587	0.2610	0.4463	0.3356
5	0.0617	0.6021	0.0624	−0.0168	0.1128	0.1284
**Schwarz Information Criterion**						
**AR(p)**	**MA(q)**					
	**0**	**1**	**2**	**3**	**4**	**5**
0	0.1214	**0.0538**	0.2052	0.3816	0.3048	0.3694
1	0.2071	0.2071	0.3882	0.5503	0.7249	0.7665
2	0.3867	0.3771	0.3793	0.5040	0.8169	0.6610
3	0.5311	0.4589	0.7246	0.7934	0.8514	0.7693
4	0.6922	0.8886	0.7835	0.7331	0.9656	0.9020
5	0.4393	1.0269	0.5344	0.5023	0.6792	0.7421

The bold values are the values for which AR and MA are selected for the given criteria.

### Set-up of the empirical model and estimation

The general set-up of the ARIMA model is ARIMA (d, p, q), where *d* is the order of integration, *p* is the AR term, and *q* is the MA term. Therefore, the estimation of the model shown in Table 5 captures the MA terms but no AR terms.

**Table 5:** Model estimation output.

**Name of regressors in the model**	**Regression statistics**			
	**Coefficient**	***t*-statistics**	**Std errors**	***p*-value**
MA (1)	−0.9999	−3.22E-05	(31095.42)	1.000
Trend	0.0173	2.4348	0.0071	0.0331^**^
Constant	0.0922	1.8788	0.0490	0.0870^*^

** significant at 5%

* significant at 10%.

The estimated ARIMA model ARIMA (*d* = 2, *p* = 0, *q* = 1) on the OPREM series is, therefore:


(32) $$\begin{array}{l} oprem_{t}=\;\alpha_{0}\;+\text{MA}(1)+\gamma_{1}X_{t}+\;e_{t}\\ oprem_{t}=\;\alpha_{0}\;+\;\beta_{1}e_{t-1}\;+\gamma_{1}X_{t}+\;e_{t} \end{array}$$

Where

*X*_*t*_ is an exogenous trend variable imputed into the model.
α_0_ is a constant term.
*e*_*t*_ is the contemporaneous error term.
*e*_*t* − *i*_ is the lag error term or Moving Average (MA) term.

The model's estimate in Table 5 shows that the lag error regressors of the model are all statistically insignificant, and only the deterministic terms (that is, the constant and the trend) are significant at the 10% level. This statistic shows the OPREM series is a random walk with drift and a stochastic trend. The test outcome in Table 5 depicts the necessity of non-zero depletion of reserves and the significance of time, *t*, in the simulated country's optimal reserves build-up (Ghana).

The ACF and the PACF, together with the Ljung-Box test, can check serial correlations. The ACF and PACF correlogram test, therefore, show no serial correlation in the OPREM series. Table 6 shows the residual diagnosis test of this model.

**Table 6:** Residual diagnosis test.

**Test**	**Test-statistic**	***p*-value**	**Interpretation**
Heteroskedasticity—ARCH test	2.1629	0.1414	No heteroscedasticity
Normality test-Jarque Bera test	0.1881	0.9102	Series normally distributed
Serial correlation test	No serial correlation		

### Benchmarking the proposed reserve model (OPREM)

**Figure 2:**
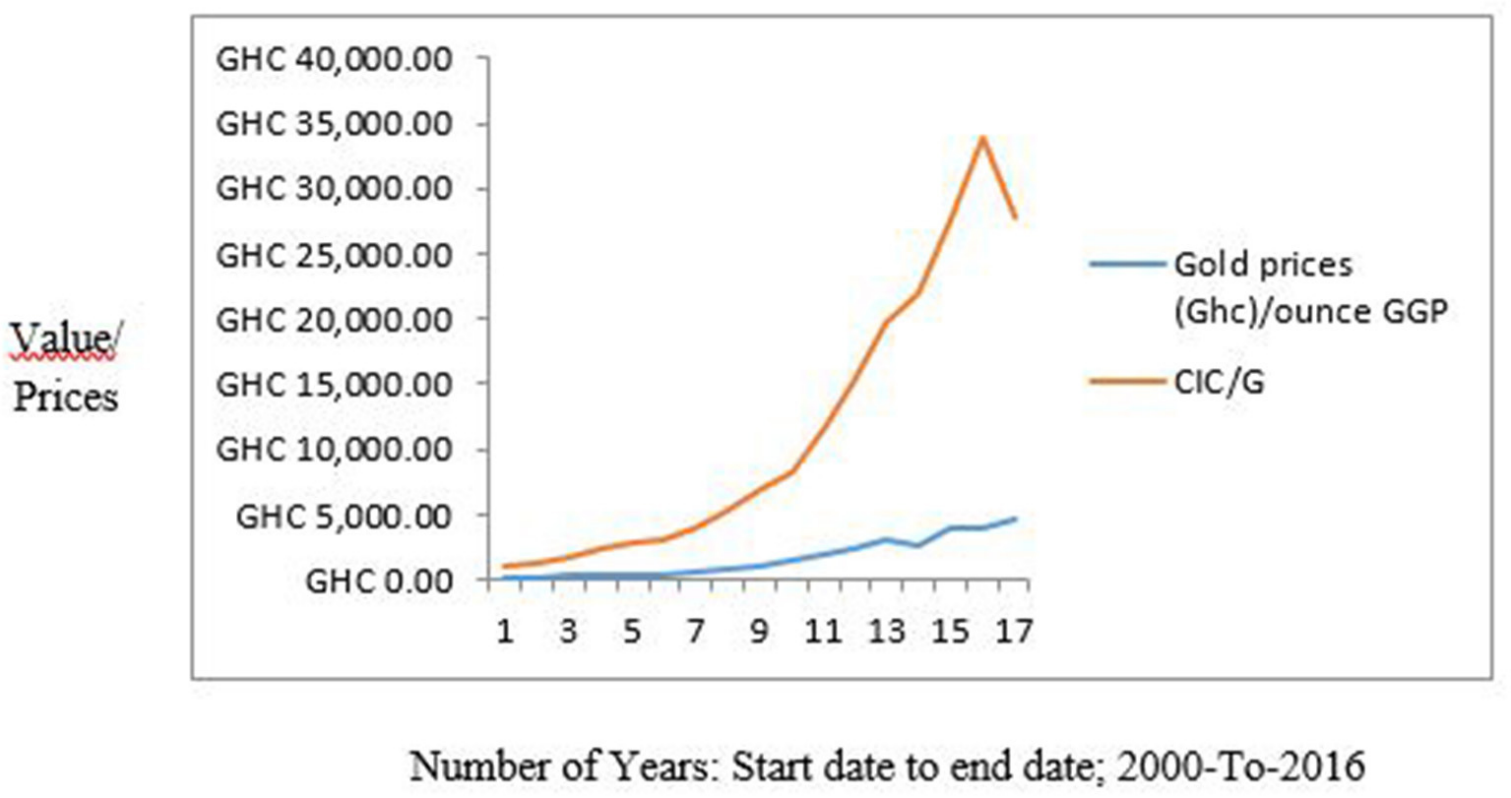
Average Opportunity cost method of Benchmarking the OPREM model.

Two methods were used in benchmarking the estimates of the OPREM model. The first method uses the average opportunity cost method, where each reserve stock component's opportunity cost is calculated and totaled. The average of the total opportunity cost is calculated and plotted on a line graph. Figure 2 shows a graph of a yearly analysis of this benchmarking and reveals that the average opportunity cost of the simulated country lies between a bandwidth of 0.8 and 1.2 of the *optimal reserve value* of the preceding period. These values then become the base model or strategy for the sensitivity analysis in Section Pesaran-Timmermann directional prediction test and sensitivity analyses test.

The average opportunity cost method is only applied in fixing the reserve stock when optimal reserves is calculated for successive times, i.e., *t* + *i*. Equation (33) therefore shows the estimate of the reserves in this benchmarking approach.


(33) $$\begin{array}{c} OPREM_{\left(t+i\right)}=\sum_{\min\;0.8}^{\max\;1.2}OPREM_{t} \end{array}$$

Where,

*OPREM*_*t*_ is the optimal reserve for the current period or time (*t*).
*i* is the number of periods ahead.

**Figure 3:**
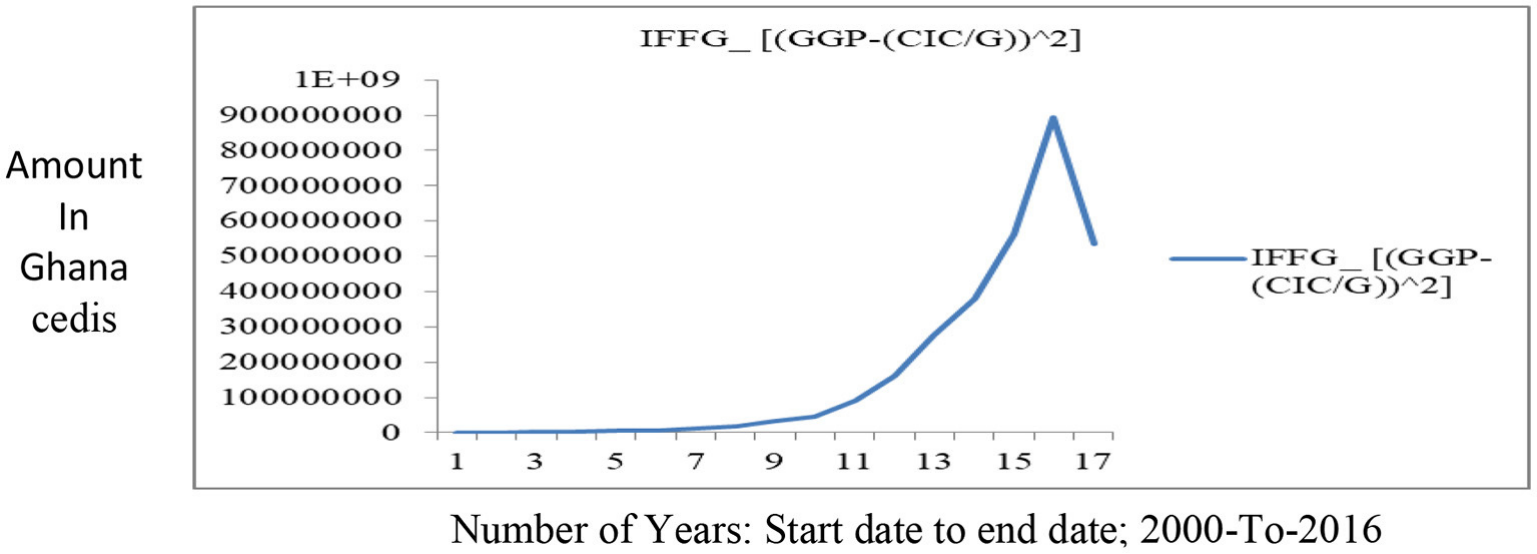
Ratio Benchmarking of the OPREM model.

Also, the second method employed in benchmarking *t*he OPREM is the ratio method. This ratio is expressed as a percentage of the average inflows in an economy. Figure 3 shows the range or width of the ratio benchmarking between 20 and 30% for the simulated country. Policymakers can apply this approach without prior knowledge of the preceding or current OPREM values. We will, therefore, use these values as the base measurement for the sensitivity analysis in Section Pesaran-Timmermann directional prediction test and sensitivity analyses test.

For the standard metrics in literature, the mathematical models of the import and broad money follow the benchmarks below:

#### Import metric or benchmark


(34) $$\begin{array}{c} Y_{t}=X_{t-1}+X_{t-2}+X_{t-3}+\varepsilon_{t}\\ Y_{t}=\sum_{i=1}^{3}X_{t-i}+\varepsilon_{t} \end{array}$$

Where:

*Y* is the Optimal Reserve Stock level.
*X* is the Import value.
*t* is the current time.
*i* is a lag period.
ε is the adjustment to the reserves in the current period (*t*).

And the Broad Money Metric or Benchmark is expressed as:


(35) $$\begin{array}{c} Y_{t}=0.2X_{t-i}+\varepsilon_{t} \end{array}$$

Where:

*Y* is the Optimal Reserve Stock level,
*X* is the Broad Money value,
*t* is the current time,
*i* is a lag period,
ε is the adjustment to the reserves in the current period (*t*).

The mathematical expressions above become the basis for the computations of the optimal reserve thresholds for the OPREM model, Import metric, and Broad Money (M2, M2+) metrics or benchmarks in the supplementary estimates in D1 and D2.

### Pesaran-Timmermann directional prediction test and sensitivity analyses test

Information usually is valid if we can explore it to improve decision processes (Blaskowitz and Herwartz, 2011). The upward and downward movement of economic variables is crucial for economic decisions, and therefore, forecasting or predicting the direction and trends of economic variables can provide practical frameworks for decisions.

Diebold and Mariano (1995) and Granger and Pesaran (2000a,b), among others, asserted that in evaluating the significance of forecasts, the realized economic value is often more logical than statistical values such as the mean squared error or the absolute forecast errors. Generally, these forecast criteria suffer from a lack of economic meaning and usually produce unreasonable forecasts far away from the realizations of the variable of interest (Armstrong and Collopy, 1992; Granger and Pesaran, 2000a,b; Pesaran and Skouras, 2002). Therefore, forecast evaluation criteria which relate to decision making, and are often reliant on forecasts of the directional change of the variable's up- or downward movements, are more beneficial (Cook, 2014).

In this study, we use a directional forecasting technique similar to Granger and Pesaran (2000a,b) and Pesaran and Skouras (2002) to compare the theoretical reserve metrics such as the B-G and H-models with the output of the OPREM model. The OPREM model in the test becomes the based model, and the underlying theories become the variations in the based model. The variations or changes to the based model are to observe the effects of parameter changes on the models' predictions on economic variables. This approach also tests which model (the based model or the alternative empirical models) best predicts the direction of economic transactions in the simulated country.

Our decision to observe the predictive effects of the models and parameter changes on changes in economic variables, but not on investor sentiments or sovereign risk, is because reserve build-ups or accumulation is supposed to protect against economic shocks or fluctuations. Therefore, the approach where reserve accumulation is matched against trends in economic variables is beneficial to monetary authorities and will enable the appropriate directional adjustments of central banks' reserves holdings. Besides, investor sentiments and sovereign risks are qualitative measures that are vulnerable to data subjectivity.

Pesaran and Timmermann (1992) used a non-parametric test to examine the effectiveness of a forecast to predict the direction of a change in a series of interests. This test computes the proportion of the forecast values that have the same sign as the actual series data. Assume the series of interest, denoted as *y*_*t*_ and its forecasts are *x*_*t*_, the Pesaran-Timmermann test (*S*_*n*_) is defined as:


(36) $$\begin{array}{c} S_{n}=\frac{\hat{p}-\hat{p_{{\;}^{*}}}}{{\left[\hat{V}\hat{\left(P\right)}-\hat{V}\left(\hat{P_{{\;}^{*}}}\right)\right]}^{0.5}} \end{array}$$

Where

*S*_*n*_ is the standard test statistic,
$\hat{p}$ is the proportion of times that the sign of *y*_*t*_ is predicted correctly,
*P*_*y*_ is the probability of y,
*P*_*y*_ is the probability of x.

Note, therefore, that:

$\hat{P}=n^{-1}\overset{n}{\underset{t=1}{\sum }}I\left(y_{t}x_{t}\right)$

${\hat{P}}_{*}=\hat{P_{y}}\hat{P_{x}}+\left(1-\hat{P_{y}}\right)\left(1-\hat{P_{x}}\right)$

$\hat{V}\left(\hat{P}\right)=n^{-1}{\hat{P}}_{*}\left(1-{\hat{P}}_{*}\right)$

$\hat{V}\;{\hat{P}}_{*}=n^{-1}{\left(2{\hat{P}}_{y}-1\right)}^{2}\left(1-{\hat{P}}_{x}\right)+n^{-1}{\left(2{\hat{P}}_{x}-1\right)}^{2}$

$\hat{P_{y}}\left(1-\hat{P_{y}}\right)+{4n}^{-2}\hat{P_{y}\hat{P_{x}}\left(1-\hat{P_{y}}\right)\left(1-\hat{P_{x}}\right)}$

$\hat{P_{y}}=n^{-1}\overset{n}{\underset{t=1}{\sum }}I\left(y_{t}\right)$, and $\hat{P_{x}}=n^{-1}\overset{n}{\underset{t=1}{\sum }}I\left(x_{t}\;\right)$

$I\left(\cdot \right)=\{\begin{array}{c} 1\\ 0 \end{array},\begin{array}{c} If\;\cdot \;>0\\ otherwise \end{array}$

Under the null hypothesis of *x*_*t*_ not been able to predict *y*_*t*_. Note that *S*_*n*_ also follows the standard normal distribution.

Table 7 shows the Pesaran-Timmermann test statistic (PT-test) of the reserve models' directional predictions to the trends in economic variables such as the Composite Index of Economic Activity, Inflation, and Interest rate differentials in Ghana. The test output suggests that the OPREM model, which is the based model, predicts the direction of the economy better than the alternative models.

**Table 7:** Pesaran-Timmermann test statistics.

**Economic series-to-benchmark**	**Monthly data**		**Remarks**
	**Mean Square error**	**PT test Abs. Value**	
CIEANominal—OPREM	1,037	10.35	OPREM has higher directional accuracy than other benchmarks
CIEANominal—Import Benchmark	91.35	3.91	
CIEANominal–20%M2 Benchmark	25.85	4.91	
CPI_O—OPREM	904.03	7.33	OPREM has higher directional accuracy than other benchmarks
CPI_O—Import Benchmark	84.23	3.12	
CPI_O–20%M2 Benchmark	19.02	3.33	
IntDiff—OPREM	927	13.82	OPREM has higher directional accuracy than other benchmarks
IntDiff—Import Benchmark	101	9.20	
IntDiff–20%M2 Benchmark	45	9.37	

CIEANominal is a composite index of economic activity, CPI_O is consumer price index overall statistics, and IntDiff is Interest rate differentials.

However, when considering the Mean Square Errors or standard forecast measures, the OPREM model was the worst performer. These standard measures are but only statistical measures that calculate the variations in the variables and models in absolute numbers. Diebold and Mariano (1995) and Granger and Pesaran (2000a,b) asserted that these statistical measures suffer from a lack of economic meaning. This is why the Mean Square Errors were not used in the decision. Besides, the decisions whether to withdraw or increase the reserve stock is central to the building of the reserve stock, and predicting the direction of flow (i.e., either increase or decrease), using the PT- test statistic, is essential.

#### Sensitivity analysis of the OPREM test

Assessing the sensitivity of the OPREM model to changes in the weights and benchmarks is relevant to policy decisions. Firstly, we assess the chance or likelihood of the optimal value computed from the OPREM model falling within the domain of the benchmarks given different data frequencies. Tables 8A,B show these likelihoods or probabilities for different strategies (including the based strategy or benchmark) under the opportunity cost approach to benchmarking in Section Benchmarking the proposed reserve model (OPREM). Interpreting these probabilities, we observed that the benchmark values in strategies 1 and 2 in Table 8A would return the same likelihood values when the data frequency is <7. However, strategy 1 has a slight edge over strategy 2 when the frequency of the data is between 7 and 13, and much superior when data frequency exceeds 13. This implies that the two strategies give the same results when the analysis period is short but differs over prolonged periods. Therefore, strategy 1 increases the likelihood of the optimal value falling in the benchmark domain in long-term periods or observations (i.e., periods exceeding 13 data frequencies). Besides the robustness of strategy 1 in reining in the optimal solutions of the OPREM model, Pannell (1996) asserts that the significance of these decisions or changes from the based solution(s) must be evaluated. Therefore, we employed the Spearman Rank Correlation test and the *t*-test of significance in Table 9 to assess whether the difference in strategy 1 and strategy 2 is anything significant.

**Table 8A:** Probabilities of the OPREM meeting the lower band width given the data frequency.

**Data Frequency (DF)**	**Strategy**			
	**S1 (0.7)**	**Based strategy** **S2 (0.8)**	**S3 (0.9)**	**S4 (1.0)**
DF < 7	90.1	90.1	87.3	54.9
7 ≤ DF < 13	98.6	97.2	86.1	53.6
13 ≤ DF < 17	95	86.7	76.7	55

DF, Data Frequency; N, Number of optimal values; Prob., Probabilities. The red color indicates or marks the highest probability for each data frequency and strategy combination.

**Table 8B:** Probabilities of the OPREM meeting the higher band width given the data frequency.

**Data frequency (DF)**	**Strategy**					
	**S1 (1.0)**	**S2 (1.1)**	**Based Strategy** **S3 (1.2)**	**S (1.3)**	**S5 (1.4)**	**S6 (1.5)**
DF < 7	45	86	90	90	92	92
7 ≤ DF < 13	44	83	93	94	94	97
13 ≤ DF < 17	79	85	92	92	93	93

DF, Data Frequency; N, Number of optimal values; Prob., Probabilities. The red color indicates or marks probabilities above or equal to 90% for each frequency and strategy combination.

**Table 9:** Spearman rank test and the *t*-test of significance.

**Boundary**	**Spearman rank, r_s_, coefficient**	**t_c_**	**t_T_** **DF=n − 1, α = 0.05 (two-tail test)**	**Decision:** **H_0_: The ranks of one variable do not co-vary with the ranks of other variables**.
Lower Band	0.5 (S1, S2)	0.5773	4.303	The ranks between S1 and S2 co-vary significantly from one another
High Band	1 (S1, S2, S3, S4)	1	4.303	The ranks between S1 and S2 co-vary significantly from one another

n, number of observations; 3 DF, Degree of Freedom Decision Rule: if *t*_*c*_ > *t*_*T*_; reject the null hypothesis, where *t*_*c*_ is t-test calculated, and *t*_*t*_ is t-test from the statistical table.

The results in Table 9 conclude that the two strategies are significantly different and should be implemented. Therefore, we will be revising the lower band of the opportunity cost approach to 0.7 (i.e., strategy 1).

Also, for the higher bandwidth values in Table 8B, four different strategies, including the based strategy, can produce a 90% chance or likelihood of the optimal value falling within the benchmark domain. These four strategies have their best performance when the data frequency is more than 13, and the differences between them are also significant. However, because each of these strategies has met a minimum of 90% likelihood rate, we decided to maintain the based strategy to avoid holding excess reserves in most of the periods. Therefore, the complete benchmark of the OPREM model, using the opportunity cost approach, is 0.7–1.2 of the immediate pass period's reserves.

Furthermore, the sensitivity of the ratio approach of benchmarking to the variations in different strategies has been analyzed in Tables 10A,B. In Table 10A, strategy 1 and 2 in the lower band statistics both achieves a 100% chance of the optimal values falling in the domain when the frequency of data analyzed is <7, or between 7 and 13. However, when data frequency exceeds 13, the two strategies produce different results. Strategy 3 in Table 10A also has over a 90 per cent chance of reining in the optimal solution, except at the point where data frequency exceeds 13. For the upper band values, in Table 10B, all the strategies, except strategy 1, exceed a minimum of 95 per cent chance of the optimal value falling within the solution domain when data frequency is <7 or between 7 and 13. Since Table 11 shows all the strategies to be significantly different, we will revise the lower band value in the ratio analysis to 2 (i.e., strategy 1) because it produces a 100 per cent likelihood for both short and long period observations. Also, for the upper band of the ratio analysis, we will maintain the based strategy (which is 6^*^inflows) for only short period analysis and strategy 5 (which is 9^*^inflows) for long period analysis or computations. Therefore, the ratio analysis will benchmark the OPREM model as falling between 2 and 6 times of the average inflows in an economy when period or observations are small and between 2 and 9 times of the average inflows when data frequency or period is large.

**Table 10A:** Probabilities of the OPREM meeting the lower band width value given the data frequency (ratio analysis).

**Data frequency (DF)**	**STRATEGY (S)**		
	S1 (2*inflows)	**Based Strategy** S2 (3*inflows)	S3 (4*inflows)
DF < 7	100	100	99
7 ≤ DF < 13	100	100	94
13 ≤ DF < 17	100	93	83

The red color indicates or marks probabilities above or equal to 90% for each frequency and strategy combination.

**Table 10B:** Probabilities of the OPREM meeting the higher band width value given the data frequency (ratio analysis).

**Data frequency (DF)**	**Strategy**				
	S1 (5*inflows)	**Based strategy** S2 (6*inflows)	S3 (7*inflows)	S4 (8*inflows)	S5 (9*inflows)
DF < 7	21	100	100	100	100
7 ≤ DF < 13	49	99	100	100	100
13 ≤ DF < 17	43	47	57	70	100

The red color indicates or marks probabilities above or equal to 90% for each frequency and strategy combination.

**Table 11:** Spearman Rank test and the *t*-test of significance.

**Boundary**	**Spearman Rank, r_s_, coefficient**	**t_c_**	**t_T_ DF=n − 1, α = 0.05 (two-tail test)**	**Decision:** **H_0_: The ranks of one variable do not co-vary with the ranks of other variables**.
Lower band	0.5 (S1, S2)	0.5773	4.303	The ranks between S1 and S2 co-vary significantly from one another
Lower band	−0.5 (S1, S3)	−0.5773	4.303	The ranks between S1 and S2 co-vary significantly from one another
High band	0.75 (S5, S4)	1.1339	4.303	The ranks between S1 and S2 co-vary significantly from one another
High band	1 (S4, S3)	1	4.303	The ranks between S1 and S2 co-vary significantly from one another
High band	0.5 (S3, S2)	0.5773	4.303	The ranks between S1 and S2 co-vary significantly from one another

n, number of observations; 3 DF, Degree of freedom decision rule: if *t*_*c*_ > *t*_*T*_, reject the null hypothesis, where *t*_*c*_ is t-test calculated, and *t*_*t*_ is t-test from the statistical table.

## Results and discussion

The OPREM test developed in this research has a higher directional predictive accuracy of the economy than the B-G and H-models' predictions. This outcome means that the reserves holding of the OPREM model enables better adjustments to economic trends. Therefore, the OPREM model satisfies the necessary condition of a reserve stock being endogenously determined by the economy. This feature is good for the systematic building of the reserve stock by monetary authorities and central banks. Also, the high probabilities or likelihood of the optimal solution falling within the domain of the benchmarked strategies shows the robustness of the benchmarks in approximating the optimal solution of the OPREM model.

For matching the reserve stock to economic trends, the research emphasizes directional predictions of economic variables rather than the magnitude (which is measured by the Mean Square Errors) because the reserves' adjustments do not have to match the changes in economic variables precisely. This argument is so because the reserve stock operates as a fraction of a country's economic activities (fractional system). Therefore, an optimal reserve stock will not mean matching the perpetual building of the reserves to changes in the actual amount or value of economic transactions. In this regard, we established the superiority of the OPREM model to the B-G and H-models which do not have the same resilience, adjustments or robustness to changing economic conditions or trends. For example, the OPREM model in the component plot reflected the gains in the economy during the reclassification of Ghana as a Highly Indebted Poor Country (HIPC). It also did not fail to adjust the reserve stock and show the effects of the Global Financial crisis on developing countries. Furthermore, the OPREM model highlighted the dip in the cedi exchange rate from 2013 to 2016 and adjusted the reserve stock to play the protective cover role, which was the focus of the research in Abdul-Rashid and Yao (2019). These automatic adjustments toward economic trends and the quantum adjustment of reserves make the OPREM model superior to the existing reserve models.

To elucidate further on the automatic and systemic adjustments which are inherent in the OPREM model, we assessed the OPREM model and the impact major economic events in Ghana would have had in the building of the reserve stock:

i. Analyzing the graph plots of the various components of the reserve stock in the OPREM model and how each component automatically adjusts to the trends in the economy, the component plot in Figure 1B shows relative stability in Ghana's currency gains that were not utilized but kept in the reserves from 2000 to 2007. During these periods, the gains achieved in the currency market were primarily due to strict adherence to the fiscal conditions inherent in the HIPC conditions (Asiama et al., 2014; National Development Planning Commission, 2014; Nyeadi et al., 2014). This discipline in fiscal policies prevented massive depreciation of the cedi and narrowed the absolute values of the domestic and hard currencies. This made the trade-off between hard currencies and the domestic currency relatively stable, thereby stabilizing the opportunity cost of holding hard currencies in the reserve portfolio during those years. Also, currency depreciation that was not avoided during these periods was relatively stable. This stability was more due to stable relative interest rates or the cost of capital across different periods. Therefore, the period between 2000 and 2007 was when the marginal benefit of adding hard currency to the reserves stock did not have increased opportunity cost threats.
ii. Additionally, the OPREM model captured the effects of the global financial crisis and the highest turbulent periods in Ghana's exchange rate history. The global financial crisis severely affected the currencies of developed nations but did not negatively impact the currencies of most developing countries. Indeed, the currency of some developing countries like Ghana strengthened during the crisis but was primarily due to the negative impact of the crisis on developed countries' currencies. This situation made holding hard currencies in the portfolio of reserves, from 2007 to early 2009, a wrong decision for monetary authorities to take. This was because the opportunity cost of keeping hard currencies had increased and needed to be traded-off for other securities that promise higher yields and high liquidity. This scenario saw a decline in 'currency depreciation that needs to be avoided, that is, the depreciation of hard currencies in Ghana's central bank's reserves stock during 2007–2009.
iii. Furthermore, the years between 2013 and 2016 were high threshold periods in the cedi rates. This epoch led to a sharp decline in the exchange rate. Under this circumstance, the benefits of foreign exchange reserves in restoring the cedi's value were necessary for the adjustment process, and holding more reserves under this circumstance was a better option for the central bank. Therefore, the effectiveness of the proposed reserve model in this research is in its natural adjustments to conditions in the currency market and global trends. For example, consider how, during the period between 2007 and 2008, hard currencies were depreciating relative to the domestic currency and how the proposed reserve model avoided the depreciating hard currency by not keeping them in the central bank's reserves. The falling CDA_FV curve shows this in Figure 1B.
iv. Consider also how hard currencies were appreciating to domestic currency during the years between 2013 and 2016. The proposed model again adjusted to keep these hard currencies in the reserves of the central bank. This slightly higher CGU_PV curve in Figure 1B shows this scenario. The CGU_PV and the CDNA_FV curves are mirror opposite of one another. Again, both curves are significant in determining the amount of hard currency in the reserves.

**Figure 4:**
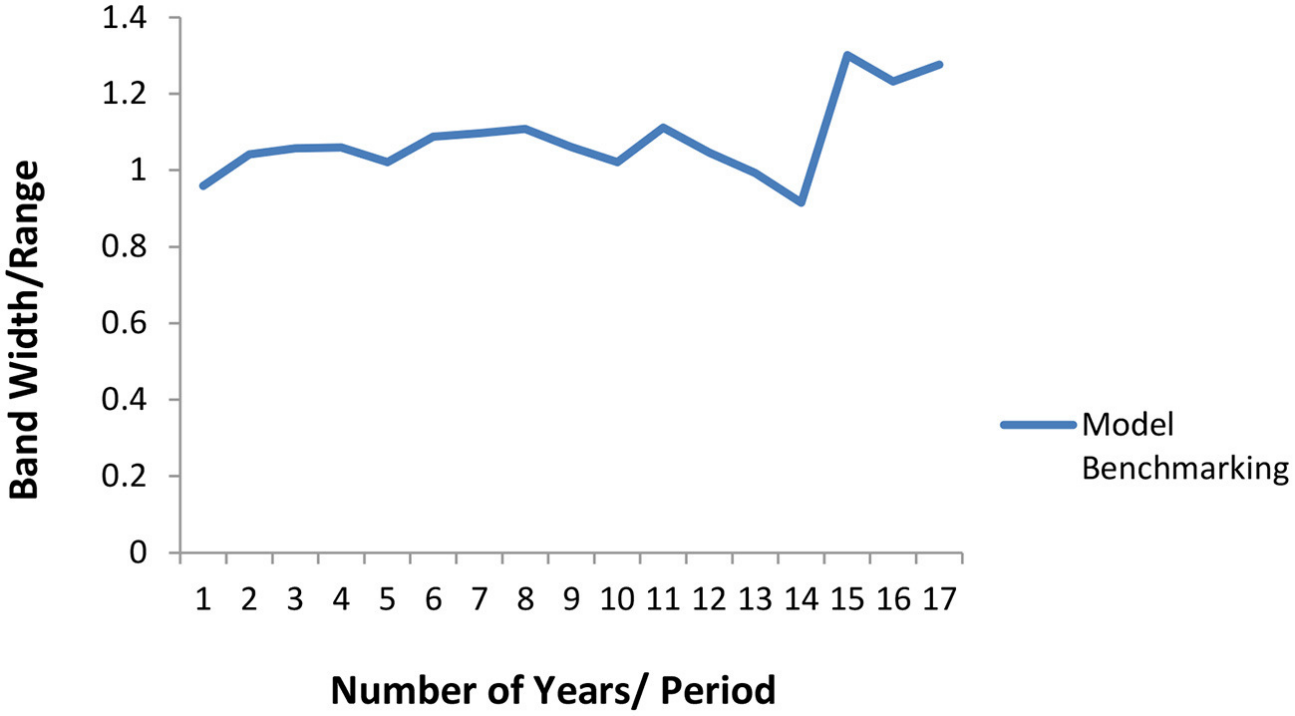
Plot of OPREM Gold Impact Factor components series (Component analysis).

Another essential component or constituent of the reserve portfolio is gold. The amount of currency issued by a country represents a sovereign debt. Therefore, the country is liable to holders of their currency at an amount equal to the face value of the currency. A country's ability, backed by the amount of gold in reserve, is a good and a traditional method of determining liquidity or default risk and the volatilities of a country's currency. The amount of gold in the reserves of central banks was instrumental for the sustenance of the fixed exchange rates regime before the break of World War II and still has some significance in other currency regimes though at a reduced level. The Impact Factor of Gold (IFFG) measures the degree to which a country's currency is strongly backed by gold. Figure 4 shows the plot of the components of the gold impact factor and their linear trends.

**Figure 5:**
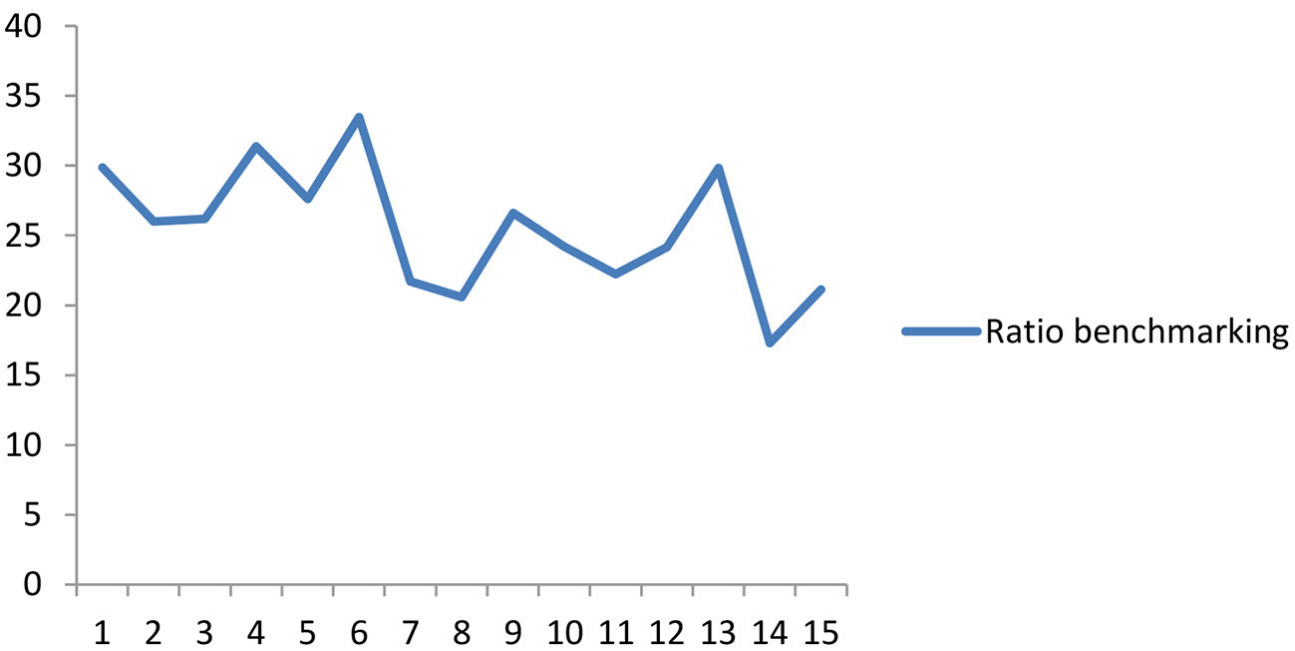
Plot of OPREM Gold Impact Factor SERIES.

The gold impact factor shows the risk of default and currency volatility in the holders' hands. An upward trend in the Impact factor of gold (IFFG), in Figure 5, means more volatility and risk, and a downward trend in IFFG means less volatility and risk in the reserves.

Ghana's gold reserves have remained steady with only marginal increases, while currency in circulation (M1) has been sky-rocketing. This leaves Ghana with a sharp growth in the gold impact factor curve and hence exposes the currency to high volatilities and risk of default for holders of the Ghanaian cedi.

**Figure 6:**
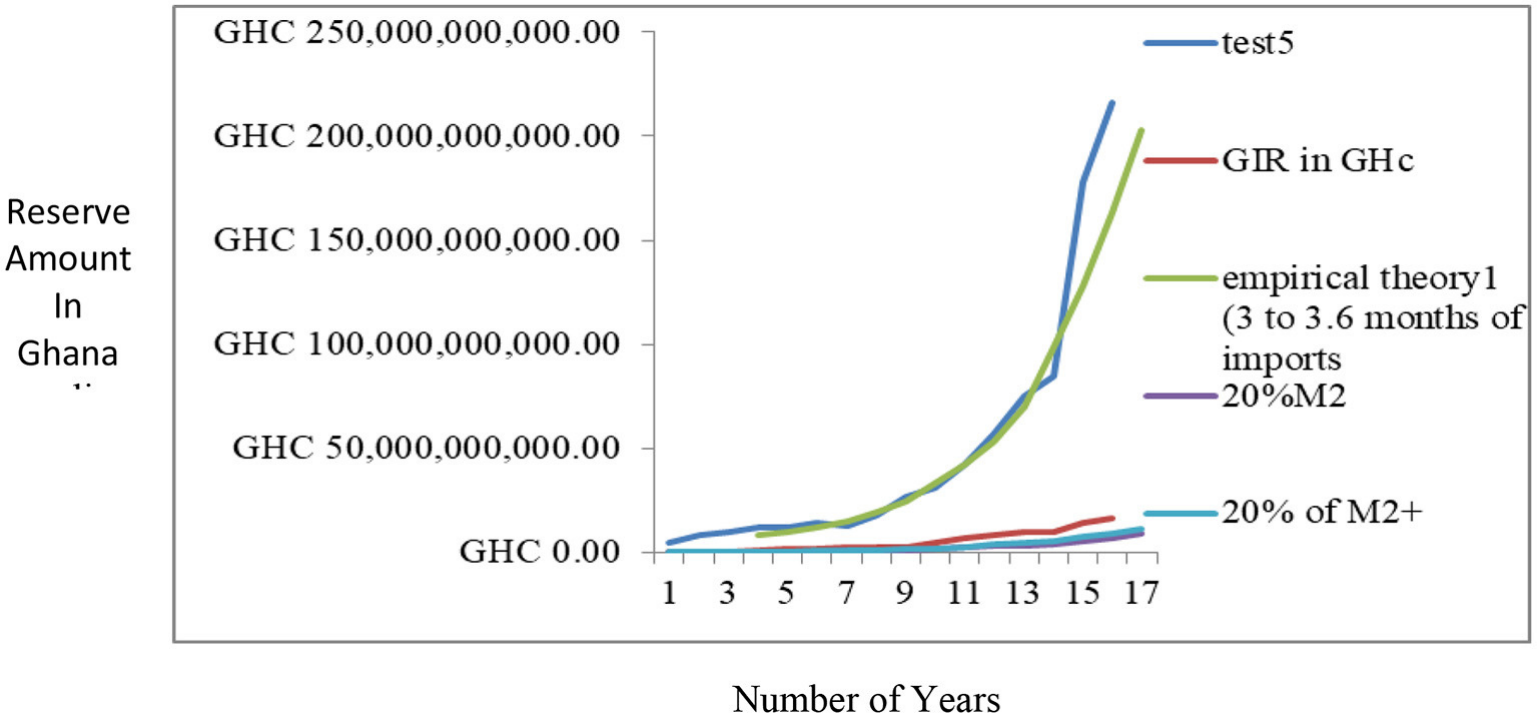
Optimal Model (OPREM) compared to other empirical benchmarks (YEARLY ANALYSIS).

Figure 6 shows the proposed reserve model's output results (OPREM) and output of alternative reserves models. The OPREM model's result is very much similar to the optimal reserves proposed by the import benchmark.

## Conclusions

Inferring from the derivatives and conditions held in Section Partial derivative of the OPREM model, we conclude that during periods of currency stability, the cost of holding reserves for precautionary purpose is optimized by overlooking the prevailing interest and inflationary rates and only considering these two variables; (1) the percentage trade-offs between global gold prices and hard currency values, and (2) the economic value of gold, defined as the output of domestic currency in circulation divided by the amount of gold in reserves. These variables only defined the optimal reserves in a stable currency or exchange rate environment because, the effect of hard currencies in the OPREM model will have zero dispersion or standard deviation and therefore the variance too will be zero. This zero variance will render insignificant whatever number, i.e., the interest and inflation digits, that is raised as an exponent. Therefore, in a stable environment, the only random variable in the reserve model is the gold impact factor.

Also, further investigations and analyses of the OPREM model has revealed that the OPREM test has superior directional predictive accuracy of the economy than the B-G and H-models. Therefore, we find that the OPREM model fixes reserves that are adjustable to economic conditions. This adjustability is an essential feature of a reserve, and it is also fundamental to its effectiveness as an emergency or control fund. Considering the threshold periods and event analysis in the simulated country, the OPREM model auto-adjusted the reserves to match the changes in economic conditions. Therefore, the OPREM is better positioned in eliminating procyclicality and reserve build-ups in developing and emerging countries (Aizenman and Sun, 2012; Pina, 2015; Abdul-Rashid and Yao, 2019).

Also, compared to existing reserve models, the OPREM model fixes reserves that are very close to only the import model. However, the import model does not have the same superior adjustments as the OPREM model. These inferior adjustments make all other reserve models, such as the import and reserve-to-M2 metric, only compete in accumulating reserves when even economic trends suggest a downward revision of the reserves.

Investigations reveal that the OPREM model for the simulated country is a random walk model with a drift and a stochastic trend. This property points or directs the simulated country to two things, i.e., how reserves are/should be managed (the management of reserves) in the simulated country and the relevance of reserves in the simulated country. To the extent that reserve remains relevant in an economy, this reveals weaknesses in the financial and banking sector (Roger, 1993; Assessing Reserve Adequacy—Specific Proposals, 2014). This conclusion of financial and banking sector weaknesses in the simulated country is consistent with the “Bank of Ghana, BoG” (2017) and the Ghana (2017).

Lastly, to avoid repeating large volumes of computations of the OPREM model, a benchmark of 0.7–1.2 of the immediate pass optimal value. This approach will need knowledge of the previous year's optimal value. However, in the absence of this knowledge, a benchmark between 2 and 6 times of the average inflows in an economy is recommended for short-term analysis or analysis with small data observations. However, for long-term analysis or analysis with large data frequency (i.e., exceeding 13 data observations), the reserve stock should be fixed on a benchmark of 2–9 times of the average inflows.

## Data availability statement

Publicly available datasets were analyzed in this study. This data can be found here: https://www.openicpsr.org/openicpsr/project/178621/version/V1/view.

## Author contributions

A-RA-R in charge of methodology, conceptualization, and writing and organizing manuscript. YH conceptualized the research and contributed to the methodology. A-RA participated in test running and analysis. EA also in test running and analysis. JB contributed in writing parts of the manuscripts. MA contributed the methodology, conceptualization, and writing of the research. All authors contributed to the article and approved the submitted version.

## Funding

This work was supported by the National Natural Science Foundation of China Nos. (71701082 and 71271103).

## Conflict of interest

The authors declare that the research was conducted in the absence of any commercial or financial relationships that could be construed as a potential conflict of interest.

## Publisher's note

All claims expressed in this article are solely those of the authors and do not necessarily represent those of their affiliated organizations, or those of the publisher, the editors and the reviewers. Any product that may be evaluated in this article, or claim that may be made by its manufacturer, is not guaranteed or endorsed by the publisher.
