# Supplementary material for: Optimizing foreign exchange reserves: Protection against external shocks in Ghana
Source: Front Psychol. 2022 Nov 2;13:994043. doi: 10.3389/fpsyg.2022.994043 (PMC9671775; doi:10.3389/fpsyg.2022.994043)
Supplement: Supplementary file 1 [file Data_Sheet_1.pdf]

SUPPLEMENTARY MATERIAL

Supplementary A1 Currency Gains now not Utilized or Invested (Monthly Analysis)

Date	Time (t)	Hard Currency Value (HCV)	Discounted Rate $(1+r)^{-t}$	Present Value (PV)	Variance Of PV	Assigned Weight (W)	HPV (weighted PV RESULT)
2000	1	0.5865	0.745212013	0.437066846	0.002363566	0.7	0.014504319
2000	1	0.4503	0.745212013	0.335568969	0.002796438	0.7	0.016316375
2000	1	0.481566667	0.745212013	0.358869265	0.000875039	0.7	0.00723468
2000	1	0.528166667	0.744823477	0.393390933	2.44096E-05	0.7	0.000590598
2000	1	0.6749	0.739152931	0.498854313	0.012189039	0.7	0.045727624
2000	1	0.6104	0.695313586	0.424419413	0.001293775	0.7	0.009512641
2000	1	0.698933333	0.686436024	0.479773018	0.008339833	0.7	0.035059859
2000	1	0.731266667	0.69827526	0.510625422	0.014926753	0.7	0.052695768
2000	1	0.7347	0.706214689	0.518855932	0.017005621	0.7	0.057731753
2000	1	0.749433333	0.704572677	0.52803025	0.019482554	0.7	0.063496841
2000	1	0.7602	0.704274949	0.535389816	0.021591212	0.7	0.068232914
2000	1	0.776833333	0.704274949	0.547104256	0.025171068	0.7	0.07596808
2001	2	0.7943	0.496003204	0.393975345	3.05258E-05	0.7	0.000690664
2001	2	0.807066667	0.496003204	0.400307652	0.000140596	0.7	0.002011784
2001	2	0.8028	0.477598482	0.383416061	2.53439E-05	0.7	0.000606333
2001	2	0.802233333	0.472490864	0.379047921	8.84053E-05	0.7	0.001453901
2001	2	0.8001	0.465489431	0.372438093	0.000256392	0.7	0.003063605
2001	2	0.788433333	0.462770142	0.364863406	0.000556343	0.7	0.005269141
2001	2	0.783	0.471194415	0.368945227	0.000380449	0.7	0.004038383
2001	2	0.793333333	0.503921596	0.399777799	0.000128312	0.7	0.001887061
2001	2	0.799833333	0.531938885	0.425462451	0.001369897	0.7	0.009901057
2001	2	0.804833333	0.552948392	0.445031297	0.003201406	0.7	0.017936533
2001	2	0.803133333	0.574791595	0.46163429	0.005355892	0.7	0.025714747
2001	2	0.809466667	0.601484816	0.486881909	0.009688776	0.7	0.038939308
2002	3	0.812866667	0.493767442	0.401367095	0.000166843	0.7	0.002267855
2002	3	0.820833333	0.533470944	0.437890733	0.002444353	0.7	0.014849602
2002	3	0.827733333	0.529595868	0.438364153	0.00249139	0.7	0.015049053
2002	3	0.8546	0.527033311	0.450402668	0.003838092	0.7	0.020364829
2002	3	0.880133333	0.527033311	0.463859585	0.005686556	0.7	0.026816032
2002	3	0.903433333	0.513230769	0.463669784	0.005657966	0.7	0.026721587
2002	3	1.0094	0.510773163	0.515574431	0.016160537	0.7	0.05570819
2002	3	0.9584	0.498717652	0.477970998	0.00801395	0.7	0.034095152
2002	3	0.9625	0.499906018	0.481159542	0.008594998	0.7	0.035807339
2002	3	0.969766667	0.498717652	0.483639755	0.009061027	0.7	0.037155592
2002	3	0.9813	0.498717652	0.489391632	0.010189146	0.7	0.040336337

Date	Time (t)	Hard Currency Value (HCV)	Discounted Rate $(1+r)^{-t}$	Present Value (PV)	Variance Of PV	Assigned Weight (W)	HPV (weighted PV RESULT)
2002	3	1.0012	0.492831982	0.49342338	0.011019341	0.7	0.042609745
2003	4	1.0642	0.386832528	0.411667176	0.000539022	0.7	0.005153764
2003	4	1.0524	0.379596577	0.399487438	0.000121818	0.7	0.00181969
2003	4	1.086333333	0.362233265	0.39350607	2.55605E-05	0.7	0.000609956
2003	4	1.087666667	0.319590187	0.347607594	0.001668129	0.7	0.011364773
2003	4	1.0855	0.31482915	0.341747043	0.002181197	0.7	0.013711546
2003	4	1.099633333	0.298406867	0.328138138	0.003637561	0.7	0.019614045
2003	4	1.094766667	0.265580103	0.290748244	0.009545698	0.7	0.038535888
2003	4	1.0819	0.361111367	0.390686388	4.99995E-06	0.7	0.00019466
2003	4	1.080066667	0.376042049	0.406150483	0.000313295	0.7	0.00352507
2003	4	1.111166667	0.379596577	0.421795064	0.001111871	0.7	0.008555357
2003	4	1.122933333	0.425713499	0.478047878	0.008027721	0.7	0.034136153
2003	4	1.156033333	0.488737071	0.564996345	0.031168495	0.7	0.088226913
2004	5	1.2046	0.463988533	0.558920587	0.029060108	0.7	0.084005566
2004	5	1.221766667	0.440832442	0.538594383	0.022543237	0.7	0.070325267
2004	5	1.215766667	0.431594873	0.52471866	0.018569058	0.7	0.061397813
2004	5	1.205833333	0.437109216	0.527080863	0.019218425	0.7	0.06289302
2004	5	1.198833333	0.458065353	0.549144014	0.02582246	0.7	0.07733896
2004	5	1.216866667	0.458065353	0.557404459	0.028545498	0.7	0.08296145
2004	5	1.225333333	0.458065353	0.561282746	0.029871044	0.7	0.085639728
2004	5	1.219033333	0.456111152	0.556014698	0.028077817	0.7	0.082007645
2004	5	1.2107	0.456111152	0.552213772	0.026818465	0.7	0.079415219
2004	5	1.220866667	0.454166947	0.554477287	0.02756495	0.7	0.080956188
2004	5	1.2574	0.454166947	0.57106952	0.033349768	0.7	0.09250495
2004	5	1.286033333	0.454166947	0.584073833	0.038268555	0.7	0.101856663
2005	6	1.2635	0.387845386	0.490042646	0.010320998	0.7	0.040701011
2005	6	1.258433333	0.38586406	0.485584196	0.009434988	0.7	0.038222487
2005	6	1.2774	0.38586406	0.492902751	0.010910308	0.7	0.042314178
2005	6	1.263866667	0.383894533	0.485191504	0.009358855	0.7	0.038006326
2005	6	1.251066667	0.383894533	0.480277654	0.008432257	0.7	0.035331388
2005	6	1.223866667	0.406221805	0.497161327	0.011818081	0.7	0.044748952
2005	6	1.199266667	0.419037602	0.502537828	0.013015957	0.7	0.047877699
2005	6	1.2164	0.436866428	0.531404323	0.020435844	0.7	0.065656058
2005	6	1.2212	0.460411781	0.562254867	0.030208017	0.7	0.086314856
2005	6	1.200433333	0.48804122	0.585860948	0.038970952	0.7	0.103161752
2005	6	1.176233333	0.498564627	0.586428333	0.03919529	0.7	0.103577092
2005	6	1.1928	0.523225444	0.624103309	0.055532326	0.7	0.132184979
2006	7	1.206466667	0.470272415	0.567367993	0.03201153	0.7	0.089890646
2006	7	1.199266667	0.5037871	0.604175076	0.046537166	0.7	0.116804602

Date	Time (t)	Hard Currency Value (HCV)	Discounted Rate $(1+r)^{-t}$	Present Value (PV)	Variance Of PV	Assigned Weight (W)	HPV (weighted PV RESULT)
2006	7	1.2008	0.519736981	0.624100166	0.055530845	0.7	0.132182511
2006	7	1.2134	0.525404879	0.63752628	0.062038829	0.7	0.142844825
2006	7	1.2582	0.52373055	0.658957778	0.073174279	0.7	0.160343337
2006	7	1.259	0.506674256	0.637902888	0.062226579	0.7	0.143147295
2006	7	1.2598	0.52373055	0.659795747	0.073628335	0.7	0.161039156
2006	7	1.279	0.504106965	0.644752808	0.06569096	0.7	0.14868047
2006	7	1.279066667	0.501872775	0.641928738	0.064251303	0.7	0.146392005
2006	7	1.273933333	0.497123226	0.633301848	0.059952266	0.7	0.139464579
2006	7	1.291233333	0.500283853	0.645983188	0.066323172	0.7	0.149680665
2006	7	1.3184	0.526412412	0.694022124	0.093374121	0.7	0.190177253
2007	8	0.9748	0.469914083	0.458072248	0.004847211	0.7	0.023979735
2007	8	1.316466667	0.476811783	0.627706819	0.057243667	0.7	0.135023454
2007	8	1.320866667	0.480303296	0.634416613	0.060499412	0.7	0.140354328
2007	8	1.3396	0.480303296	0.643414295	0.065006623	0.7	0.147594551
2007	8	1.438266667	0.480303296	0.69080422	0.091417875	0.7	0.187379374
2007	8	1.339366667	0.480303296	0.643302224	0.064949488	0.7	0.147503733
2007	8	1.366033333	0.476811783	0.65134079	0.069111393	0.7	0.154058177
2007	8	1.365766667	0.473348799	0.646484011	0.06658138	0.7	0.15008834
2007	8	1.379466667	0.473348799	0.652968889	0.069970068	0.7	0.15539556
2007	8	1.408833333	0.458111522	0.645402783	0.066024563	0.7	0.149208606
2007	8	1.4589	0.446641408	0.651605151	0.069250459	0.7	0.154275109
2007	8	1.448233333	0.446641408	0.646840976	0.066765725	0.7	0.150379107
2008	9	1.4431	0.397321582	0.573374776	0.03419705	0.7	0.094143871
2008	9	1.4477	0.397321582	0.575202455	0.034876356	0.7	0.095449085
2008	9	1.4893	0.387769342	0.577504881	0.035741623	0.7	0.097100617
2008	9	1.493333333	0.36645763	0.547243393	0.025215237	0.7	0.076061369
2008	9	1.507066667	0.307507943	0.46343497	0.005622696	0.7	0.026604876
2008	9	1.541866667	0.256910922	0.396122387	5.88604E-05	0.7	0.001093645
2008	9	1.6037	0.196738175	0.315509011	0.005320436	0.7	0.025595467
2008	9	1.6172	0.138145779	0.223409353	0.027238524	0.7	0.080283907
2008	9	1.600233333	0.138145779	0.22106548	0.028017688	0.7	0.081884671
2008	9	1.550333333	0.137151929	0.212631207	0.030912364	0.7	0.087718775
2008	9	1.496066667	0.137151929	0.205188429	0.033584925	0.7	0.09296106
2008	9	1.548666667	0.137151929	0.21240262	0.030992796	0.7	0.08787848
2009	10	1.588466667	0.109985508	0.174708313	0.04568565	0.7	0.115304396
2009	10	1.6439	0.109985508	0.180805177	0.04311651	0.7	0.110726232
2009	10	1.699833333	0.086038229	0.146250649	0.058660686	0.7	0.137354532
2009	10	1.758933333	0.101542353	0.17860623	0.044034547	0.7	0.112371317
2009	10	1.825966667	0.101542353	0.185412952	0.041224177	0.7	0.107301649

Date	Time (t)	Hard Currency Value (HCV)	Discounted Rate $(1+r)^{-t}$	Present Value (PV)	Variance Of PV	Assigned Weight (W)	HPV (weighted PV RESULT)
2009	10	1.942766667	0.100738061	0.195710548	0.037148624	0.7	0.099760797
2009	10	1.978566667	0.099940772	0.19773948	0.036370629	0.7	0.098293673
2009	10	1.9888	0.099940772	0.198762207	0.035981584	0.7	0.097556497
2009	10	1.990266667	0.099940772	0.198908787	0.035925997	0.7	0.097450973
2009	10	1.9774	0.100738061	0.199199443	0.035815899	0.7	0.097241824
2009	10	1.982633333	0.108236968	0.21459422	0.030225947	0.7	0.086350716
2009	10	1.948233333	0.131413237	0.256023649	0.017536826	0.7	0.058988275
2010	11	1.920566667	0.14893597	0.28604146	0.010487577	0.7	0.041159738
2010	11	1.875	0.174500338	0.327188133	0.003753057	0.7	0.020047933
2010	11	1.830933333	0.223341386	0.408923188	0.000419138	0.7	0.004321652
2010	11	1.8283	0.250758925	0.458462543	0.00490171	0.7	0.024168145
2010	11	1.7898	0.263248945	0.471162961	0.006841379	0.7	0.030521127
2010	11	1.7404	0.25320425	0.440676677	0.002727591	0.7	0.016034134
2010	11	1.807833333	0.268433629	0.485283263	0.009376617	0.7	0.038056804
2010	11	1.837833333	0.268433629	0.493336272	0.011001061	0.7	0.042560252
2010	11	1.835733333	0.273729909	0.502495118	0.013006214	0.7	0.047852609
2010	11	1.920533333	0.276420709	0.530875185	0.020284839	0.7	0.065316078
2010	11	1.896733333	0.278321433	0.52790154	0.01944664	0.7	0.063414884
2010	11	1.872866667	0.280511166	0.525360013	0.018744261	0.7	0.061802754
2011	12	1.919066667	0.252585645	0.484728692	0.009269523	0.7	0.037752017
2011	12	1.985533333	0.253397853	0.503129884	0.0131514	0.7	0.048225905
2011	12	2.0109	0.253669217	0.510103429	0.014799476	0.7	0.052380839
2011	12	2.0358	0.244616914	0.497991113	0.011999183	0.7	0.045227874
2011	12	2.038266667	0.272294661	0.555009131	0.027741834	0.7	0.081319485
2011	12	2.034266667	0.299469284	0.609200383	0.048730586	0.7	0.120631615
2011	12	2.024533333	0.31176034	0.631169199	0.058912449	0.7	0.137766922
2011	12	2.0459	0.341366936	0.698402615	0.096070419	0.7	0.194004936
2011	12	2.028566667	0.33987231	0.689453639	0.090602992	0.7	0.186208618
2011	12	2.0536	0.350485566	0.719757159	0.109764215	0.7	0.212972016
2011	12	2.072033333	0.331777577	0.687454198	0.089403313	0.7	0.184479252
2011	12	2.061533333	0.296238214	0.610704953	0.049397117	0.7	0.121784249
2012	13	2.103766667	0.262081239	0.551357774	0.026538835	0.7	0.078834679
2012	13	2.176166667	0.247476622	0.538550376	0.022530024	0.7	0.070296411
2012	13	2.186033333	0.221341663	0.483860253	0.009103053	0.7	0.037276142
2012	13	2.2858	0.182693406	0.417600587	0.000849737	0.7	0.007087602
2012	13	2.3538	0.131052561	0.308471519	0.00639661	0.7	0.029118227
2012	13	2.398666667	0.071944372	0.172570568	0.046604072	0.7	0.116922126
2012	13	2.420166667	0.068884722	0.166712508	0.049167662	0.7	0.121387982
2012	13	2.439166667	0.068884722	0.168021317	0.04858895	0.7	0.120386076

Date	Time (t)	Hard Currency Value (HCV)	Discounted Rate $(1+r)^{-t}$	Present Value (PV)	Variance Of PV	Assigned Weight (W)	HPV (weighted PV RESULT)
2012	13	2.4593	0.067585991	0.166214228	0.049388885	0.7	0.121770042
2012	13	2.464233333	0.067158961	0.16549535	0.049708923	0.7	0.122321852
2012	13	2.431066667	0.072712623	0.176769233	0.044808887	0.7	0.113750918
2012	13	2.462733333	0.068521289	0.168749662	0.048268384	0.7	0.119829551
2013	14	2.4791	0.055753693	0.13821898	0.062615729	0.7	0.143773355
2013	14	2.461633333	0.055122441	0.135691238	0.063887159	0.7	0.145810736
2013	14	2.4277	0.056008359	0.135971492	0.063745564	0.7	0.145584445
2013	14	2.459966667	0.055311009	0.136063237	0.063699245	0.7	0.145510388
2013	14	2.4937	0.054934562	0.136990317	0.063232139	0.7	0.144762645
2013	14	2.5492	0.054747369	0.139561992	0.061945405	0.7	0.142694216
2013	14	2.5859	0.054685123	0.141410259	0.061028797	0.7	0.141212898
2013	14	2.6274	0.056008359	0.147156362	0.058222779	0.7	0.136635973
2013	14	2.685	0.064778365	0.17392991	0.046019011	0.7	0.115892704
2013	14	2.757966667	0.075298557	0.207670909	0.032681199	0.7	0.091202887
2013	14	2.836166667	0.085231942	0.241731993	0.02152627	0.7	0.068089188
2013	14	2.906533333	0.089654033	0.260582436	0.016350199	0.7	0.056165046
2014	15	3.104766667	0.069448416	0.215621127	0.029869934	0.7	0.0856375
2014	15	3.284166667	0.061899183	0.203287233	0.034285373	0.7	0.094314011
2014	15	3.475133333	0.045420492	0.157842267	0.053180079	0.7	0.128240229
2014	15	3.699666667	0.039497866	0.14612894	0.058719656	0.7	0.137451174
2014	15	3.877466667	0.03935485	0.152597121	0.055626737	0.7	0.132342248
2014	15	3.710966667	0.039264559	0.145709471	0.058923125	0.7	0.137784397
2014	15	4.032	0.036695749	0.14795726	0.057836917	0.7	0.136001467
2014	15	4.085866667	0.035142178	0.143586253	0.059958416	0.7	0.139474595
2014	15	4.1713	0.033779611	0.140904892	0.061278744	0.7	0.141617492
2014	15	4.1298	0.032434516	0.133948064	0.064771404	0.7	0.14722051
2014	15	4.0779	0.032241576	0.131477924	0.066034818	0.7	0.149224828
2014	15	3.899933333	0.032011664	0.124843354	0.069488638	0.7	0.154646345
2015	16	3.954433333	0.025319368	0.100123751	0.083132216	0.7	0.175322862
2015	16	4.110966667	0.026005151	0.106906308	0.079267037	0.7	0.169576176
2015	16	4.2852	0.026238109	0.112435543	0.076184163	0.7	0.164932138
2015	16	4.540033333	0.027506849	0.124882012	0.069468259	0.7	0.154614596
2015	16	4.815933333	0.027825214	0.134004378	0.064742743	0.7	0.147174906
2015	16	5.130966667	0.027542031	0.141317243	0.061074763	0.7	0.141287341
2015	16	4.244366667	0.027436628	0.11645111	0.073983576	0.7	0.161582649
2015	16	4.723666667	0.027366598	0.129270686	0.067174088	0.7	0.151022358
2015	16	4.605566667	0.027157644	0.125076338	0.06936586	0.7	0.154455025
2015	16	4.526866667	0.02698481	0.122156637	0.070912331	0.7	0.15685748
2015	16	4.542166667	0.030011685	0.136318074	0.063570675	0.7	0.145304737

Date	Time (t)	Hard Currency Value (HCV)	Discounted Rate $(1+r)^{-t}$	Present Value (PV)	Variance Of PV	Assigned Weight (W)	HPV (weighted PV RESULT)
2015	16	4.537133333	0.035870901	0.162751063	0.05094016	0.7	0.124434902
2016	17	4.476933333	0.030749462	0.13766329	0.06289414	0.7	0.144220543
2016	17	4.566966667	0.031006146	0.141604036	0.060933093	0.7	0.141057849
2016	17	4.5333	0.031221783	0.141537708	0.060965843	0.7	0.141110915
2016	17	4.5387	0.030579589	0.138791583	0.06232949	0.7	0.143312972
2016	17	4.5541	0.030495026	0.138877399	0.062286648	0.7	0.143244011
2016	17	4.593966667	0.030452837	0.13989932	0.061777605	0.7	0.14242353
2016	17	4.488633333	0.030579589	0.137260565	0.063096299	0.7	0.144544882
2016	17	4.5127	0.030579589	0.137996513	0.062727115	0.7	0.143952336
2016	17	4.531533333	0.030159241	0.136667606	0.06339454	0.7	0.145022804
2016	17	4.4168	0.030621964	0.135251091	0.064109855	0.7	0.146166334
2016	17	4.397266667	0.03986439	0.175294354	0.04543547	0.7	0.114862039
2016	17	4.5119	0.071258979	0.321513386	0.004480555	0.7	0.022695107

Supplementary A2 Currency Gains now not Utilized or Invested (Yearly Analysis)

Date	Time (t)	Hard Currency Value (HCV)	Discounted Rate $(1+r)^{-t}$	Present Value (PV)	Variance Of PV	Assigned Weight (W)	HPV (weighted PV RESULT)
2000	1	0.776833333	0.704274949	0.547104256	0.014068682	0.7	0.050556546
2001	2	0.809466667	0.601484816	0.486881909	0.00340929	0.7	0.018744109
2002	3	1.0012	0.492831982	0.49342338	0.004215983	0.7	0.021748493
2003	4	1.156033333	0.488737071	0.564996345	0.018633223	0.7	0.061546249
2004	5	1.286033333	0.454166947	0.584073833	0.024205464	0.7	0.073916159
2005	6	1.1928	0.523225444	0.624103309	0.038263479	0.7	0.101847206
2006	7	1.3184	0.526412412	0.694022124	0.070505833	0.7	0.156227517
2007	8	1.448233333	0.446641408	0.646840976	0.047675934	0.7	0.118798088
2008	9	1.548666667	0.137151929	0.21240262	0.046694957	0.7	0.117081692
2009	10	1.948233333	0.131413237	0.256023649	0.029745601	0.7	0.08538782
2010	11	1.872866667	0.280511166	0.525360013	0.009383261	0.7	0.038075678
2011	12	2.061533333	0.296238214	0.610704953	0.033201276	0.7	0.092216437
2012	13	2.462733333	0.068521289	0.168749662	0.067466488	0.7	0.151482223
2013	14	2.906533333	0.089654033	0.260582436	0.028193884	0.7	0.082244797
2014	15	3.899933333	0.032011664	0.124843354	0.092202974	0.7	0.188504383
2015	16	4.537133333	0.035870901	0.162751063	0.070618661	0.7	0.156402479
2016	17	4.5119	0.071258979	0.321513386	0.011444591	0.7	0.043754245

Supplementary B1 Risk of Future Currency Value Depreciation not Avoided (Monthly Analysis)

Date	Time (t)	Hard Currency Value	Future Value rate $(1+r)^t$	Future Value (FV)	VARIANCE Of FV	Assigned Weights (W)	HFV (Weighted FV RESULT)
2000	1	0.5865	1.3419	0.7870244	904.0023165	0.2	3.901520639
2000	1	0.4503	1.3419	0.6042576	915.0260829	0.2	3.910989903
2000	1	0.481567	1.3419	0.6462143	912.4895111	0.2	3.90881914
2000	1	0.528167	1.3426	0.7091166	908.6932353	0.2	3.905561303
2000	1	0.6749	1.3529	0.9130722	896.4385355	0.2	3.894969879
2000	1	0.6104	1.4382	0.8778773	898.5472876	0.2	3.896800637
2000	1	0.698933	1.4568	1.0182061	890.1540498	0.2	3.88949336
2000	1	0.731267	1.4321	1.047247	888.4219958	0.2	3.887978552
2000	1	0.7347	1.416	1.0403352	888.834075	0.2	3.888339159
2000	1	0.749433	1.4193	1.0636707	887.4432003	0.2	3.887121479
2000	1	0.7602	1.4199	1.079408	886.5058231	0.2	3.886299964
2000	1	0.776833	1.4199	1.1030257	885.0999842	0.2	3.885066586
2001	2	0.7943	2.01611601	1.6014009	855.694404	0.2	3.858901902
2001	2	0.807067	2.01611601	1.62714	854.1892142	0.2	3.857543363
2001	2	0.8028	2.093809	1.6809099	851.0490954	0.2	3.854703009
2001	2	0.802233	2.11644304	1.6978812	850.0591852	0.2	3.853805861
2001	2	0.8001	2.14827649	1.718836	848.8377137	0.2	3.852697698
2001	2	0.788433	2.1609	1.7037256	849.7184213	0.2	3.853496836
2001	2	0.783	2.12226624	1.6617345	852.1682612	0.2	3.855716296
2001	2	0.793333	1.98443569	1.574319	857.2795546	0.2	3.860330545
2001	2	0.799833	1.87991521	1.5036188	861.424659	0.2	3.864056428
2001	2	0.804833	1.80848704	1.4555307	864.2497521	0.2	3.86658759
2001	2	0.803133	1.739761	1.3972601	867.6792405	0.2	3.869651385
2001	2	0.809467	1.66255236	1.3457807	870.7146818	0.2	3.872355079
2002	3	0.812867	2.025244912	1.6462541	853.0723052	0.2	3.856534036
2002	3	0.820833	1.874516337	1.5386655	859.3686467	0.2	3.862210151
2002	3	0.827733	1.888232256	1.5629528	857.9452734	0.2	3.860929906
2002	3	0.8546	1.897413272	1.6215294	854.5172049	0.2	3.85783956
2002	3	0.880133	1.897413272	1.6699767	851.6871177	0.2	3.855280802
2002	3	0.903433	1.948441249	1.7602868	846.4241114	0.2	3.850504234
2002	3	1.0094	1.957816251	1.9762197	833.9063045	0.2	3.839047198
2002	3	0.9584	2.005142581	1.9217286	837.0563988	0.2	3.841943238
2002	3	0.9625	2.000376	1.9253619	836.8461781	0.2	3.841750243
2002	3	0.969767	2.005142581	1.9445204	835.7380976	0.2	3.84073232
2002	3	0.9813	2.005142581	1.9676464	834.4015283	0.2	3.839503061
2002	3	1.0012	2.029089096	2.031524	830.7152715	0.2	3.836104584
2003	4	1.0642	2.585098015	2.7510613	789.7558076	0.2	3.797506848

Date	Time (t)	Hard Currency Value	Future Value rate $(1+r)^t$	Future Value (FV)	VARIANCE Of FV	Assigned Weights (W)	HFV (Weighted FV RESULT)
2003	4	1.0524	2.634375702	2.772417	788.5559636	0.2	3.796352266
2003	4	1.086333	2.760652033	2.9989883	775.8824906	0.2	3.784070249
2003	4	1.087667	3.12900721	3.4033168	753.5210995	0.2	3.762002532
2003	4	1.0855	3.176325951	3.4479018	751.0753419	0.2	3.759557235
2003	4	1.099633	3.351129311	3.6850135	738.1351154	0.2	3.746512391
2003	4	1.094767	3.765342322	4.1221713	714.5722565	0.2	3.722281639
2003	4	1.0819	2.76922881	2.9960286	776.0473809	0.2	3.784231073
2003	4	1.080067	2.659277071	2.8721965	782.9620538	0.2	3.790950755
2003	4	1.111167	2.634375702	2.9272305	779.8852223	0.2	3.787966576
2003	4	1.122933	2.348997631	2.6377677	796.1363294	0.2	3.803623206
2003	4	1.156033	2.046089933	2.3653482	811.5836621	0.2	3.81827021
2004	5	1.2046	2.155225675	2.5961848	798.484655	0.2	3.805864437
2004	5	1.221767	2.268435589	2.771499	788.6075216	0.2	3.796401908
2004	5	1.215767	2.316987673	2.8169164	786.0587478	0.2	3.793944741
2004	5	1.205833	2.287757757	2.7586546	789.329085	0.2	3.797096383
2004	5	1.198833	2.183094602	2.6171666	797.2993139	0.2	3.804733813
2004	5	1.216867	2.183094602	2.6565351	795.0776089	0.2	3.802611039
2004	5	1.225333	2.183094602	2.6750186	794.0355856	0.2	3.801613781
2004	5	1.219033	2.192448036	2.6726672	794.1681068	0.2	3.801740667
2004	5	1.2107	2.192448036	2.6543968	795.1981964	0.2	3.802726378
2004	5	1.220867	2.201833502	2.6881451	793.2959818	0.2	3.800905315
2004	5	1.2574	2.201833502	2.7685854	788.7711673	0.2	3.796559455
2004	5	1.286033	2.201833502	2.8316313	785.2338488	0.2	3.793148124
2005	6	1.2635	2.57834703	3.2577415	761.5344781	0.2	3.769970172
2005	6	1.258433	2.591586268	3.2613385	761.335962	0.2	3.769773601
2005	6	1.2774	2.591586268	3.3104923	758.6258463	0.2	3.767085931
2005	6	1.263867	2.604882108	3.2922237	759.6325318	0.2	3.768085174
2005	6	1.251067	2.604882108	3.2588812	761.4715772	0.2	3.769907892
2005	6	1.223867	2.461709311	3.012804	775.1130212	0.2	3.783319393
2005	6	1.199267	2.386420684	2.8619548	783.5353165	0.2	3.791505718
2005	6	1.2164	2.289029177	2.7843751	787.88451	0.2	3.795705529
2005	6	1.2212	2.17196875	2.6524082	795.3103545	0.2	3.802833643
2005	6	1.200433	2.049007256	2.4596966	806.2169088	0.2	3.81320699
2005	6	1.176233	2.005758021	2.3592394	811.9317538	0.2	3.818597688
2005	6	1.1928	1.91122204	2.2797056	816.4706175	0.2	3.822857521
2006	7	1.206467	2.126427083	2.5654634	800.2218199	0.2	3.807518988
2006	7	1.199267	1.984965474	2.3805029	810.7204248	0.2	3.817457607
2006	7	1.2008	1.92405012	2.3103994	814.7174771	0.2	3.821214408
2006	7	1.2134	1.903294089	2.309457	814.7712727	0.2	3.821264869

Date	Time (t)	Hard Currency Value	Future Value rate $(1+r)^t$	Future Value (FV)	VARIANCE Of FV	Assigned Weights (W)	HFV (Weighted FV RESULT)
2006	7	1.2582	1.909378783	2.4023804	809.475063	0.2	3.816284073
2006	7	1.259	1.973654648	2.4848312	804.7901994	0.2	3.811856437
2006	7	1.2598	1.909378783	2.4054354	809.3012347	0.2	3.816120156
2006	7	1.279	1.983705978	2.5371599	801.8239278	0.2	3.80904236
2006	7	1.279067	1.992536852	2.5485875	801.1768835	0.2	3.808427408
2006	7	1.273933	2.011573687	2.5626108	800.3832191	0.2	3.807672566
2006	7	1.291233	1.998865231	2.5810014	799.3429763	0.2	3.806682299
2006	7	1.3184	1.899651256	2.5045002	803.6746127	0.2	3.810799064
2007	8	0.9748	2.128048587	2.0744218	828.244302	0.2	3.833819759
2007	8	1.316467	2.097263606	2.7609776	789.1985572	0.2	3.796970793
2007	8	1.320867	2.082017776	2.7500679	789.8116444	0.2	3.797560544
2007	8	1.3396	2.082017776	2.789071	787.6209097	0.2	3.795451511
2007	8	1.438267	2.082017776	2.9944968	776.1327325	0.2	3.78431431
2007	8	1.339367	2.082017776	2.7885852	787.6481777	0.2	3.795477791
2007	8	1.366033	2.097263606	2.864932	783.3686507	0.2	3.791344406
2007	8	1.365767	2.112607031	2.8853283	782.2273357	0.2	3.790239015
2007	8	1.379467	2.112607031	2.914271	780.6092146	0.2	3.788669613
2007	8	1.408833	2.182874588	3.0753065	771.6366813	0.2	3.779919692
2007	8	1.4589	2.238932578	3.2663787	761.0578462	0.2	3.769498142
2007	8	1.448233	2.238932578	3.2424968	762.3760924	0.2	3.770803085
2008	9	1.4431	2.516852958	3.6320705	741.0146967	0.2	3.749430987
2008	9	1.4477	2.516852958	3.643648	740.3845137	0.2	3.748793043
2008	9	1.4893	2.578852663	3.8406853	729.7005675	0.2	3.73791083
2008	9	1.493333	2.728828436	4.0750505	717.0936946	0.2	3.724904827
2008	9	1.507067	3.251948521	4.9009032	673.5453577	0.2	3.678522099
2008	9	1.541867	3.892399719	6.0015614	617.6265886	0.2	3.615307123
2008	9	1.6037	5.082897623	8.1514429	515.390462	0.2	3.486801912
2008	9	1.6172	7.238730048	11.706474	366.6145554	0.2	3.257181359
2008	9	1.600233	7.238730048	11.583657	371.3328428	0.2	3.26552244
2008	9	1.550333	7.291184388	11.303766	382.1981769	0.2	3.284412696
2008	9	1.496067	7.291184388	10.908098	397.8252722	0.2	3.310842136
2008	9	1.548667	7.291184388	11.291614	382.673464	0.2	3.285229164
2009	10	1.588467	9.092106932	14.442509	269.3257406	0.2	3.062355269
2009	10	1.6439	9.092106932	14.946515	253.0371362	0.2	3.024383449
2009	10	1.699833	11.62274041	19.756722	123.1419453	0.2	2.618672587
2009	10	1.758933	9.848107415	17.322164	183.1012548	0.2	2.834903396
2009	10	1.825967	9.848107415	17.982316	165.6713872	0.2	2.778750167
2009	10	1.942767	9.926734607	19.285329	133.8261786	0.2	2.662614026
2009	10	1.978567	10.00592633	19.797392	122.2409586	0.2	2.61482934

Date	Time (t)	Hard Currency Value	Future Value rate $(1+r)^t$	Future Value (FV)	VARIANCE Of FV	Assigned Weights (W)	HFV (Weighted FV RESULT)
2009	10	1.9888	10.00592633	19.899786	119.9872535	0.2	2.605115738
2009	10	1.990267	10.00592633	19.914462	119.665965	0.2	2.603719103
2009	10	1.9774	9.926734607	19.629125	125.9900876	0.2	2.630675472
2009	10	1.982633	9.23898759	18.317525	157.1545771	0.2	2.749573965
2009	10	1.948233	7.609583502	14.825244	256.909972	0.2	3.033585168
2010	11	1.920567	6.714294726	12.895251	322.5043097	0.2	3.174732256
2010	11	1.875	5.730647939	10.744965	404.3594434	0.2	3.321647314
2010	11	1.830933	4.477450507	8.1979134	513.2826568	0.2	3.483945222
2010	11	1.8283	3.987893946	7.2910665	555.1956064	0.2	3.539070419
2010	11	1.7898	3.798685694	6.7988877	578.6318623	0.2	3.568457008
2010	11	1.7404	3.949380782	6.8735023	575.0477531	0.2	3.564025332
2010	11	1.807833	3.725315647	6.7347498	581.7216182	0.2	3.572259836
2010	11	1.837833	3.725315647	6.8465093	576.3430762	0.2	3.565629515
2010	11	1.835733	3.653236154	6.7063674	583.0915297	0.2	3.573940735
2010	11	1.920533	3.617673958	6.9478634	571.4869009	0.2	3.559600475
2010	11	1.896733	3.592967987	6.8149021	577.861669	0.2	3.567506537
2010	11	1.872867	3.564920477	6.6766207	584.5290165	0.2	3.575701156
2011	12	1.919067	3.959053176	7.597687	540.8400777	0.2	3.520576399
2011	12	1.985533	3.946363349	7.835636	529.8292288	0.2	3.506123282
2011	12	2.0109	3.942141699	7.9272527	525.6199493	0.2	3.500534546
2011	12	2.0358	4.088024757	8.3224008	507.6574441	0.2	3.476275213
2011	12	2.038267	3.67249213	7.4855183	546.0698426	0.2	3.527358805
2011	12	2.034267	3.339240623	6.7929059	578.9196779	0.2	3.568811933
2011	12	2.024533	3.207592093	6.4938771	593.3988094	0.2	3.586487604
2011	12	2.0459	2.929399111	5.9932576	618.0393882	0.2	3.615790262
2011	12	2.028567	2.942281468	5.9686141	619.2652914	0.2	3.617223535
2011	12	2.0536	2.853184542	5.8592998	624.7178245	0.2	3.623571035
2011	12	2.072033	3.014067467	6.2452483	605.5737133	0.2	3.601085252
2011	12	2.061533	3.375661724	6.9590392	570.9526959	0.2	3.558934749
2012	13	2.103767	3.815610778	8.0271548	521.0491416	0.2	3.494425106
2012	13	2.176167	4.040785719	8.7934232	486.6538553	0.2	3.447021829
2012	13	2.186033	4.517902262	9.8762849	440.050081	0.2	3.378317175
2012	13	2.2858	5.473651305	12.511672	336.4283578	0.2	3.201684394
2012	13	2.3538	7.630526183	17.960733	166.2274661	0.2	2.780613052
2012	13	2.398667	13.89962783	33.340574	6.184762791	0.2	1.439675485
2012	13	2.420167	14.51700715	35.133577	18.31772518	0.2	1.788851576
2012	13	2.439167	14.51700715	35.4094	20.75480614	0.2	1.834103063
2012	13	2.4593	14.79596557	36.387718	30.62584776	0.2	1.982520213
2012	13	2.464233	14.89004574	36.692547	34.09265307	0.2	2.025499591

Date	Time (t)	Hard Currency Value	Future Value rate $(1+r)^t$	Future Value (FV)	VARIANCE Of FV	Assigned Weights (W)	HFV (Weighted FV RESULT)
2012	13	2.431067	13.75277032	33.433902	6.657668768	0.2	1.461047841
2012	13	2.462733	14.59400456	35.941141	25.88251237	0.2	1.916908071
2013	14	2.4791	17.9360316	44.465216	185.2745741	0.2	2.841601444
2013	14	2.461633	18.14143179	44.657553	190.5475882	0.2	2.857595091
2013	14	2.4277	17.85447785	43.345316	156.0415755	0.2	2.745668273
2013	14	2.459967	18.07958355	44.475173	185.545732	0.2	2.84243272
2013	14	2.4937	18.20347644	45.394009	211.4218821	0.2	2.917628462
2013	14	2.5492	18.26571809	46.562969	246.7825126	0.2	3.009281923
2013	14	2.5859	18.28650918	47.287084	270.057571	0.2	3.064017712
2013	14	2.6274	17.85447785	46.910855	257.8336546	0.2	3.035763401
2013	14	2.685	15.43725288	41.449024	112.2618305	0.2	2.57067056
2013	14	2.757967	13.28046704	36.627085	33.33249154	0.2	2.016385453
2013	14	2.836167	11.73269052	33.275866	5.867101999	0.2	1.424573062
2013	14	2.906533	11.1539879	32.419438	2.451673278	0.2	1.196444393
2014	15	3.104767	14.39917653	44.706083	191.8897531	0.2	2.86160942
2014	15	3.284167	16.15530214	53.056705	492.9753922	0.2	3.455930883
2014	15	3.475133	22.01649411	76.510253	2084.524843	0.2	4.611066813
2014	15	3.699667	25.31782324	93.667507	3945.579889	0.2	5.238683588
2014	15	3.877467	25.40982847	98.525763	4579.514105	0.2	5.397142442
2014	15	3.710967	25.46825976	94.511863	4052.367368	0.2	5.266738576
2014	15	4.032	27.25111287	109.87649	6244.607895	0.2	5.742500409
2014	15	4.085867	28.45583459	116.26675	7295.396009	0.2	5.923928039
2014	15	4.1713	29.60365644	123.48573	8580.701594	0.2	6.11933966
2014	15	4.1298	30.83135277	127.32732	9307.168048	0.2	6.219615126
2014	15	4.0779	31.01585326	126.47955	9144.311294	0.2	6.197695006
2014	15	3.899933	31.238614	121.82851	8276.4245	0.2	6.075311515
2015	16	3.954433	39.49545721	156.18215	15707.23213	0.2	6.905894388
2015	16	4.110967	38.45392059	158.08279	16187.25155	0.2	6.947597067
2015	16	4.2852	38.11250311	163.3197	17547.25247	0.2	7.060603521
2015	16	4.540033	36.35458199	165.05101	18008.93101	0.2	7.097372295
2015	16	4.815933	35.93862692	173.07803	20227.77304	0.2	7.26423047
2015	16	5.130967	36.30814287	186.29587	24162.28226	0.2	7.527098351
2015	16	4.244367	36.44762734	154.69709	15337.19737	0.2	6.87304522
2015	16	4.723667	36.54089591	172.60701	20094.01404	0.2	7.254597807
2015	16	4.605567	36.82204593	169.58639	19246.77085	0.2	7.192362618
2015	16	4.526867	37.05788547	167.75611	18742.28104	0.2	7.15425621
2015	16	4.542167	33.32035524	151.34661	14518.5513	0.2	6.79805473
2015	16	4.537133	27.87774936	126.48507	9145.366627	0.2	6.197838053
2016	17	4.476933	32.52089444	145.59388	13165.31825	0.2	6.66632177

Date	Time (t)	Hard Currency Value	Future Value rate $(1+r)^t$	Future Value (FV)	VARIANCE Of FV	Assigned Weights (W)	HFV (Weighted FV RESULT)
2016	17	4.566967	32.25167016	147.2923	13557.95851	0.2	6.705618784
2016	17	4.5333	32.02892042	145.1967	13074.33293	0.2	6.657082022
2016	17	4.5387	32.70155085	148.42253	13822.43995	0.2	6.73157893
2016	17	4.5541	32.79223285	149.33911	14038.80234	0.2	6.752522063
2016	17	4.593967	32.83766256	150.85513	14400.35318	0.2	6.786949672
2016	17	4.488633	32.70155085	146.78527	13440.13949	0.2	6.693923661
2016	17	4.5127	32.70155085	147.57229	13623.23927	0.2	6.712063814
2016	17	4.531533	33.15733298	150.25356	14256.33708	0.2	6.773319954
2016	17	4.4168	32.65629841	144.23634	12855.63285	0.2	6.634660309
2016	17	4.397267	25.0850445	110.30563	6312.616235	0.2	5.754954278
2016	17	4.5119	14.0333193	63.316933	1053.8644	0.2	4.02306405

Appendix B2 Risk of Future Currency Value Depreciation not Avoided (Yearly Analysis)

Date	Time (t)	Hard Currency Value	Future Value rate $(1+r)^t$	Future Value (FV)	VARIANCE Of FV	Assigned Weights (W)	HFV (Weighted FV RESULT)
2000	1	0.776833333	1.4199	1.10302565	606.5968701	0.2	3.602301285
2001	2	0.809466667	1.66255236	1.345780717	594.6980801	0.2	3.588056782
2002	3	1.0012	2.029089096	2.031524003	561.7226595	0.2	3.547352845
2003	4	1.156033333	2.046089933	2.365348166	546.0103728	0.2	3.527281972
2004	5	1.286033333	2.201833502	2.831631277	524.4366438	0.2	3.498957005
2005	6	1.1928	1.91122204	2.279705649	550.0201016	0.2	3.532447471
2006	7	1.3184	1.899651256	2.504500215	539.5266418	0.2	3.518864784
2007	8	1.448233333	2.238932578	3.242496791	505.7873357	0.2	3.47371025
2008	9	1.548666667	7.291184388	11.29161422	208.5308934	0.2	2.909605311
2009	10	1.948233333	7.609583502	14.82524423	219.7878665	0.2	2.940361602
2010	11	1.872866667	3.564920477	6.67662073	363.1155972	0.2	3.250940199
2011	12	2.061533333	3.375661724	6.959039166	352.4320563	0.2	3.231581258
2012	13	2.462733333	14.59400456	35.94114149	104.2222206	0.2	2.532748486
2013	14	2.906533333	11.1539879	32.41943763	44.7189731	0.2	2.138446377
2014	15	3.899933333	31.238614	121.828512	9234.498688	0.2	6.209872234
2015	16	4.537133333	27.87774936	126.4850659	10151.13736	0.2	6.328531424
2016	17	4.5119	14.0333193	63.31693335	1412.611203	0.2	4.265839676

Supplementary C1 Gold Impact Factor or Cost of Holding Gold in Reserves (Monthly Analysis)

Date	Time (t)	$\left(GGP - \left(\frac{CIC}{G}\right)\right)^2$	$\text{LOG}\left(GGP_d - \left(\frac{CIC}{G}\right)\right)$	Stability Monitor, SM (Absolute Value)	$\text{SM} \times \text{LOG}\left(GGP_d - \left(\frac{CIC}{G}\right)\right)^2$	Weights (W)	$\lvert\text{SM}\rvert \times \text{LOG}\left(\left(\left(GGP_d - \left(\frac{CIC}{G}\right)\right)^2\right)^{0.1}\right)$
2000	1	221641.7	5.34565	0	0	0.1	0
2000	1	191222.0	5.28153	0.4204	2.220787095	0.1	1.083055451
2000	1	201636.2	5.304568	0.5912	3.13658773	0.1	1.121103592
2000	1	212999.4	5.328378	0.7512	4.003023197	0.1	1.148785144
2000	1	133744.8	5.126276	3.0600	15.6865181	0.1	1.316899569
2000	1	222910.1	5.3481	3.6681	19.61767222	0.1	1.346681045
2000	1	238498.9	5.377	0.75384	4.053795668	0.1	1.150233959
2000	1	250048.2	5.39802	0.88017	4.7512062	0.1	1.168639034
2000	1	254861.5	5.40630	5.0176097	27.12672481	0.1	1.391040377
2000	1	283204.7559	5.452100542	0.815349562	4.445367788	0.1	1.160889197
2000	1	393944.2362	5.595434751	0.505890297	2.830676149	0.1	1.109657668
2000	1	624700.2784	5.795671699	1.667853053	9.666328741	0.1	1.2546603
2001	2	607167.4462	5.783308478	0.887046389	5.130062902	0.1	1.177639242
2001	2	533769.2264	5.727353532	0.405055729	2.31989736	0.1	1.087794546
2001	2	478051.4054	5.679474599	3.688535158	20.94894174	0.1	1.355552084
2001	2	473956.7324	5.675738697	0.907966216	5.153378986	0.1	1.178173386
2001	2	469655.3417	5.671779266	17.64537915	100.0806956	0.1	1.58502104
2001	2	436126.132	5.63961211	0.494294639	2.78763003	0.1	1.10795855
2001	2	460909.3519	5.66361552	2.37689538	13.46182156	0.1	1.296911625
2001	2	459667.2882	5.662443599	1.061959486	6.013285695	0.1	1.196495815
2001	2	458587.3674	5.661422087	5.177085817	29.30966799	0.1	1.401848528
2001	2	530548.5671	5.724725146	0.241417081	1.382046435	0.1	1.032885697
2001	2	617482.7197	5.790624808	8.575512181	49.65757358	0.1	1.477741771
2001	2	936885.0829	5.971686324	1.183601323	7.068095834	0.1	1.215990671
2002	3	759341.5194	5.880437147	5.997286802	35.26666809	0.1	1.42802708
2002	3	646059.3132	5.810272391	7.048079443	40.9512614	0.1	1.449528399
2002	3	638171.9058	5.804937681	0.543401983	3.154414645	0.1	1.121739151
2002	3	627274.5168	5.797457645	1.794780524	10.40516407	0.1	1.263935445
2002	3	580224.5542	5.763596104	1.834494042	10.57328271	0.1	1.265962917
2002	3	611039.7406	5.786069457	1.027714314	5.946426404	0.1	1.195158776
2002	3	682703.7656	5.834232298	0.026726686	0.155929697	0.1	0.830410598
2002	3	702790.3954	5.846825818	0.002557005	0.01495036	0.1	0.65684843
2002	3	719634.3338	5.857111875	7.209165386	42.2248882	0.1	1.453974704
2002	3	1065667.6	6.027621762	0.367237537	2.213568972	0.1	1.082702915
2002	3	1515438.644	6.180538358	1.334154435	8.245792661	0.1	1.23487569

Date	Time (t)	$\left(GGP - \left(\frac{CIC}{G}\right)\right)^2$	LOG $\left(GGP_d - \left(\frac{CIC}{G}\right)\right)$	Stability Monitor, SM (Absolute Value)	SM × LOG $\left(GGP_d - \left(\frac{CIC}{G}\right)\right)^2$	Weights (W)	lSMl × LOG $\left(\left(GGP_d - \left(\frac{CIC}{G}\right)\right)^2\right)^{0.1}$
2002	3	2188292.282	6.340105329	2.947822571	18.68950559	0.1	1.340169659
2003	4	1635271.661	6.21358991	3.319349635	20.6250774	0.1	1.353441717
2003	4	1475118.398	6.16882688	8.671841968	53.49509183	0.1	1.488782975
2003	4	1294084.776	6.111962728	2.966407881	18.13057441	0.1	1.336106745
2003	4	1377319.982	6.139034848	27.83685877	170.8914461	0.1	1.672137676
2003	4	1443045.692	6.159280083	7.593011957	46.76748731	0.1	1.468907349
2003	4	1588324.143	6.200939137	4.013148719	24.88529096	0.1	1.379095281
2003	4	1465047.591	6.165851733	9.096875183	56.08998361	0.1	1.495851672
2003	4	1481782.939	6.17078459	0.734461077	4.532201095	0.1	1.163137123
2003	4	1447174.363	6.16052086	32.55688773	200.567386	0.1	1.699127745
2003	4	1852269.291	6.267704127	0.222882607	1.396962236	0.1	1.033995066
2003	4	2112378.926	6.324771826	2.523518003	15.96067557	0.1	1.319183246
2003	4	4043683.348	6.60677714	1.971033884	13.0221816	0.1	1.29261257
2004	5	3429201.955	6.535193063	0.115424441	0.754321004	0.1	0.972200007
2004	5	2821804.498	6.450526921	1.35197253	8.720935205	0.1	1.241813297
2004	5	2525173.05	6.402291146	11.7637406	75.31489225	0.1	1.540593588
2004	5	2887705.078	6.460552837	7.468489975	48.25057409	0.1	1.473500368
2004	5	3088460.97	6.489742117	2.798276082	18.16009015	0.1	1.336324098
2004	5	3078319.841	6.488313741	2.10904228	13.68412801	0.1	1.299037573
2004	5	3036137.321	6.48232141	2.997665428	19.43183078	0.1	1.345399841
2004	5	2887742.143	6.460558411	9.256941753	59.8050129	0.1	1.505475731
2004	5	2946611.229	6.469322839	0.644580661	4.170000392	0.1	1.153489404
2004	5	3445865.022	6.537298262	4.266519899	27.89151312	0.1	1.394913276
2004	5	4088317.289	6.611544594	1.718026259	11.35880723	0.1	1.275067784
2004	5	5443756.015	6.735898652	0.678436737	4.5698811	0.1	1.164100538
2005	6	4720961.404	6.67403045	2.106442879	14.05846391	0.1	1.302548152
2005	6	4093952.2	6.612142768	0.204379461	1.351386176	0.1	1.030571071
2005	6	4084873.644	6.611178627	0.511588771	3.382204751	0.1	1.12958779
2005	6	4114527.899	6.614320011	0.384506615	2.543249796	0.1	1.097839625
2005	6	4225301.637	6.625857718	0.704877197	4.670416013	0.1	1.166636491
2005	6	3960978.065	6.597802437	1.357937706	8.959404707	0.1	1.245167898
2005	6	3848258.596	6.585264248	1.220883965	8.039843527	0.1	1.231756208
2005	6	3891115.439	6.590074115	1.243278036	8.193294403	0.1	1.234087223
2005	6	4030627.004	6.60537261	15.3973176	101.70502	0.1	1.587574955
2005	6	4665822.2	6.668928185	0.735146733	4.902640767	0.1	1.172311451
2005	6	5390869.406	6.731658811	3.052057746	20.54541142	0.1	1.352918029

Date	Time (t)	$\left(GGP - \left(\frac{CIC}{G}\right)\right)^2$	$\text{LOG}\left(GGP_d - \left(\frac{CIC}{G}\right)\right)$	Stability Monitor, SM (Absolute Value)	SM $\times$ $\text{LOG}\left(GGP_d - \left(\frac{CIC}{G}\right)\right)^2$	Weights (W)	$\text{lSMl} \times \text{LOG}\left(\left(\left(GGP_d - \left(\frac{CIC}{G}\right)\right)^2\right)^{0.1}\right)$
2005	6	6605772.032	6.819923582	1.80236656	12.29200221	0.1	1.285174983
2006	7	5380496.556	6.730822358	8.87796179	59.75598371	0.1	1.505352264
2006	7	4884563.28	6.68882574	2.148709536	14.37234365	0.1	1.305427506
2006	7	4702416.234	6.672321068	10.46023218	69.79402758	0.1	1.528909665
2006	7	4826302.189	6.683614511	12.26627771	81.98307172	0.1	1.553718792
2006	7	5179112.667	6.714255359	0.944465036	6.341379431	0.1	1.202869115
2006	7	5279420.689	6.72258627	165.1895815	1110.501213	0.1	2.016284928
2006	7	5304131.731	6.724614301	121.8887789	819.6550258	0.1	1.955974149
2006	7	5366497.061	6.729690896	0.567047684	3.816055636	0.1	1.143303334
2006	7	5470355.148	6.738015523	992.4085613	6686.864291	0.1	2.412805149
2006	7	6145669.171	6.788569178	5.775375136	39.20653364	0.1	1.443230986
2006	7	5605060.019	6.748580267	4.626358406	31.22135105	0.1	1.410734117
2006	7	10938621.77	7.038962606	1.008555601	7.099185163	0.1	1.216524474
2007	8	8214381.749	6.914574882	0.090368323	0.624858535	0.1	0.954065454
2007	8	7128556.165	6.853001576	0.183382857	1.256723009	0.1	1.023113833
2007	8	7304376.707	6.863583163	5.780799264	39.6769965	0.1	1.444953525
2007	8	7127610.268	6.852943945	2.514029261	17.2285016	0.1	1.329305344
2007	8	7316237.551	6.864287798	0.544731818	3.739195971	0.1	1.140979451
2007	8	7027191.46	6.846781786	0.13025959	0.891858988	0.1	0.988620517
2007	8	12384379.87	7.092874265	1.248547946	8.855793595	0.1	1.243720373
2007	8	9806070.272	6.991495001	1.094374656	7.651314933	0.1	1.225670146
2007	8	10945553.28	7.039237719	10.41189351	73.29179352	0.1	1.536404374
2007	8	13374017.84	7.126261898	3.330856334	23.73655458	0.1	1.372592963
2007	8	13443528.24	7.128513264	1.753995145	12.50337765	0.1	1.287368075
2007	8	19617696.18	7.292648004	4.56421892	33.285242	0.1	1.419793462
2008	9	13945092.78	7.144421408	29.15718351	208.3112061	0.1	1.705576735
2008	9	12517908.42	7.09753177	11.75650123	83.44214097	0.1	1.556462078
2008	9	13310715.26	7.124201393	0.067317386	0.479582614	0.1	0.929151095
2008	9	13037836.81	7.115205541	18.42883179	131.1249261	0.1	1.628427866
2008	9	13393875.41	7.126906255	0.856379538	6.103336689	0.1	1.198275646
2008	9	13317477.38	7.124421968	2.1524776	15.3351587	0.1	1.31391971
2008	9	13604612.86	7.133686188	0.478922544	3.416483135	0.1	1.130727431
2008	9	14384535.45	7.157895841	5.231358632	37.4455202	0.1	1.436613644
2008	9	15561593.48	7.192054066	10.19524621	73.32476192	0.1	1.536473471
2008	9	20174392.09	7.304800457	4.455348492	32.5454317	0.1	1.416605761
2008	9	22020109.5	7.342819474	2.140360964	15.71628417	0.1	1.317149245

Date	Time (t)	$\left(GGP - \left(\frac{CIC}{G}\right)\right)^2$	$\text{LOG}\left(GGP_d - \left(\frac{CIC}{G}\right)\right)$	Stability Monitor, SM (Absolute Value)	$SM \times \text{LOG}\left(GGP_d - \left(\frac{CIC}{G}\right)\right)^2$	Weights (W)	$\lvert SM \rvert \times \text{LOG}\left(\left(GGP_d - \left(\frac{CIC}{G}\right)\right)^2\right)^{0.1}$
2008	9	34327139.25	7.535637612	3.581308235	26.98744103	0.1	1.390324483
2009	10	25491554.55	7.406396321	2.180885745	16.15250416	0.1	1.320760239
2009	10	19619264.48	7.292682722	5.012008351	36.5509867	0.1	1.433144266
2009	10	19342904.78	7.286521694	0.639219674	4.657688024	0.1	1.166318165
2009	10	20688802.54	7.315735355	0.531824975	3.890690775	0.1	1.145519988
2009	10	18944757.06	7.27748904	2.561497319	18.64126866	0.1	1.339823363
2009	10	18628610.65	7.270180466	0.006042372	0.043929132	0.1	0.731602637
2009	10	19611318.22	7.292506787	1.646286089	12.00555248	0.1	1.282148165
2009	10	20533877.49	7.312470967	1.82857571	13.37140679	0.1	1.296037925
2009	10	21603589.36	7.334525914	58.80101147	431.2775424	0.1	1.834322463
2009	10	30625112.98	7.486077699	6.494673061	48.61962717	0.1	1.47462354
2009	10	35586259.97	7.551282347	43.35321186	327.3723434	0.1	1.78444924
2009	10	45633461.78	7.659283416	3.435100366	26.31040727	0.1	1.386796564
2010	11	37379455.22	7.572632968	0.786009701	5.952162974	0.1	1.195274024
2010	11	32947616.06	7.517823997	0.51246616	3.852630392	0.1	1.144394428
2010	11	33468597.76	7.524637517	0.782542418	5.888348037	0.1	1.19398631
2010	11	33068284.03	7.519411659	29.78162778	223.9403192	0.1	1.717960718
2010	11	31999502.44	7.505143226	4.819057969	36.16772027	0.1	1.431634361
2010	11	30757642.24	7.487953041	0.385514041	2.886711032	0.1	1.111834974
2010	11	33973154.24	7.531135871	1.086103037	8.179589542	0.1	1.233880643
2010	11	34907206.13	7.542915091	2.710751198	20.44696612	0.1	1.352268363
2010	11	41416991.33	7.617178547	8.804204447	67.06319724	0.1	1.522819486
2010	11	121957831.1	8.086209692	3.210772156	25.96297693	0.1	1.384954318
2010	11	68497456.56	7.835674446	3.638205654	28.50779507	0.1	1.397965207
2010	11	91997433.49	7.963775712	2.295109774	18.27773947	0.1	1.337187316
2011	12	78562423.4	7.895214872	0.124158386	0.980257136	0.1	0.998007951
2011	12	66922388.85	7.825571435	0.638052289	4.993123766	0.1	1.174457304
2011	12	66978896.96	7.825937991	2.900682545	22.70056173	0.1	1.366481198
2011	12	75641116.1	7.878757928	3.354897913	26.43242853	0.1	1.387438389
2011	12	76451959.31	7.88338862	19.33934602	152.4595803	0.1	1.653162221
2011	12	74685106.47	7.873234004	4.987531131	39.2679997	0.1	1.44345709
2011	12	69319256.34	7.840853895	6.657359723	52.19938491	0.1	1.48513707
2011	12	65497869.98	7.816227177	19.83055147	155.0000954	0.1	1.655896532
2011	12	75469902.48	7.877773789	7.559584995	59.55270053	0.1	1.504839374
2011	12	108002838.3	8.033435169	3.657770952	29.38446581	0.1	1.402205867
2011	12	107458058	8.031238988	5.211583143	41.85546972	0.1	1.45269761

Date	Time (t)	$\left(GGP - \left(\frac{CIC}{G}\right)\right)^2$	LOG$\left(GGP_d - \left(\frac{CIC}{G}\right)\right)$	Stability Monitor, SM (Absolute Value)	SM × LOG$\left(GGP_d - \left(\frac{CIC}{G}\right)\right)^2$	Weights (W)	lSMl × LOG$\left(\left(GGP_d - \left(\frac{CIC}{G}\right)\right)^2\right)^{0.1}$
2011	12	157260691.6	8.196620181	8.65133443	70.91170239	0.1	1.531340579
2012	13	135281630.7	8.13123883	2.236476618	18.18532551	0.1	1.336509678
2012	13	109445310.4	8.039197157	2.352874454	18.91522162	0.1	1.341779472
2012	13	120118052.4	8.079608282	6.823907538	55.13449986	0.1	1.493283767
2012	13	126345421.1	8.101559507	0.075578359	0.612302569	0.1	0.952130787
2012	13	132717678.8	8.122928777	0.716674813	5.821498459	0.1	1.192623819
2012	13	127595563.5	8.105835574	4.482991242	36.33838989	0.1	1.432308496
2012	13	134301191	8.128079864	0.675927884	5.493995821	0.1	1.185738257
2012	13	139084047.5	8.143277321	3.821580957	31.12019353	0.1	1.410276369
2012	13	143793104.6	8.157738061	8.656040025	70.61370716	0.1	1.530695838
2012	13	169002738.3	8.227893741	0.779322351	6.412181491	0.1	1.204205428
2012	13	213863873.2	8.330137428	1.486637672	12.38389611	0.1	1.286132551
2012	13	276125053.5	8.441105813	1.827330953	15.42469393	0.1	1.314684842
2013	14	224741035.3	8.351682377	0.424100347	3.541951392	0.1	1.134812889
2013	14	198935293	8.298711838	3.562020246	29.56017958	0.1	1.403042113
2013	14	212350013.7	8.327052293	1.17455342	9.780567749	0.1	1.25613526
2013	14	209617557.5	8.321427656	4.302800548	35.80544348	0.1	1.430193848
2013	14	222063401.7	8.346476988	3.242986524	27.06751239	0.1	1.390736441
2013	14	206509010	8.314939005	1.923479496	15.99361468	0.1	1.319455242
2013	14	203817515.6	8.309241504	2.4139587	20.05816582	0.1	1.349674746
2013	14	196783130.4	8.293987865	12.86367423	106.6911579	0.1	1.595191559
2013	14	207772819.6	8.317588733	5.824453064	48.44540518	0.1	1.474094274
2013	14	247602006	8.393754159	0.327678319	2.750451252	0.1	1.106471916
2013	14	282471528.5	8.45097468	0.426925493	3.607936529	0.1	1.136909483
2013	14	415604665.9	8.618680415	14.92864508	128.665221	0.1	1.625347086
2014	15	337290126.1	8.528003628	1.534609213	13.08715294	0.1	1.293256047
2014	15	313505129.2	8.496244651	0.935077563	7.944647743	0.1	1.230289917
2014	15	314047613.4	8.496995497	0.970145765	8.243324201	0.1	1.234838718
2014	15	353561347.8	8.548464781	0.952835437	8.145280172	0.1	1.233362111
2014	15	349995299.9	8.544062212	0.907895485	7.757115503	0.1	1.227354519
2014	15	343826380.6	8.536339196	0.967117346	8.255641708	0.1	1.235023108
2014	15	369427283.4	8.567528966	0.144202313	1.235457492	0.1	1.021369255
2014	15	389133634.7	8.590098771	1.135465693	9.753762452	0.1	1.25579057
2014	15	376456443.2	8.575714735	1.925491118	16.51246255	0.1	1.323674446
2014	15	430587077.4	8.634060992	0.252197348	2.177487284	0.1	1.080925003
2014	15	480683843.4	8.681859525	0.014936601	0.129677475	0.1	0.815241829

Date	Time (t)	$\left(GGP - \left(\frac{CIC}{G}\right)\right)^2$	$\text{LOG}\left(GGP_d - \left(\frac{CIC}{G}\right)\right)$	Stability Monitor, SM (Absolute Value)	SM $\times$ $\text{LOG}\left(GGP_d - \left(\frac{CIC}{G}\right)\right)^2$	Weights (W)	lSMl $\times \text{LOG}\left(\left(\left(GGP_d - \left(\frac{CIC}{G}\right)\right)^2\right)^{0.1}\right)$
2014	15	625659212	8.796337843	0.002866918	0.025218379	0.1	0.692104569
2015	16	624833081.4	8.795764015	0.465507889	4.094497537	0.1	1.151383659
2015	16	507790081.9	8.705684214	1.120181426	9.75194576	0.1	1.255767178
2015	16	532933215.9	8.726672789	1.614674383	14.09073501	0.1	1.302846842
2015	16	521430654.6	8.717196559	1.036317815	9.033786094	0.1	1.2461978
2015	16	513371983.3	8.710432164	0.349636435	3.045484446	0.1	1.117803946
2015	16	507606023.2	8.705526767	1.151673321	10.02592292	0.1	1.259251382
2015	16	515286985.9	8.712049174	0.904476507	7.879843808	0.1	1.229282675
2015	16	529081091.8	8.723522241	0.818797222	7.142795774	0.1	1.217269732
2015	16	532418086.5	8.7262528	0.801228834	6.991725353	0.1	1.214670365
2015	16	696370132.4	8.842840136	0.270820967	2.394826518	0.1	1.091257913
2015	16	709929090.2	8.851214972	2.028039285	17.95061168	0.1	1.334774574
2015	16	956305600.1	8.980596699	1.190586863	10.69218045	0.1	1.267379354
2016	17	801318043.9	8.903804922	0.228398895	2.03361921	0.1	1.073561587
2016	17	713401782.3	8.85333419	0.850462701	7.529430509	0.1	1.22370353
2016	17	720291148	8.857508078	0.714808724	6.331424049	0.1	1.202680142
2016	17	706024551.3	8.848819804	6.779654635	59.9919422	0.1	1.505945629
2016	17	709682933.7	8.851064362	0.663524883	5.872901447	0.1	1.193672727
2016	17	721723088.6	8.858370599	2.131027743	18.8774335	0.1	1.341511175
2016	17	698732662.4	8.844311045	0.63470246	5.613505974	0.1	1.188292672
2016	17	714161861.8	8.853796654	0.284102125	2.515382447	0.1	1.096630707
2016	17	34004003.53	7.531530053	0.692433582	5.215084335	0.1	1.179576559
2016	17	33909592.37	7.530322569	0.101827838	0.766796467	0.1	0.973796054
2016	17	33860587.41	7.529694488	0.302134128	2.274977675	0.1	1.085669692
2016	17	32714174.94	7.514735972	1.208208316	9.079366497	0.1	1.246825152

Supplementary C2 Gold Impact Factor or Cost of Holding Gold in Reserves (Yearly Analysis)

Date	Time (t)	$\left(GGP - \left(\frac{CIC}{G}\right)\right)^2$	$\text{LOG}\left(GGP_d - \left(\frac{CIC}{G}\right)\right)$	Stability Monitor, SM (Absolute Value)	SM $\times$ $\text{LOG}\left(GGP_d - \left(\frac{CIC}{G}\right)\right)^2$	Weights (W)	lSMl $\times \text{LOG}\left(\left(\left(GGP_d - \left(\frac{CIC}{G}\right)\right)^2\right)^{0.1}\right)$
2000	1	626840.6547	5.797157156	0.00	0.00	0.1	0.00
2001	2	943988.0402	5.974966492	1.25	7.49	0.1	1.223121138
2002	3	2172974.441	6.337054618	1.88	11.94	0.1	1.281444596

Date	Time (t)	$\left(GGP - \left(\frac{CIC}{G}\right)\right)^2$	LOG $\left(GGP_d - \left(\frac{CIC}{G}\right)\right)$	Stability Monitor, SM (Absolute Value)	SM × LOG $\left(GGP_d - \left(\frac{CIC}{G}\right)\right)^2$	Weights (W)	|SM| × LOG $\left(\left(GGP_d - \left(\frac{CIC}{G}\right)\right)^2\right)^{0.1}$
2003	4	4036022.09	6.605953535	1.69	11.17	0.1	1.272976542
2004	5	5461996.542	6.737351421	0.64	4.30	0.1	1.156959984
2005	6	6578661.292	6.818137527	2.57	17.51	0.1	1.33143749
2006	7	10876408.57	7.036485513	2.36	16.62	0.1	1.324547483
2007	8	19592635.21	7.292092853	3.89	28.36	0.1	1.397219771
2008	9	34315766.24	7.535493701	4.22	31.78	0.1	1.413224682
2009	10	46099127.92	7.66369271	1.89	14.47	0.1	1.306345985
2010	11	91600999.78	7.961900214	8.15	64.87	0.1	1.517774609
2011	12	162240613.3	8.210159579	1.59	13.04	0.1	1.292831942
2012	13	277452859.2	8.443189205	1.62	13.69	0.1	1.299059462
2013	14	381201399.3	8.581154486	1.04	8.92	0.1	1.244557388
2014	15	561525393.4	8.749369401	1.53	13.41	0.1	1.296386578
2015	16	894943393.4	8.951795566	0.26	2.36	0.1	1.08987382
2016	17	536104704.9	8.729249619	30.05	262.27	0.1	1.745321782

Supplementary D1 Output of the Proposed Optimal Reserve Formula in Comparison with Theoretical Benchmarks (Monthly data Analysis)

Date	Time	Weigh-ted (PV) Results	Weigh-ted (FV) Results	Impact Factor of Gold	Average Inflows into the Economy	Proposed Optimal Reserve TEST	Gross International Reserves	Import Bench Mark	20% of Broad Money (M2)	20% of M2+
2000	1	0.0145	3.9015	0.0000	2847.90	11152.46	123.65		79.20	98.58
2000	1	0.0163	3.9110	1.0831	2962.45	14842.96	131.03		78.58	99.32
2000	1	0.0072	3.9088	1.1211	3235.00	16295.19	154.87		81.18	102.94
2000	1	0.0006	3.9056	1.1488	3547.04	1 7930.08	154.86	14166.78	82.04	105.78
2000	1	0.0457	3.8950	1.3169	6676.97	35104.83	281.62	15261.76	83.66	108.90
2000	1	0.0095	3.8968	1.3467	4169.55	21902.64	181.46	21079.42	85.66	112.94
2000	1	0.0351	3.8895	1.1502	4704.37	23873.70	212.53	22543.11	86.44	117.60
2000	1	0.0527	3.8880	1.1686	5017.21	25634.49	219.24	24355.72	86.28	120.14
2000	1	0.0577	3.8883	1.3910	5152.30	27498.38	235.25	21756.21	89.62	125.40
2000	1	0.0635	3.8871	1.1609	5310.29	27143.61	226.73	23295.38	91.34	128.36
2000	1	0.0682	3.8863	1.1097	5423.26	27464.44	241.65	24244.36	99.92	138.54
2000	1	0.0760	3.8851	1.2547	2413.65	12588.85	235.44	24880.32	106.10	144.96
2001	2	0.0007	3.8589	1.1776	5523.59	27823.61	245.35	19233.19	110.20	149.42
2001	2	0.0020	3.8575	1.0878	5563.09	27522.55	211.82	19567.25	110.76	150.30
2001	2	0.0006	3.8547	1.3556	5649.99	29441.31	229.01	19786.25	111.52	151.24

Date	Time	Weigh-ted (PV) Results	Weigh-ted (FV) Results	Impact Factor of Gold	Average Inflows into the Economy	Proposed Optimal Reserve TEST	Gross International Reserves	Import Bench Mark	20% of Broad Money (M2)	20% of M2+
2001	2	0.0015	3.8538	1.1782	5701.34	28697.30	218.75	26212.87	112.92	152.42
2001	2	0.0031	3.8527	1.5850	5712.40	31079.91	212.09	26491.25	113.34	153.16
2001	2	0.0053	3.8535	1.1080	5713.19	28375.83	212.26	26725.10	114.12	154.22
2001	2	0.0040	3.8557	1.2969	5678.43	29281.76	232.32	26824.08	118.76	159.26
2001	2	0.0019	3.8603	1.1965	5652.36	28593.66	285.77	26788.20	122.86	161.54
2001	2	0.0099	3.8641	1.4018	5655.52	29837.42	263.38	26694.16	130.22	169.70
2001	2	0.0179	3.8666	1.0329	5668.95	27876.54	238.53	26603.84	132.98	179.70
2001	2	0.0257	3.8697	1.4777	5701.34	30633.90	260.97	26589.00	144.88	193.24
2001	2	0.0389	3.8724	1.2160	2586.62	13262.33	286.01	26665.71	157.48	204.96
2002	3	0.0023	3.8565	1.4280	5784.29	30580.53	307.89	20503.23	158.86	208.72
2002	3	0.0148	3.8622	1.4495	5886.99	31357.55	262.61	20683.87	157.56	211.56
2002	3	0.0150	3.8609	1.1217	5954.13	29757.08	333.81	20974.63	158.78	214.90
2002	3	0.0204	3.8578	1.2639	6113.71	31437.56	281.62	27604.80	159.90	215.84
2002	3	0.0268	3.8553	1.2660	6202.98	31933.31	266.42	28120.74	161.74	220.58
2002	3	0.0267	3.8505	1.1952	6240.90	31656.24	317.98	28615.65	166.46	225.20
2002	3	0.0557	3.8390	0.8304	6386.26	30176.12	328.86	29064.78	171.70	233.00
2002	3	0.0341	3.8419	0.6568	6444.72	29213.17	355.93	29491.64	174.34	236.30
2002	3	0.0358	3.8418	1.4540	6454.20	34410.75	341.26	29870.24	178.88	242.72
2002	3	0.0372	3.8407	1.0827	6495.28	32220.40	431.74	30204.31	194.06	260.50
2002	3	0.0403	3.8395	1.2349	6546.62	33484.12	473.68	30375.05	217.82	286.52
2002	3	0.0426	3.8361	1.3402	3137.91	16376.36	537.49	30534.66	236.30	307.36
2003	4	0.0052	3.7975	1.3534	7498.56	38663.34	600.75	23377.90	232.14	308.16
2003	4	0.0018	3.7964	1.4888	6747.28	35672.57	525.27	24949.23	233.56	312.30
2003	4	0.0006	3.7841	1.3361	7574.40	38786.88	587.94	25263.50	229.18	304.20
2003	4	0.0114	3.7620	1.6721	7629.70	41547.55	609.90	34174.72	225.26	302.70
2003	4	0.0137	3.7596	1.4689	6852.35	35921.22	572.22	34380.10	235.80	316.16
2003	4	0.0196	3.7465	1.3791	6865.78	35325.96	769.94	34544.66	240.66	320.86
2003	4	0.0385	3.7223	1.4959	6879.21	36161.73	766.65	33434.83	250.98	325.08
2003	4	0.0002	3.7842	1.1631	6887.11	34074.40	821.85	32259.42	249.64	330.22
2003	4	0.0035	3.7910	1.6991	6890.27	37852.41	873.16	32313.86	245.18	328.92
2003	4	0.0086	3.7880	1.0340	6902.12	33340.80	997.77	32352.21	273.98	359.94
2003	4	0.0341	3.8036	1.3192	6933.72	35756.79	1137.76	32388.10	296.02	385.56
2003	4	0.0882	3.8183	1.2926	3766.90	19584.52	1259.09	32461.09	331.96	423.48
2004	5	0.0840	3.8059	0.9722	7022.99	34146.25	1209.75	25517.85	348.48	429.42
2004	5	0.0703	3.7964	1.2418	7030.10	35913.53	1231.18	25707.15	323.94	423.28
2004	5	0.0614	3.7939	1.5406	7091.72	38266.44	1196.72	25858.10	319.86	424.96
2004	5	0.0629	3.7971	1.4735	7115.42	37949.99	1282.51	33116.85	325.54	429.10
2004	5	0.0773	3.8047	1.3363	7132.01	37217.63	1242.98	33261.61	330.34	437.50
2004	5	0.0830	3.8026	1.2990	7136.74	37001.24	1209.74	33421.22	341.32	449.80

Date	Time	Weigh-ted (PV) Results	Weigh-ted (FV) Results	Impact Factor of Gold	Average Inflows into the Economy	Proposed Optimal Reserve TEST	Gross International Reserves	Import Bench Mark	20% of Broad Money (M2)	20% of M2+
2004	5	0.0856	3.8016	1.3454	7138.32	37352.38	1319.98	33491.74	338.62	449.74
2004	5	0.0820	3.8017	1.5055	7143.06	38495.58	1239.12	33527.62	345.40	455.58
2004	5	0.0794	3.8027	1.1535	7143.06	35969.84	1308.11	33544.95	355.10	465.70
2004	5	0.0810	3.8009	1.3949	7143.06	37692.34	1372.30	33554.84	378.44	493.58
2004	5	0.0925	3.7966	1.2751	7143.85	36891.81	1495.62	33562.27	409.60	520.16
2004	5	0.1019	3.7931	1.1641	3919.54	19829.36	1567.41	33563.50	419.72	533.72
2005	6	0.0407	3.7700	1.3025	7153.33	36576.57	1519.52	27460.64	400.34	517.46
2005	6	0.0382	3.7698	1.0306	7154.12	34615.71	1472.42	27476.73	404.24	521.08
2005	6	0.0423	3.7671	1.1296	7163.60	35380.96	1404.54	27492.81	408.16	526.74
2005	6	0.0380	3.7681	1.0978	7167.55	35149.19	1396.24	33627.84	410.90	537.96
2005	6	0.0353	3.7699	1.1666	7166.76	35632.26	1323.88	33650.11	409.12	537.96
2005	6	0.0447	3.7833	1.2452	7169.92	36374.72	1333.36	33669.91	418.94	542.16
2005	6	0.0479	3.7915	1.2318	7169.13	36355.68	1347.37	33679.81	412.54	542.24
2005	6	0.0657	3.7957	1.2341	7167.55	36521.91	1355.60	33682.28	439.30	560.20
2005	6	0.0863	3.8028	1.5876	7151.75	39168.18	1491.12	33683.52	422.84	540.12
2005	6	0.1032	3.8132	1.1723	7171.50	36493.49	1486.52	33655.06	448.58	574.04
2005	6	0.1036	3.8186	1.3529	7177.03	37859.52	1525.64	33658.77	452.54	575.36
2005	6	0.1322	3.8229	1.2852	4351.53	22802.95	1726.16	33673.62	477.40	608.36
2006	7	0.0899	3.8075	1.5054	7204.68	38925.19	1763.35	28713.65	481.44	613.46
2006	7	0.1168	3.8175	1.3054	7203.89	37746.17	1751.03	28765.62	488.96	627.58
2006	7	0.1322	3.8212	1.5289	7212.58	39541.60	1685.12	28807.69	492.16	630.02
2006	7	0.1428	3.8213	1.5537	7219.69	39837.03	1745.00	33862.92	510.02	656.70
2006	7	0.1603	3.8163	1.2029	7226.80	37431.20	1686.16	33886.43	523.52	667.44
2006	7	0.1431	3.8119	2.0163	7248.92	43285.41	1799.78	33922.31	538.86	681.58
2006	7	0.1610	3.8161	1.9560	7259.98	43074.45	1735.99	33979.23	552.74	692.42
2006	7	0.1487	3.8090	1.1433	7264.72	37057.54	1694.00	34042.33	558.44	704.72
2006	7	0.1464	3.8084	2.4128	7273.41	46314.36	1641.33	34101.72	569.20	721.66
2006	7	0.1395	3.8077	1.4432	7282.89	39257.47	1942.44	34140.07	584.78	749.14
2006	7	0.1497	3.8067	1.4107	7287.63	39113.43	1872.77	34175.95	613.44	788.04
2006	7	0.1902	3.8108	1.2165	3946.23	20589.45	2095.93	34211.84	665.56	846.04
2007	8	0.0240	3.8338	0.9541	7204.68	34667.97	1907.45	30657.27	676.66	855.22
2007	8	0.1350	3.7970	1.0231	7305.01	36197.13	1891.66	30534.78	669.74	845.82
2007	8	0.1404	3.7976	1.4450	7315.28	39377.20	1772.46	30562.00	684.04	856.48
2007	8	0.1476	3.7955	1.3293	7320.81	38597.89	1918.92	34182.14	704.94	894.12
2007	8	0.1874	3.7843	1.1410	7326.34	37457.19	1895.14	34364.02	727.68	918.94
2007	8	0.1475	3.7955	0.9886	7328.71	36142.29	1983.98	34397.43	720.24	904.96
2007	8	0.1541	3.7913	1.2437	7342.93	38103.38	1806.67	34418.46	738.26	935.68
2007	8	0.1501	3.7902	1.2257	7372.16	38084.57	1881.89	34453.10	741.34	965.48
2007	8	0.1554	3.7887	1.5364	7417.98	40654.02	1701.66	34524.87	770.18	1000.00

Date	Time	Weigh-ted (PV) Results	Weigh-ted (FV) Results	Impact Factor of Gold	Average Inflows into the Economy	Proposed Optimal Reserve TEST	Gross International Reserves	Import Bench Mark	20% of Broad Money (M2)	20% of M2+
2007	8	0.1492	3.7799	1.3726	7469.33	39600.30	2308.25	34664.68	822.02	1040.00
2007	8	0.1543	3.7695	1.2874	7564.92	39421.86	2115.63	34862.64	883.58	1060.00
2007	8	0.1504	3.7708	1.4198	5463.15	29178.53	2745.93	35164.54	951.54	1118.60
2008	9	0.0941	3.7494	1.7056	7678.68	42610.14	2536.14	33720.18	916.46	1142.06
2008	9	0.0954	3.7488	1.5565	7702.38	41598.25	2387.00	34048.06	916.02	1154.58
2008	9	0.0971	3.7379	0.9292	7720.55	36781.93	2176.35	34263.34	949.36	1191.84
2008	9	0.0761	3.7249	1.6284	7766.36	42166.65	2157.90	36181.57	951.32	1202.12
2008	9	0.0266	3.6785	1.1983	7854.84	38515.46	2025.89	36318.91	995.46	1250.24
2008	9	0.0011	3.6153	1.3139	8031.01	39595.46	2635.64	36557.71	968.78	1239.40
2008	9	0.0256	3.4868	1.1307	8252.21	38316.03	2127.75	37043.95	1012.02	1303.64
2008	9	0.0803	3.2572	1.4366	8655.10	41320.13	2736.04	37804.88	997.22	1338.96
2008	9	0.0819	3.2655	1.5365	8881.83	43377.78	2552.39	39058.23	1044.24	1386.88
2008	9	0.0877	3.2844	1.4166	9039.03	43285.56	2349.16	40390.78	1073.96	1411.86
2008	9	0.0930	3.3108	1.3171	9227.05	43560.47	2035.47	41623.10	1120.76	1438.88
2008	9	0.0879	3.2852	1.3903	8341.89	39736.02	2442.01	42518.89	1262.26	1612.22
2009	10	0.1153	3.0624	1.3208	9864.57	44374.98	2422.73	43833.48	1216.26	1564.76
2009	10	0.1107	3.0244	1.4331	10361.47	47333.84	2379.37	45126.43	1180.76	1569.34
2009	10	0.1374	2.6187	1.1663	10760.42	42206.07	2384.08	46903.16	1204.26	1642.38
2009	10	0.1124	2.8349	1.1455	11002.15	45029.55	2271.63	48530.80	1216.30	1640.06
2009	10	0.1073	2.7788	1.3398	11232.04	47465.19	2233.22	50312.47	1231.52	1670.02
2009	10	0.0998	2.6626	0.7316	11485.62	40130.51	2479.19	51675.94	1251.40	1731.94
2009	10	0.0983	2.6148	1.2821	11678.38	46658.30	2801.53	52811.76	1260.72	1758.66
2009	10	0.0976	2.6051	1.2960	11636.51	46531.04	2610.30	53870.87	1247.78	1738.90
2009	10	0.0975	2.6037	1.8343	11503.00	52171.79	3373.93	54504.35	1274.54	1745.76
2009	10	0.0972	2.6307	1.4746	11425.59	48016.49	3778.89	54531.57	1373.32	1842.90
2009	10	0.0864	2.7496	1.7844	11314.99	52279.47	4295.61	54135.64	1480.75	1742.26
2009	10	0.0590	3.0336	1.3868	10227.97	45814.85	4524.08	53632.07	1513.38	2046.66
2010	11	0.0412	3.1747	1.1953	11251.00	49630.02	4536.79	52355.27	1554.63	2044.47
2010	11	0.0200	3.3216	1.1444	11273.12	50572.22	4390.74	52081.83	1550.60	2018.81
2010	11	0.0043	3.4839	1.1940	11209.92	52487.68	4688.66	52016.26	1622.39	2107.60
2010	11	0.0242	3.5391	1.7180	11196.49	59130.89	4543.16	52834.03	1605.50	2081.64
2010	11	0.0305	3.5685	1.4316	11860.87	59667.43	4729.11	52748.66	1629.86	2093.41
2010	11	0.0160	3.5640	1.1118	11248.63	52777.38	4914.16	53669.19	1688.14	2169.21
2010	11	0.0381	3.5723	1.2339	11306.30	54769.94	4738.70	53729.82	1661.13	2154.15
2010	11	0.0426	3.5656	1.3523	11311.83	56111.85	4725.56	53901.80	1660.96	2166.00
2010	11	0.0479	3.5739	1.5228	11296.03	58113.69	4727.54	53041.89	1732.38	2234.16
2010	11	0.0653	3.5596	1.3850	12091.55	60577.08	6131.58	53116.13	1916.63	2451.92
2010	11	0.0634	3.5675	1.3980	11321.31	56933.57	6321.26	54345.98	2060.29	2584.96
2010	11	0.0618	3.5757	1.3372	12680.19	63080.02	6743.00	54360.83	2187.02	2732.60

Date	Time	Weigh-ted (PV) Results	Weigh-ted (FV) Results	Impact Factor of Gold	Average Inflows into the Economy	Proposed Optimal Reserve TEST	Gross International Reserves	Import Bench Mark	20% of Broad Money (M2)	20% of M2+
2011	12	0.0378	3.5206	0.9980	11699.71	53307.82	7061.11	57805.91	2133.35	2728.98
2011	12	0.0482	3.5061	1.1745	11845.86	56016.78	7235.84	57192.23	2111.81	2743.98
2011	12	0.0524	3.5005	1.3665	11852.18	58305.57	6756.75	58013.78	2213.10	2866.90
2011	12	0.0452	3.4763	1.3874	11856.13	58201.04	7328.41	55439.73	2270.13	2945.78
2011	12	0.0813	3.5274	1.6532	11860.87	62410.00	7063.34	55684.71	2268.07	2945.58
2011	12	0.1206	3.5688	1.4435	11874.30	60949.59	7162.12	55708.22	2306.49	3040.42
2011	12	0.1378	3.5865	1.4851	11887.73	61927.83	6966.02	55742.86	2319.91	3070.76
2011	12	0.1940	3.6158	1.6559	11916.96	65134.42	6791.27	55792.35	2335.19	3077.74
2011	12	0.1862	3.6172	1.5048	11980.95	63598.12	6968.32	55880.20	2411.91	3170.25
2011	12	0.2130	3.6236	1.4022	12091.55	63344.57	7618.71	56047.23	2623.57	3385.83
2011	12	0.1845	3.6011	1.4527	12142.10	63603.52	7447.99	56366.45	2661.84	3478.67
2011	12	0.1218	3.5589	1.5313	18173.47	94721.21	8428.95	56719.07	2848.13	3638.96
2012	13	0.0788	3.4944	1.3365	12753.55	62617.01	7358.44	70095.34	2766.18	3619.59
2012	13	0.0703	3.4470	1.3418	13141.44	63855.53	7831.76	71132.18	2753.94	3654.09
2012	13	0.0373	3.3783	1.4933	13264.68	65114.66	7792.70	72697.33	2734.50	3700.03
2012	13	0.0071	3.2017	0.9521	13421.09	55843.87	7477.37	61331.62	2838.20	3831.72
2012	13	0.0291	2.7806	1.1926	13704.70	54851.07	7227.52	62377.12	2923.78	3966.28
2012	13	0.1169	1.4397	1.4323	14655.84	43804.94	8053.24	63259.29	2998.92	4079.82
2012	13	0.1214	1.7889	1.1857	14854.92	45990.50	8065.22	65438.13	2972.02	3981.07
2012	13	0.1204	1.8341	1.4103	14924.44	50217.24	8487.99	67683.78	2986.90	4024.58
2012	13	0.1218	1.9825	1.5307	14930.76	54273.10	8403.51	69594.13	3011.90	4082.39
2012	13	0.1223	2.0255	1.2042	14923.65	50024.47	10424.24	70024.70	3108.80	4128.40
2012	13	0.1138	1.4610	1.2861	14827.27	42419.80	10511.20	70132.35	3393.74	4381.64
2012	13	0.1198	1.9169	1.3147	25241.91	84596.30	10046.59	69980.16	3500.66	4523.72
2013	14	0.1438	2.8416	1.1348	14870.72	61270.16	9248.23	92025.97	3410.32	4439.92
2013	14	0.1458	2.8576	1.4030	14889.68	65610.60	8936.59	91943.07	3430.14	4501.16
2013	14	0.1456	2.7457	1.2561	14952.88	62015.39	8729.97	92040.82	3496.24	4598.04
2013	14	0.1455	2.8424	1.4302	15054.79	66514.11	8592.04	70029.65	3558.89	4612.21
2013	14	0.1448	2.9176	1.3907	15170.13	67554.50	7873.04	70317.94	3559.88	4642.68
2013	14	0.1427	3.0093	1.3195	15362.09	68690.54	7822.93	70757.17	3559.92	4660.66
2013	14	0.1412	3.0640	1.3497	15392.11	70109.61	7584.93	71398.08	3588.94	4660.54
2013	14	0.1366	3.0358	1.5952	15426.87	73549.01	8547.60	71926.39	3566.26	4647.22
2013	14	0.1159	2.5707	1.4741	15466.37	64350.27	7583.93	72328.50	3681.94	4799.59
2013	14	0.0912	2.0164	1.1065	15708.11	50486.80	7552.74	72491.82	3876.42	4387.52
2013	14	0.0681	1.4246	1.1369	16434.90	43216.74	8542.54	72986.73	4038.08	5168.76
2013	14	0.0562	1.1964	1.6253	30807.87	88663.71	9637.08	74565.49	4138.28	5387.40
2014	15	0.0856	2.8616	1.2933	18334.81	77748.83	9889.12	101047.63	4202.30	5550.86
2014	15	0.0943	3.4559	1.2303	19325.46	92386.03	9187.81	105161.56	4278.90	5663.55
2014	15	0.1282	4.6111	1.2348	20415.64	121966.01	9882.36	109688.74	4384.66	5842.81

Date	Time	Weigh-ted (PV) Results	Weigh-ted (FV) Results	Impact Factor of Gold	Average Inflows into the Economy	Proposed Optimal Reserve TEST	Gross International Reserves	Import Bench Mark	20% of Broad Money (M2)	20% of M2+
2014	15	0.1375	5.2387	1.2334	21672.51	143244.39	10009.02	90958.12	4439.36	5936.72
2014	15	0.1323	5.3971	1.2274	22618.13	152827.04	11533.71	96185.60	4553.58	6074.28
2014	15	0.1378	5.2667	1.2350	23557.42	156410.58	10348.43	101342.56	4527.82	6147.57
2014	15	0.1360	5.7425	1.0214	23851.30	164570.87	9762.58	106263.19	4651.06	6300.28
2014	15	0.1395	5.9239	1.2558	24213.11	177220.43	9256.61	109675.59	4573.25	6328.73
2014	15	0.1416	6.1193	1.3237	25187.95	191041.35	14372.99	112173.64	4600.47	6410.47
2014	15	0.1472	6.2196	1.0809	25251.15	188064.54	15263.13	114727.38	4888.95	6680.03
2014	15	0.1492	6.1977	0.8152	25255.89	180886.79	15466.45	116919.82	5231.63	6948.72
2014	15	0.1546	6.0753	0.6921	45614.62	315747.24	13907.09	118553.02	5506.01	7368.61
2015	16	0.1753	6.9059	1.1514	25423.37	209300.46	12225.94	146489.82	5504.45	7367.05
2015	16	0.1696	6.9476	1.2558	26550.68	222307.28	11973.21	146759.55	5520.02	7534.68
2015	16	0.1649	7.0606	1.3028	28367.65	241930.19	14102.54	148787.44	5503.88	7677.56
2015	16	0.1546	7.0974	1.2462	30115.89	255930.43	14678.88	125830.66	5615.39	7902.49
2015	16	0.1472	7.2642	1.1178	30755.78	262322.52	13766.36	133180.06	5655.11	8083.35
2015	16	0.1413	7.5271	1.2593	33072.82	295262.11	13493.93	139766.07	5566.42	8293.28
2015	16	0.1616	6.8730	1.2293	27903.93	230595.58	10875.29	147135.27	5427.76	7471.08
2015	16	0.1510	7.2546	1.2173	30484.03	262860.42	12579.69	143670.90	5609.69	7947.20
2015	16	0.1545	7.1924	1.2147	29873.37	255760.49	12047.48	143245.28	5709.57	7905.24
2015	16	0.1569	7.1543	1.0913	29735.12	249845.53	16634.25	138234.33	6236.12	8469.34
2015	16	0.1453	6.7981	1.3348	29938.94	247838.54	17233.36	141102.32	6530.44	8764.46
2015	16	0.1244	6.1978	1.2674	58398.11	443221.39	16708.88	140248.61	6972.18	9291.05
2016	17	0.1442	6.6663	1.0736	30069.29	237069.37	16584.05	178085.80	6966.48	9225.91
2016	17	0.1411	6.7056	1.2237	30583.57	246821.01	15731.82	178609.16	6631.68	8985.64
2016	17	0.1411	6.6571	1.2027	30422.41	243405.84	15514.52	179618.78	6649.94	9066.76
2016	17	0.1433	6.7316	1.5059	30176.72	252906.22	16361.78	142641.49	6832.36	9174.78
2016	17	0.1432	6.7525	1.1937	30108.78	243563.17	14598.42	142809.76	7057.28	9443.60
2016	17	0.1424	6.7869	1.3415	30670.47	253671.87	14129.61	142066.16	6888.02	9291.56
2016	17	0.1445	6.6939	1.1883	31116.81	249767.19	13838.93	142454.66	6946.71	9407.63
2016	17	0.1440	6.7121	1.0966	31164.21	247837.94	12900.22	143927.02	7124.67	9579.10
2016	17	0.1450	6.7733	1.1796	31254.27	253094.53	12998.42	145580.01	7221.68	9668.93
2016	17	0.1462	6.6347	0.9738	31334.84	242989.90	9793.29	146494.36	7651.35	10144.72
2016	17	0.1149	5.7550	1.0857	31376.71	218240.30	9806.37	146835.85	8104.43	10585.29
2016	17	0.0227	4.0231	1.2468	32364.99	171294.43	10115.25	147168.67	8690.49	11338.42

Supplementary D2 Output of the Proposed Optimal Reserve Formula in Comparison with Theoretical Benchmarks (Yearly data Analysis)

Date	Time	Weigh-ted (PV) Results	Weigh-ted (FV) Results	Impact Factor of Gold	Average Inflows into the Economy	Proposed Optimal Reserve TEST	Gross International Reserves	Import Bench Mark	20% of Broad Money (M2)	20% of M2+
2000	1	0.0215	1.8980	0.0000	2414	4633	235.44		106.10	144.96
2001	2	0.0060	1.8942	1.2231	2587	8079	286.01		157.48	204.96
2002	3	0.0073	1.8834	1.2814	3138	9954	537.51		236.30	307.36
2003	4	0.0277	1.8781	1.2730	3767	11974	1259.08	8069.28	331.96	423.48
2004	5	0.0351	1.8705	1.1570	3920	12004	1567.44	9495.17	419.72	533.72
2005	6	0.0530	1.8795	1.3314	4352	14203	1726.20	11884.57	477.40	608.36
2006	7	0.0919	1.8759	1.3245	3946	12992	2095.89	15173.88	665.56	846.04
2007	8	0.0646	1.8638	1.3972	5463	18168	2745.88	19162.64	951.54	1118.60
2008	9	0.0634	1.7058	1.4132	8342	26547	2442.04	24251.71	1262.26	1612.22
2009	10	0.0423	1.7147	1.3063	10228	31332	4524.10	33235.90	1513.38	2046.66
2010	11	0.0150	1.8030	1.5178	12680	42298	6742.93	42138.02	2187.02	2732.60
2011	12	0.0467	1.7977	1.2928	18173	57013	8428.98	53101.24	2848.13	3638.96
2012	13	0.0883	1.5915	1.2991	25242	75192	10046.57	70016.74	3500.66	4523.72
2013	14	0.0403	1.4623	1.2446	30808	84635	9637.03	98707.88	4138.28	5387.40
2014	15	0.1170	2.4920	1.2964	45615	178142	13907.00	128276.49	5506.01	7368.61
2015	16	0.0921	2.5157	1.0899	58398	215932	16708.73	163521.74	6972.18	9291.05
2016	17	0.0179	2.0654	1.7453				202716.14	8690.49	11338.42

Supplementary Table E Calculation of Hard Currency Values (HCV)

date	time	ExR (ghc/us$)	ExR (ghc/GBP)	ExR (ghc/Euro)	$HCV = \frac{ExR_{US/GHc} + ExR_{Euro/GHc} + ExR_{Britain/GHc}}{3}$
2000	1	0.3605	1.0327	0.3663	0.5865
2000	1	0.375	0.6027	0.3732	0.4503
2000	1	0.4095	0.6376	0.3976	0.481566667
2000	1	0.449	0.6946	0.4409	0.528166667
2000	1	0.8452	0.7319	0.4476	0.6749
2000	1	0.5278	0.7934	0.51	0.6104
2000	1	0.5955	0.9161	0.5852	0.698933333
2000	1	0.6351	0.9628	0.5959	0.731266667
2000	1	0.6522	0.9558	0.5961	0.7347
2000	1	0.6722	0.9811	0.595	0.749433333
2000	1	0.6865	0.99	0.6041	0.7602
2000	1	0.6974	1.0086	0.6245	0.776833333
2001	2	0.6992	1.0327	0.651	0.7943

2001	2	0.7042	1.0656	0.6514	0.807066667
2001	2	0.7152	1.0412	0.652	0.8028
2001	2	0.7217	1.0383	0.6467	0.802233333
2001	2	0.7231	1.0368	0.6404	0.8001
2001	2	0.7232	1.0216	0.6205	0.788433333
2001	2	0.7188	1.015	0.6152	0.783
2001	2	0.7155	1.0231	0.6414	0.793333333
2001	2	0.7159	1.0347	0.6489	0.799833333
2001	2	0.7176	1.044	0.6529	0.804833333
2001	2	0.7217	1.044	0.6437	0.803133333
2001	2	0.7287	1.0485	0.6512	0.809466667
2002	3	0.7322	1.0528	0.6536	0.812866667
2002	3	0.7452	1.0656	0.6517	0.820833333
2002	3	0.7537	1.0607	0.6688	0.827733333
2002	3	0.7739	1.1069	0.683	0.8546
2002	3	0.7852	1.1384	0.7168	0.880133333
2002	3	0.79	1.1669	0.7534	0.903433333
2002	3	0.8084	1.4188	0.801	1.0094
2002	3	0.8158	1.2559	0.8035	0.9584
2002	3	0.817	1.2643	0.8062	0.9625
2002	3	0.8222	1.2775	0.8096	0.969766667
2002	3	0.8287	1.2905	0.8247	0.9813
2002	3	0.8393	1.3201	0.8442	1.0012
2003	4	0.9492	1.3541	0.8893	1.0642
